# A unique subset of low-risk Wilms tumors is characterized by loss of function of *TRIM28 (KAP1)*, a gene critical in early renal development: A Children’s Oncology Group study

**DOI:** 10.1371/journal.pone.0208936

**Published:** 2018-12-13

**Authors:** Amy E. Armstrong, Samantha Gadd, Vicki Huff, Daniela S. Gerhard, Jeffrey S. Dome, Elizabeth J. Perlman

**Affiliations:** 1 Division of Hematology-Oncology and Transplantation, Ann & Robert H. Lurie Children’s Hospital of Chicago, Northwestern University’s Feinberg School of Medicine, Chicago, Illinois, United States of America; 2 Department of Pathology and Laboratory Medicine, Ann & Robert H. Lurie Children’s Hospital of Chicago, Northwestern University’s Feinberg School of Medicine and Robert H. Lurie Cancer Center, Chicago, Illinois, United States of America; 3 Department of Genetics, The University of Texas MD Anderson Cancer Center, Houston, Texas, United States of America; 4 Office of Cancer Genomics, National Cancer Institute, Bethesda, Maryland, United States of America; 5 Division of Pediatric Hematology/Oncology, Children's National Medical Center, Washington, District of Columbia, United States of America; University of Bristol, UNITED KINGDOM

## Abstract

This study explores the genomic alterations that contribute to the formation of a unique subset of low-risk, epithelial differentiated, favorable histology Wilms tumors (WT), tumors that have been characterized by their expression of post-induction renal developmental genes (Subset 1 WT). We demonstrate copy neutral loss of heterozygosity involving 19q13.32-q13.43, unaccompanied by evidence for imprinting by DNA methylation. We further identified loss-of-function somatic mutations in *TRIM28* (also known as KAP1), located at 19q13, in 8/9 Subset 1 tumors analyzed. An additional germline *TRIM28* mutation was identified in one patient. Retrospective evaluation of previously analyzed WT outside of Subset 1 identified an additional tumor with anaplasia and both *TRIM28* and *TP53* mutations. A major function of TRIM28 is the repression of endogenous retroviruses early in development. We depleted *TRIM28* in HEK293 cells, which resulted in increased expression of endogenous retroviruses, a finding also demonstrated in *TRIM28*-mutant WT. TRIM28 has been shown by others to be active during early renal development, and to interact with WTX, another gene recurrently mutated in WT. Our findings suggest that inactivation of TRIM28 early in renal development contributes to the formation of this unique subset of FHWTs, although the precise manner in which TRIM28 impacts both normal renal development and oncogenesis remains elusive.

## Introduction

Wilms tumor (WT), the most common renal malignancy in childhood, demonstrates a striking histologic replication of early renal development. Most WTs originate during embryonic development from disruption of mesenchymal-epithelial transition, which in the kidney is known as induction [1–3]. Therefore, increased understanding of the normal early renal developmental processes may elucidate the origins of WT, and *vice versa*. Through analysis of global gene expression patterns, we previously recognized five subsets of WT that differed in their clinical and pathologic characteristics [4]. Subset 1 (S1) (~5% of FHWTs) are exclusively epithelial tumors most commonly detected in infancy; they do not relapse, have a very low incidence of 11p15 loss of heterozygosity (LOH)/loss of imprinting (LOI), and show a post-induction gene expression pattern. In contrast, Subset 5 tumors (S5) (~70% of FHWTs) show a wide range of histologic patterns, arise at a median age of 43.5 months, have a high frequency of 11p15 LOH/LOI, and display the gene expression pattern of pre-induction metanephric mesenchyme. Subsequently, we and others reported recurrent gene mutations in the majority of WT, with specific mutations occurring with different prevalence in different subsets [5–11]. However, these studies (which were confined to high risk WT) did not include the low-risk S1 tumors. In this study we performed comprehensive genomic analysis of S1 tumors, resulting in the identification of recurrent (8/9 patients) mutations in *TRIM28*, a gene previously recognized to be important both in renal development and in carcinogenesis [12]. This work extends the recent report of *TRIM28* mutation in four patients with S1 tumors [13]. Through the analysis of an unselected group of patients with WT, we are able to provide the full clinical context of *TRIM28* mutations in WT, including an additional patient with anaplasia and mutations in both *TRIM28* and *TP53*, and a patient with both germline and somatic TRIM28 mutations. Through the investigation of the functional impact of TRIM28 depletion in HEK293 cells we demonstrate over-expression of endogenous retroviruses (ERVs) and associated zinc finger proteins (ZFPs) following TRIM28 depletion, findings we also document in *TRIM28*-mutant WTs, thereby validating the functional significance of these mutations.

## Results

Of eleven S1 FHWTs previously defined and described [4], DNA was available for nine, and these represent the focus of the current study (**Table 1).** PAJMKN and PAKVET were analyzed within the TARGET initiative using whole exomic sequencing and RNA sequencing. All 9 S1 tumors were included in the TARGET validation set which was analyzed by targeted sequencing for recurrent mutations identified within the TARGET discovery set. The five S1 pilot tumors examined for copy number analysis and methylation analysis are indicated (^a^). All tumors were exclusively of epithelial histology, none had associated nephrogenic rests, and none relapsed.

**Table 1:** Pathogenic *TRIM28* variants identified in nine S1 favorable histology Wilms tumors.

Sample	Age (months)	Initial Detection Method	Genomic Change: hg19 (Protein Change: ENSP00000253024)	Exon	AF	Effect
^a^ PADWNP	18	Targeted seq	g.59058853C>T (p.Gln233x)	4	0.91	Nonsense
^a^ PAJMKN	17	RNAseq	g.59056439_59056440 insCGGCGGGG (p.Asp105fs)	1	1	Frameshift ins
^a^ PAJMZF	8	Targeted seq	g.59060404C>T (p.Arg487x)	12	0.51	Nonsense
PADDLL	6	Targeted seq	g.59060970_59060971 delTT (p.Phe645fs)	13	0.91	Frameshift del
PAJPER	15	Targeted seq	g.59059081G>A	5–6	0.48	Splice site
		Targeted seq	g.59060404C>T (p.Arg487x)	12	0.5	Nonsense
PAKSJN	91	Targeted seq	g.59058844C>T (p.Arg230x)	4	0.97	Nonsense
^a^ PAKVET	13	WES	g.59059081G>A	5–6	0.9	Splice site
^a^ PAJNYM	10	Sanger	g.59056466T>G	1–2	1	Splice site
PAJNID	39	Targeted seq	No variants detected			

AF = allele frequency;

^**a**^ S1 pilot set tumors

### Evaluation of copy number and methylation

**Fig 1:**
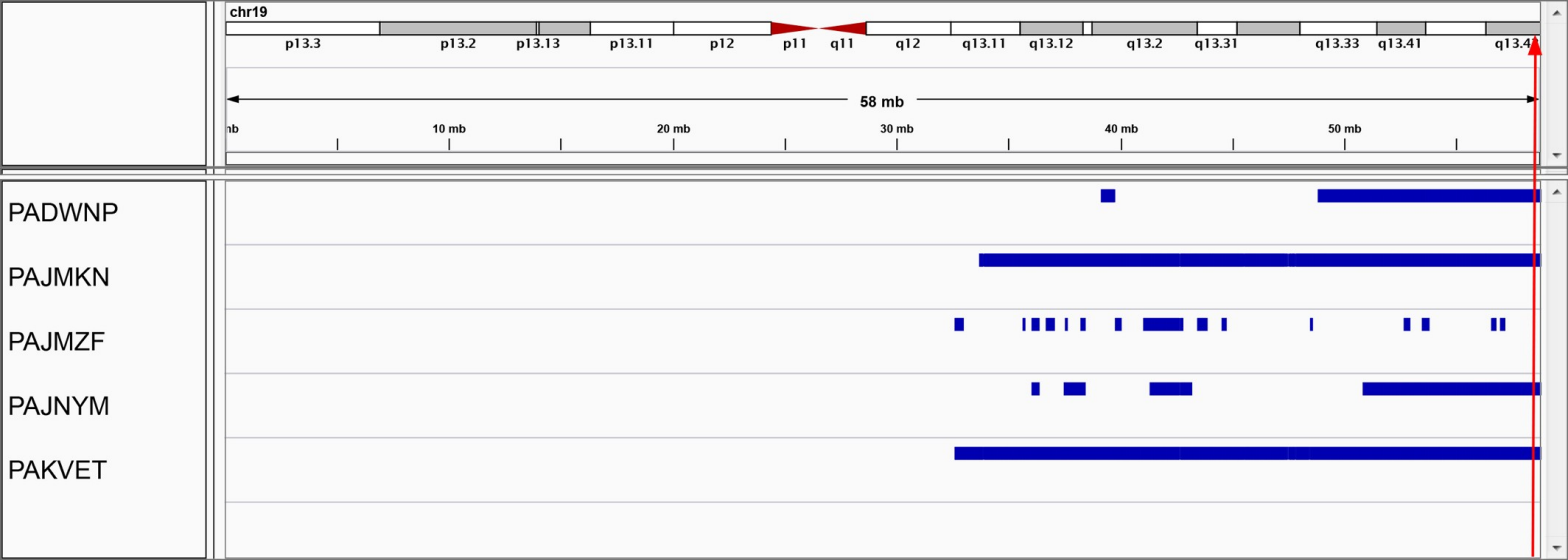
Copy-neutral loss of heterozygosity on chromosome 19 in five S1 favorable histology Wilms tumors. The beta allele frequency values from the Illumina Human 610-quad beadchip were filtered to include only regions on 19q for which the beta value is < 15% or > 85% for ≥ 10 consecutive probes. The filtered files were converted to .bed format and imported into IGV for visualization. The red arrow indicates the location of *TRIM28*. The regions were verified to have normal copy number using BioDiscovery Nexus 6.1 software (see S1 File).

Five S1 pilot tumors were analyzed for copy number and methylation changes. The only recurrent copy number change seen in more than 2 of the 5 tumors was copy neutral loss of heterozygosity (CN-LOH) of chr19q13, present in all five tumors (**S1 Table** and **Fig 1**). All but one tumor (PAJMZF) demonstrated CN-LOH of almost the entire long arm of chromosome 19, 19q13.32 to 9q13.43. For verification, we evaluated the two TARGET cases for which both tumor and normal DNA was characterized by the Affymetrix SNP 6.0 platform; both cases had somatic large regions of CN-LOH (chr19q12-q13.43). We then compared the DNA methylation status of the 5 pilot S1 tumors with 11 clear cell sarcomas of the kidney (CCSK), which are genomically stable and lack CN and/or allelic imbalance on chr19 [14]. Neither hypo- nor hyper-methylation associated with 19q CN-LOH was detected.

### TRIM28 variant identification

No somatic variants were initially identified by WES within the two S1 tumors included in TARGET (PAJMKN and PAKVET). Neither the two TARGET S1 tumors analyzed by WES nor the 9 S1 tumors within the TARGET validation set contained any of the recurrent mutations previously identified in WT [5]. Therefore, we turned to the TARGET RNAseq data performed on PAJMKN and PAKVET. In PAJMKN, a novel internal tandem duplication (ITD) was identified in exon 1 of *TRIM28* (g.chr19:59056439_59056440insCGGCGGGG). We amplified this GC-rich region in the genomic DNA by utilizing PCR enhancer reagents (see S1 File) and submitted the amplicon for Sanger sequencing, which confirmed the ITD to be homozygous (**S1 Fig**). No other variants were identified in either PAJMKN or PAKVET by RNAseq. We assessed the 8 additional S1 tumors for this ITD mutation using Sanger sequencing; none had the ITD but we discovered a nearby splice-site variant between *TRIM28* exons 1 and 2 in PAJNYM (g.chr19:59056466T>G) (**S1 Fig**). Deeper examination of the WES data for PAKVET revealed a novel variant in a conserved splice site between exons 5 and 6 of *TRIM28* (g.chr19:59059081G>A). This variant was also present in the paired normal sample and was therefore not initially detected as somatic. The variant allelic fraction was 61% and 93% in the paired normal and tumor samples, respectively, confirmed by Sanger Sequencing (**S1 Fig**). (This tumor also showed LOH for 19q). Only 22% (18/82) of the RNA reads of the tumor sample retained the normal splice junction, whereas 72% (59/82) were abnormally spliced (**S2 Fig**), resulting in frameshift deletion of 11 nucleotides at position S280.

**Fig 2:**
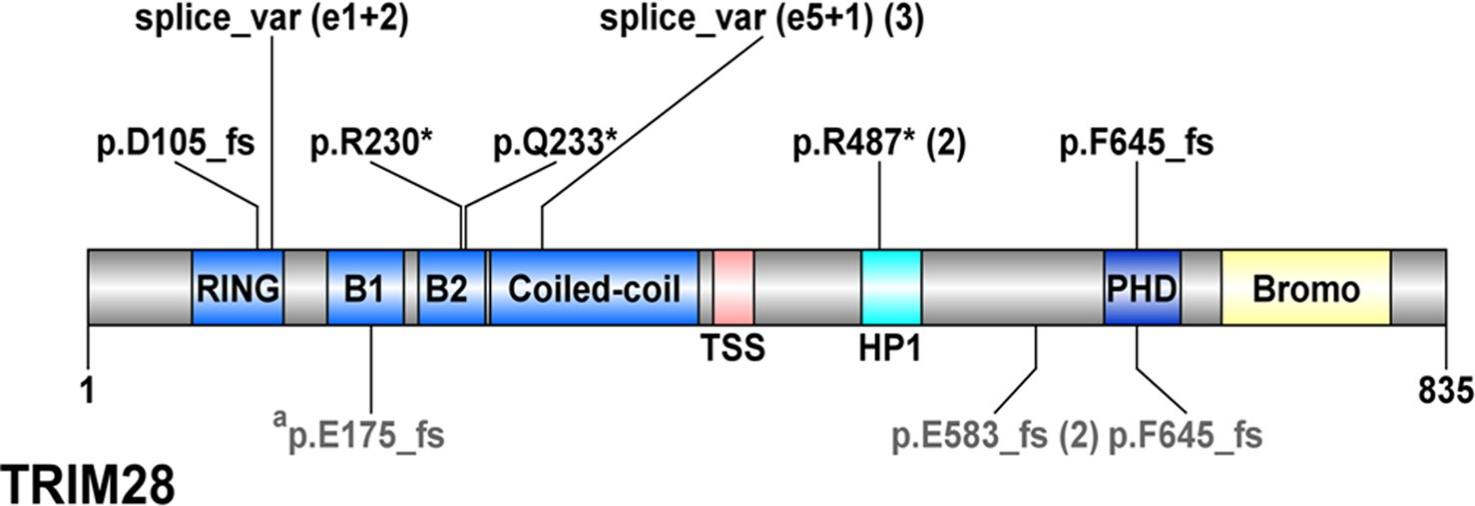
*TRIM28* mutations identified in Wilms tumors. TRIM28 protein structure according to the UniProt database is illustrated using DOG 1.0 software (http://bioinformatics.lcd-ustc.org/dog). TRIM28 includes an N-terminal tripartite RBCC (Ring finger, two B-box zinc fingers, and a coiled coil) domain, which is necessary for interaction with the family of KRAB ZNF transcription factors, a central TIF1 signature sequence (TSS) domain, a HP1 (heterochromatin protein 1)-binding domain, a C terminal combination plant homeodomain (PHD), and a bromodomain. Illustrated are TRIM28 pathogenic mutations identified in 8 S1 tumors and in one anaplastic WT from this study, depicted in black font above the TRIM28 protein image. TRIM28 pathogenic mutations identified by Halliday et al [13] are depicted in gray font below the TRIM28 protein image. One mutation (a) was reported by Halliday et al. [13] in two siblings and their mother.

We next performed targeted sequencing of the entire *TRIM28* gene (*TRIM28* was not included in the original targeted sequencing). High-quality mapped reads were obtained for exons 4 through 17; an adequate read depth could not be achieved for exons 1–3 using targeted sequencing despite several attempts to optimize the primers/sequencing conditions. We successfully performed Sanger sequencing for exons 2 and 3 which revealed no further mutations. Nonsense, frameshift, or splice site mutations were identified in 6/7 tumors analyzed. In total, *TRIM28* mutations were identified in 8/9 S1 tumors tested (**Table 1 and Fig 2**). Of note, 6/8 S1 tumors demonstrated homozygous mutations (AF>90%, or presence of two different mutations). Only 1 tumor had an allelic fraction most consistent with heterozygous mutation, and this tumor (PAJMZF) is the pilot tumor that lacked clear 19p13.43 CN LOH. Methylation analysis of the *TRIM28* promoter in PAJMZF identified five sequential probes with very low beta values in the remaining 4 S1 tumors (average 1.7% +/- 0.7%) and CCSKs (average 1.3% +/- 0.3%), whereas the beta values for PAJMZF were an average of 30%. To determine if this resulted in decreased expression, we performed RT-PCR followed by Sanger sequencing for *TRIM28* in PAJMZF, which demonstrated a ratio of 70:30 wildtype:mutant allele. These findings suggest that hypermethylation of the *TRIM28* promoter in this case is unlikely to be responsible for silencing of the wild-type allele in PAJMZF, as was recently proposed [13].

Lastly, analysis of the sequencing data from the entire TARGET discovery set of 117 high risk WT identified a single patient with *TRIM28* mutation in an anaplastic, epithelial WT that also contained a mutation in *TP53*. This patient had the same conserved splice-site mutation found in PAKVET (g.chr19:59059081G>A), with 93% alternate reads in the tumor sample. Notably, this case had low *TRIM28* mRNA levels (log2 = 6.4) compared to the average expression level in all TARGET cases (log2 = 10.3 +/- 0.8). **Fig 2**provides the *TRIM28* mutation data for all *TRIM28*-mutant WTs, including those recently reported [13].

### TRIM28 depletion effect on gene expression

The genetic expression pattern characterizing S1 tumors compared with other WT subsets has been previously reported [4]. To address the impact of *TRIM28* depletion on gene expression, we knocked out *TRIM28* in the human embryonal kidney HEK293 cell line using CRISPR. We identified three clones showing loss of both *TRIM28* mRNA and protein, and two clones showing *TRIM28* mRNA and protein levels comparable to that seen in the HEK293 parent line (see **S3 and S4 Figs**); a frameshift-causing mutation in *TRIM28* was verified by Sanger sequencing in CRISPR clones with loss of TRIM28 mRNA and protein (**S5 Fig**). The gene expression patterns of the three *TRIM28* knockdown clones were compared to two clones with normal *TRIM28* expression and with the HEK293 parent cell line using Statistical Analysis for Microarrays. We found 20 differentially expressed genes with q < 0.1 (**Table 2**), including *TRIM28* (q < 0.0001; fold change = 0.052) and 18 *ZNF* genes overexpressed in the *TRIM28* knockdown clones. This is in keeping with previous studies demonstrating that essentially all of the top-ranked TRIM28 targets in Ntera2 cells were *ZNF* genes [15]. *APOBEC3B/A*, which is also over-expressed following *TRIM28* depletion, also has an established role in restricting infectivity of certain retroviruses [16]. Only seven of these 20 genes were represented in the Affymetrix U133A array used in the original report of genes defining the S1 subset [4] (identified by an asterisk in Table 2). Of these seven genes, only *TRIM28* was differentially expressed.

**Table 2:** Significant differentially expressed genes in *TRIM28* CRISPR clones.

Gene Name	Location	Score(d)	Fold Change	q-value(%)
APOBEC3B/A*	22q13.1	3.281	7.727	0.000
TRIM28*	19q13.43	-5.102	0.052	0.000
ZNF135*	19q13.43	2.678	15.889	9.627
ZNF28	19q13.41	3.547	4.993	0.000
ZNF347	19q13.42	3.013	4.199	5.946
ZNF354C	5q35.3	3.836	7.745	0.000
ZNF486*	19p12	3.999	4.209	0.000
ZNF528*	19q13.41	4.140	5.327	0.000
ZNF578	19q13.41	2.963	3.564	5.946
ZNF610	19q13.41	2.620	4.702	9.627
ZNF611*	19q13.41	2.609	2.738	9.627
ZNF626	19p12	3.892	5.553	0.000
ZNF677	19q13.42	2.850	7.029	5.946
ZNF681	19p12	5.222	5.075	0.000
ZNF737	19p12	3.156	6.727	0.000
ZNF763	19p13.2	3.074	5.452	5.946
ZNF808	19q13.41	2.963	3.802	5.946
ZNF83*	19q13.41	3.373	9.021	0.000
ZNF850	19q13.12	2.602	2.378	9.627
ZNF883	9q32	4.333	12.126	0.000

* Genes also represented in the original report of genes defining the S1 subset [4]

**Fig 3:**
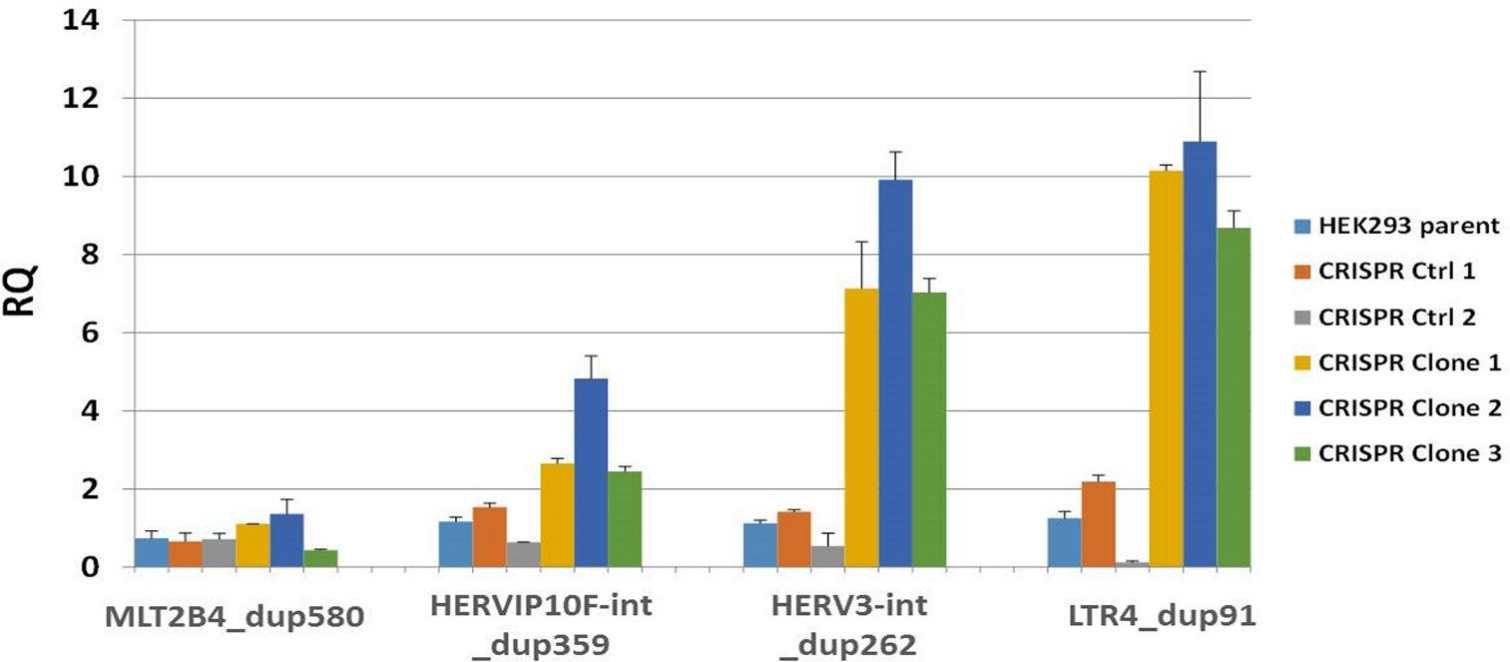
Human endogenous retrovirus expression in *TRIM28* CRISPR clones. The expression of four human endogenous retroviruses up-regulated in *TRIM28*-mutant WT was evaluated in HEK293 cells (HEK293 parent), two CRISPR clones with normal *TRIM28* gene and protein levels (CRISPR Ctrl 1 and 2), and three *TRIM28* knockdown CRISPR clones (CRISPR Clone 1, 2, and 3) by SYBR Green PCR. The endogenous retrovirus levels were normalized to *GAPDH* and are presented as the relative quantitative (RQ) value compared to HEK293 parent cells. Error bars represent the standard deviation of two PCR replicates.

The available TARGET RNAseq data was then used to compare the three *TRIM28*-mutant WT (two S1 tumors and 1 anaplastic tumor) with six randomly selected *TRIM28*-wild-type WT using DESeq2 (adjusted p<0.01, **S2 Table**). *TRIM28* was found to be down-regulated (log 2 fold change (FC) -4.09, adjusted p = 1.16E-15). In addition, the expression of four *KRAB-ZNFs* was increased (*ZNF728* (p = 2.1e-06, log2 FC = 5), *ZNF676* (p = 0.00016, log2 FC = 4), *ZNF208* (p = 0.0003, log2 FC = 4.3), and *ZNF780A*, (p = 0.009, log2 FC 1.7). TRIM28 has been shown to repress transposable elements (TEs) in embryonic stem cells and neural progenitor cells via recruitment by KRAB-ZNF proteins [17–22] and depletion of *TRIM28* results in increased expression of both TEs and ZNFs [21]. Therefore, the TARGET RNAseq data was analyzed for differences in TE expression between the three *TRIM28*-mutant WT (two S1 tumors and 1 anaplastic tumor) and the six randomly selected *TRIM28*-wild-type WT (see methods), revealing differential expression (p<0.001) of 787 TEs overall; 172 of these TEs are classified as ERVs) (**S3 Table**), and 161/172 (94%) of these ERVs are over-expressed in *TRIM28*-mutated tumors, with a median log2 fold change of 6.9. While we were not able to determine global TE expression in *TRIM28* CRISPR clones (Clariom D array lacks probes for these elements), RT-PCR performed on four ERVs differentially expressed in the *TRIM28* mutant WT showed increased expression of 3 of the 4 ERVs within the *TRIM28* depleted clones (**Fig 3**). Lastly, we compared the genes in S2 Table with the 100 genes previously reported to most significantly characterize S1 tumors [4], and identified 18 genes in common (indicated with an asterisk in **S2 Table).** These include low expression of *LEF1*, *MEIS1*, *MEIS2*, *HMGA2*, *SIX2*, and *TRIM28*.

## Discussion

We report recurrent loss-of-function mutations of *TRIM28* in a subset of low-risk epithelial WTs typically arising in infancy. As previously reviewed, pathogenic mutation of *TRIM28* had not been reported prior to its recent recognition in this unique subset of WT [13]. TRIM28 (also known as KAP1, TIF1) was identified as an interaction partner of the family of Kruppel-associated box domain-containing zinc finger transcription factors (KRAB-ZNFs) in 1996 by several laboratories [23–25]. *TRIM28* is critical for early differentiation and development [12]. A number of reports have also associated overexpression of *TRIM28* with aggressiveness or poor outcome in adult cancers, specifically in breast, gastric, pancreatic, and brain tumors [26–29], although the mechanisms proposed are varied and perplexing [30].

TRIM28 is a scaffold protein that recruits chromatin modifying factors (including the histone deacetylase complex NuRD and histone H3 lysine 9-specific methyltransferase SETDB1), thereby establishing repressive histone modifications [31,32]. TRIM28 itself does not bind to DNA, and requires recruitment by KRAB-ZFPs to specific genomic sites [22,23]. The dominant DNA sequences to which the TRIM28 complex is recruited are those coding the KRAB-ZFPs themselves [15]. The best studied function of TRIM28 is its role in silencing transposable elements (TEs) [33,34]. Most TRIM28/KRAB-ZF protein complexes bind to thousands of TEs (particularly to ERVs) in both mouse and human embryonic stem cells [35–40]. ERVs undergo TRIM28/H3K9me3-mediated silencing during the first few days of embryogenesis [12,17]. When these early cells differentiate into various somatic cell types, the H3K9me3 histone-mediated repression is followed by DNA methylation, resulting in stable silencing of endogenous retroviruses (ERVs) [41–43]. Importantly, this TRIM28-induced transcriptional silencing is able to spread over long genomic distances [44]. Many ERVs during evolution have inserted into precise genomic locations, and when these locations are situated near developmental genes, TRIM28-mediated repression may extend to these nearby genes [18]. This has been best exemplified in brain development [32]. TRIM28 depletion in mouse stem cells results in activation of TEs, particularly numerous ERVs, as well as many KRAB-ZFPs [17,21,45]. The presence of increased expression of both ERVs and KRAB-ZNFs in the *TRIM28*-mutant WTs described in the current study raises the hypothesis that failure of TRIM28-mediated repression of ERV and nearby developmental genes early in renal development may result in failure to complete early epithelial differentiation, upsetting the balance between proliferation and differentiation. However, such mechanistic features will need to be clarified in future studies.

Reports are rapidly accumulating concerning the multifunctionality of TRIM28. In addition to its role in repression of ERVs early in development, TRIM28 has also been implicated in imprinting [46]. In addition, TRIM28 is involved in regulating transcription, including polymerase pausing [47]. It has recently been shown to facilitate the recruitment of P-TEFb to promoter-proximal regions allowing for productive transcript elongation, a major mechanism for controlling transcription [48]. Its actions may also be mediated through such factors as long-non-coding RNA [49]. The implication of the involvement of TRIM28 with the super elongation complex (of which P-TEFb is a member) is of particular interest in the context of WT, as mutations in *MLLT1* have been described in a different set of high-risk WTs [11]. MLLT1 has also been implicated in regulation of transcription elongation through its association with PAF, another member of the super elongation complex [11].

TRIM28 is functionally active during early embryonic development. Renal development begins within the undifferentiated metanephric (cap) mesenchyme located at the tips of the ureteric bud. Mesenchymal-to-epithelial transition (MET) occurs within the metanephric mesenchyme following Wnt activation signals provided by the adjacent ureteric bud. MET is followed by differentiation of the epithelial cells into the renal vesicle which then develop into the comma- and S-shaped bodies, a process that requires down-regulation of Wnt4 [1, 50–52]. Proteomic analysis of the developing kidney recently revealed Trim28 to be highly expressed in the undifferentiated cap mesenchyme, however Trim28 was not expressed in the comma- and S-shaped bodies of the differentiating, elongating nephrons. Knockdown of Trim28 results in branching arrest of the ureteric bud, supporting an important role for Trim28 in kidney branching and morphogenesis [53]. TRIM28 has also been shown to interact with WTX, a protein that contributes to β-catenin degradation [54], and a gene inactivated frequently in WT [55]. The WTX-TRIM28 interaction occurs through the N-terminal coiled coil domain of TRIM28 (the same domain responsible for KRAB-ZNF binding and recruitment to chromatin) and through the C-terminal domain of WTX (the same domain responsible for binding WT1) [56]. Individual knock-down of either *WTX* or *TRIM28* result in highly overlapping transcriptomic impacts including both TEs and protein-coding sequences [54]. Kim et al also provide evidence that WTX/TRIM28 is involved in lineage specification through studies of adipocyte and osteoblastic models [54]. It is therefore perhaps not surprising that *TRIM28* mutation would result in abnormal epithelial development specific to that of the nephron. It is intriguing to consider that the loss of the WTX-TRIM28 interaction in this particular context may result in continued Wnt activation (which itself may prevent terminal epithelial differentiation [57], as well as continued proliferation and thereby altering the balance between proliferation and differentiation. TRIM28 depletion in breast and lung cancer cell lines has been shown to result in increased cell proliferation [58]. However, previous characterization of the global gene expression pattern of S1 tumors revealed a lack of Wnt activation in this WT subset [4], and analysis of the RNA-seq data from three TRIM28-mutant tumors in the present study also did not reveal an expression pattern consistent with Wnt activation. Finally, CRISPR-mediated knockdown of TRIM28 in HEK293 cells did not result in up-regulation of Wnt-associated genes. These data indicate that, despite the intriguing association between TRIM28 and WTX, loss of TRIM28 function in S1 tumors does not result in aberrant Wnt activation.

TRIM-28 mutant S1 tumors are of low-risk, with no evidence of recurrences. While this raises questions regarding their malignant potential, our review of the 117 high risk WTs revealed a single *TRIM28* mutation in a WT with diffuse anaplasia. This tumor showed an epithelial histology, was also identified as having a large region of CN-LOH of 19q, and also had a mutation in TP53, a finding that highly correlates with anaplasia in WT [10]. This suggests that while S1 tumors may have an excellent prognosis, this subset, like all other WT subsets, may develop secondary TP53 mutations resulting in the development of anaplasia. Lastly, of the nine S1 tumors sequenced in this study, one patient demonstrated a germ-line mutation with secondary copy-neutral LOH within the tumor, resulting in two mutant alleles. Halliday et al similarly identified germline TRIM28 mutations in two siblings, as well as in the peripheral blood of their mother [13].

In summary, we have identified mutations in *TRIM28* in a unique subset of low-risk epithelial WT and propose that *TRIM28* mutations contribute to aberrant nephron differentiation, resulting in WT formation. We have further shown that knockdown of *TRIM28* leads to upregulation of both KRAB-ZNF genes and endogenous retroviral families. The limitation of this study is that *TRIM28* depletion was performed in a cell line that does not accurately reflect the developmental context of the early developing kidney. Indeed, there are no such cell lines currently available. Therefore, to elucidate specific mechanisms resulting from *TRIM28* mutation requires a developmentally relevant system, such as TRIM28 depletion within renal organoids or conditional depletion in murine models.

## Materials and methods

### Clinical samples

Samples were obtained from patients prospectively registered on the National Wilms Tumor Study 5 (NWTS-5), previously described [5, 59]. Lurie Children’s Hospital Institutional Review Board (IRB) approval for this study was obtained. Informed consent or parental authorization, as appropriate, were obtained as part of the initial sample collection. S1 and S5 subsets of FHWT were previously defined [4]; the current study includes nine of the original 11 S1 tumors for which DNA was available, and six randomly selected comparison S5 tumors. For additional comparison, we used data from previously reported clear cell sarcomas of the kidney (CCSK N = 11) [14]. Two of these 9 S1 tumors were comprehensively characterized through The National Cancer Institute’s “Therapeutically Applicable Research to Generate Effective Treatments” (TARGET) initiative, although they were not included in previous publications which reported only high-risk WT. The sequencing FASTQ files are deposited in the Sequence Read Archive at the National Center for Biotechnology Information, and are accessible through dbGAP, (https://www.ncbi.nlm.nih.gov/projects/gap/cgi-bin/study.cgi?study_id=phs000218) under the accession number phs000471 (See S1 File for sample identification numbers). Chromosome segmental copy number, genotype, sequence analysis (e.g. MAF and summary files), and the clinical information are available through the TARGET Data Matrix (https://ocg.cancer.gov/programs/target/data-matrix). These are annotated within MIAME compliant MAGE-TAB files fully describing the methods, the specimen processing details, and the quality control parameters. The remaining 7 S1 tumors were among the 651 WTs that underwent targeted sequencing in the TARGET validation set for genes recurrently mutated in WT within the TARGET discovery set [5].

### Copy number and methylation analyses

At the time of the original gene expression study [4], a pilot set of five S1 tumors with available tissue were analyzed for copy number and methylation analysis. Copy number analysis was performed using the Illumina Human 610-quad beadchip, as described in S1 File.

Methylation analysis was performed using the Illumina Infinium Human Methylation 450K BeadChips according to the manufacturer’s protocol using methods previously reported [14]. The average of the beta values for probes on 19q in the test set (S1 tumors) was compared with those of 11 CCSKs; regions were identified in which the average beta value for ≥ 5 consecutive probes ranged from 40–60% in the comparison set and ranged from 0–25% or from 75–100% in the test set. The regions were visualized with Integrative Genomics Viewer [60, 61].

### Targeted sequencing of *TRIM28*

Targeted sequencing of the full *TRIM28* gene was performed at GeneWiz (South Plainfield, NJ). In brief, primers were generated against the 17 *TRIM28* exons with flanking regions of ~100 bp plus the 5’ and 3’ UTR. Sequencing was performed using the Illumina MiSeq platform (paired end, 2 x 250 bp). Paired-end fastq files were processed using FASTQGroomer and Trimmomatic and mapped to the human reference genome (hg19) using BWA for Illumina with Galaxy software [62]. Variants were called using the FreeBayes algorithm and were annotated using ANNOVAR and Oncotator [63,64]. Variants detected by either WES or RNAseq were verified using Sanger sequencing using primers and amplification conditions described in S1 File. Sanger sequencing was performed on DNA from all S1 tumors for exons 2 and 3, and on RNA of PAJMZF to evaluate the ratio of the expressed reference and mutant alleles using the primers and amplification conditions described in S1 File. PCR products were purified using the QIAquick PCR Purification Kit (Qiagen, Germantown, MD) and sent to GeneWiz (South Plainfield, NJ) for sequencing.

### Functional analysis of TRIM28 knockout

#### CRISPR

Human embryonic kidney (HEK293) cells were transiently transfected with CRISPR RNA targeting exon 3 of *TRIM28* (crRNA) or non-targeting CRISPR RNA, transactivating RNA (tracrRNA) that forms a complex with crRNA and Cas9, and a Cas9-puromycin resistance expression plasmid (GE Dharmacon, LaFayette CO) using Lipofectamine (ThermoFisher) as described in S1 File. RNA samples from five resulting clones and from the HEK93 parent line were submitted to the NUSeq Core at Northwestern University for Clariom D microarray (ThermoFisher) gene expression analysis. CEL files were imported into Transcriptome Analysis Console 4.0 (ThermoFisher) and the data were processed using Expression (Gene + Exon) analysis type and Gene + Exon–SST-RMA summarization (https://assets.thermofisher.com/TFS-Assets/LSG/manuals/tac_user_manual.pdf). mRNA expression analysis was performed using Significance Analysis of Microarrays (SAM) in R (https://www.r-project.org/). Probes with average log2 < 5.5 in both comparison groups, and probes lacking annotation (ftp.broadinstitute.org/pub/gsea/annotations/Clariom_D_Human.r1.chip) were removed. For each gene, the probeset with the maximum average expression was retained. Two class unpaired SAM was run with the following parameters: nperms = 100, min.foldchange = 0.1, and nvals = 50.

### Gene expression analysis of *TRIM28*-mutant WT

Paired-end fastq files were aligned to hg19 using HISAT2 [65]. Aligned reads were counted using htseq-count with the UCSC transcriptome gtf file as a reference [66]. Differential mRNA gene expression was determined with the DESeq2 package for R (https://www.r-project.org/) using default parameters. Gene Set Enrichment Analysis 3.0 (GSEA, [67]) was performed using a local gmt file containing separate gene lists corresponding to the up- or down-regulated genes from S3 Table in [22]. The preranked gene list was prepared by removing transcripts with basemean < 10 and calculating and ranking the genes based on the–log10 of the DESeq2 p-value from the DESeq2 comparison of *TRIM28*-mutant WT versus *TRIM28*-non-mutant WT. The following GSEA parameters were used: 1000 permutations and classic enrichment statistic.

For transposable element (TE) expression analysis, aligned reads that overlapped with TEs were counted by using htseq-count with a custom hg19 gtf file (http://labshare.cshl.edu/shares/mhammelllab/www-data/TEToolkit/TE_GTF/), which provides a unique ID for each TE annotation. Transcripts with an average read count >5 in all samples were retained. Differential expression analysis was performed using the DESeq2 package, as described above. To detect TEs within the TRIM28 knock-down clones, quantitative RT-PCR was performed for four endogenous retroviruses (ERVs) differentially expressed in *TRIM28* mutant TARGET tumors (adjusted q < 0.05) for which primers lacking high self-complementarity could be generated (See S1 File for primers and conditions).

## Supporting information

S1 Fig Sanger sequencing of TRIM28 variants. (a) Internal tandem duplication in Exon 1 of *TRIM28* (g.chr19:g.59056439_59056440insCGGCGGGG); (b) Single nucleotide polymorphism (SNP) in splice-site between Exon 1 and 2 of *TRIM28* (g.chr19:59056466T>G); (c) SNP in splice-site between Exon 5 and 6 of *TRIM28* (g.chr19:59059081G>A).
(TIF)

S2 Fig Sashimi plot of TRIM28 exon 5-exon 6 boundary in PAKVET. RNAseq paired-end fastq files were processed using FASTQGroomer, mapped to the human reference genome (hg19) using TopHat, and Sashimi plots were generated from the aligned bam file in IGV. The plot demonstrates the effect of the g.59059081G>A DNA splice-site variant on RNA.
(TIF)

S3 Fig *TRIM28* gene expression in CRISPR clones. TRIM28 mRNA levels were evaluated in the parent HEK293 cell line and in selected CRISPR clones by qPCR using the TRIM28 TaqMan Gene Expression Assay from ThermoFisher. TRIM28 expression was normalized to *GAPDH* and is presented as the relative quantitative (RQ) value compared to HEK293 parent cells. Reduced mRNA levels were observed in CRISPR clones d, e and l, and wild-type mRNA levelswere observed in CRISPR clones f and o in comparison to HEK293 parent cells. Error bars represent the standard deviation of two PCR replicates.
(TIF)

S4 Fig TRIM28 protein expression in CRISPR clones. TRIM28 protein levels were evaluated in the parent HEK293 cell line and in selected CRISPR clones by western blotting using a polyclonal TRIM28 antibody from Abcam. The protein content was quantified in cell lysates by BCA, and equal amounts were loaded per lane; B-ACTIN was run on the same blot as an equal loading control. Reduced TRIM28 protein levels were observed in CRISPR clones d, L, and e, whereas protein levels were similar in HEK293 parent cells and clones f and o.
(TIF)

S5 Fig *TRIM28* genotype in CRISPR clones. Genomic DNA was isolated from the parent HEK293 cell line and selected CRISPR clones and the TRIM28 CRISPR target region was amplified for Sanger sequencing. A single base pair insertion resulting in a frameshift change was found in CRISPR clones d, L, and e, whereas the genotype was normal in HEK293 parent cells and clones f and o.
(TIF)

S1 Table Copy number variants and loss of heterozygosity (LOH) identified in S1 favorable histology Wilms tumors. Copy number and LOH analysis were performed in 5 S1 tumors using Nexus 6.1 (BioDiscovery) according to the parameters described in S1 File. Copy number and LOH events were filtered to include only those that occurred in > 2 samples.
(PDF)

S2 Table Differentially expressed genes in TRIM28-mutant Wilms tumors. RNAseq gene transcript data from 3 *TRIM28*-mutant WTs was compared to six randomly selected *TRIM28*-wildtype WTs using DESeq2 as described in the Methods. The data were filtered to include transcripts with adjusted p-value < 0.01.
(PDF)

S3 Table Differentially expressed TEs in TRIM28-mutant Wilms tumors. RNAseq transposable element data from 3 *TRIM28*-mutant WTs was compared to six randomly selected *TRIM28*-wildtype WTs using DESeq2 as described in the Methods. The data were filtered to include TEs with adjusted p-value < 0.001.
(PDF)

S1 File Supplemental methods. (DOCX)
