# Supplementary figures and images for: A unique subset of low-risk Wilms tumors is characterized by loss of function of TRIM28 (KAP1), a gene critical in early renal development: A Children’s Oncology Group study
Source: PLoS One. 2018 Dec 13;13(12):e0208936. doi: 10.1371/journal.pone.0208936 (PMC6292605; doi:10.1371/journal.pone.0208936)

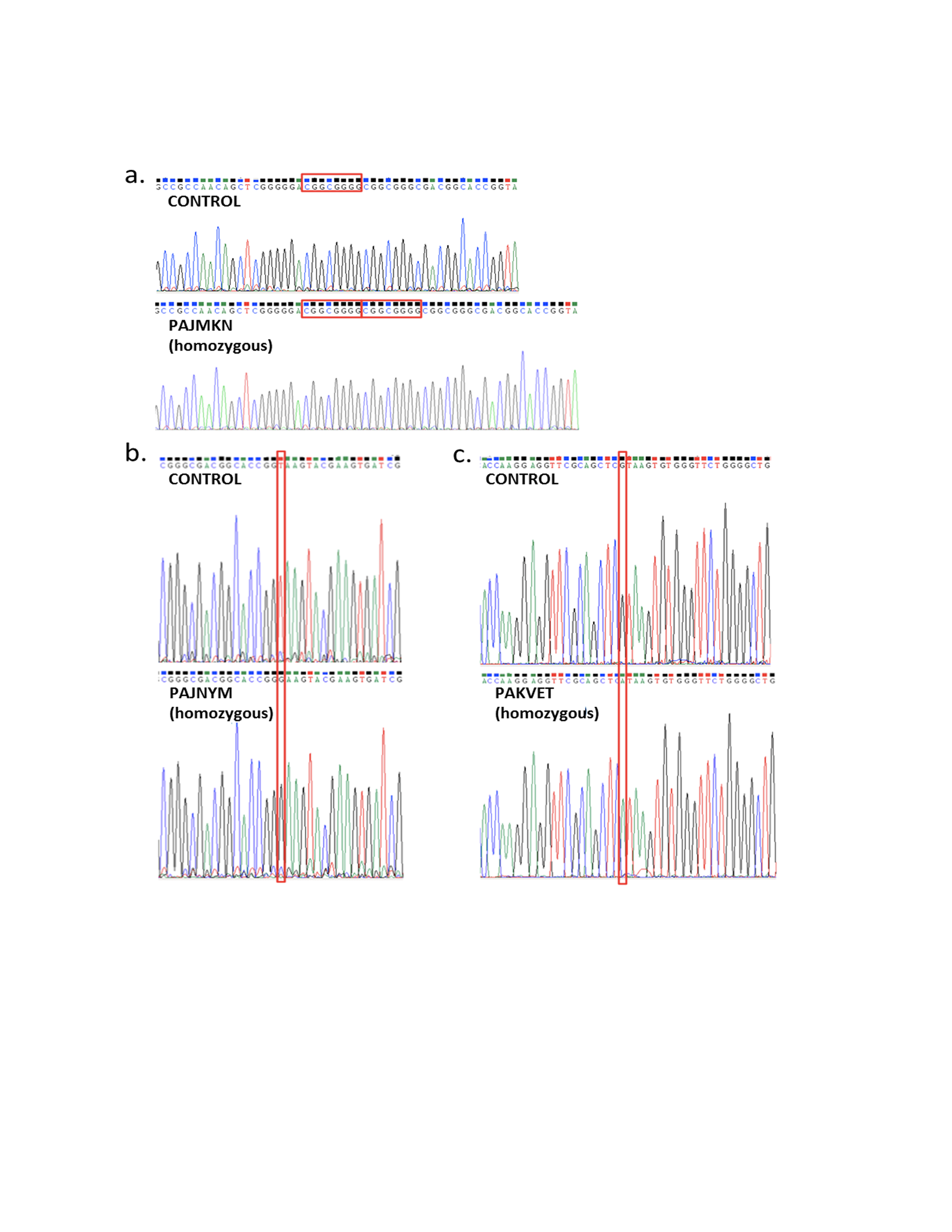

Supplement: S1 Fig — (a) Internal tandem duplication in Exon 1 of TRIM28 (g.chr19:g.59056439_59056440insCGGCGGGG); (b) Single nucleotide polymorphism (SNP) in splice-site between Exon 1 and 2 of TRIM28 (g.chr19:59056466T>G); (c) SNP in splice-site between Exon 5 and 6 of TRIM28 (g.chr19:59059081G>A). (TIF) [file pone.0208936.s001.tif]

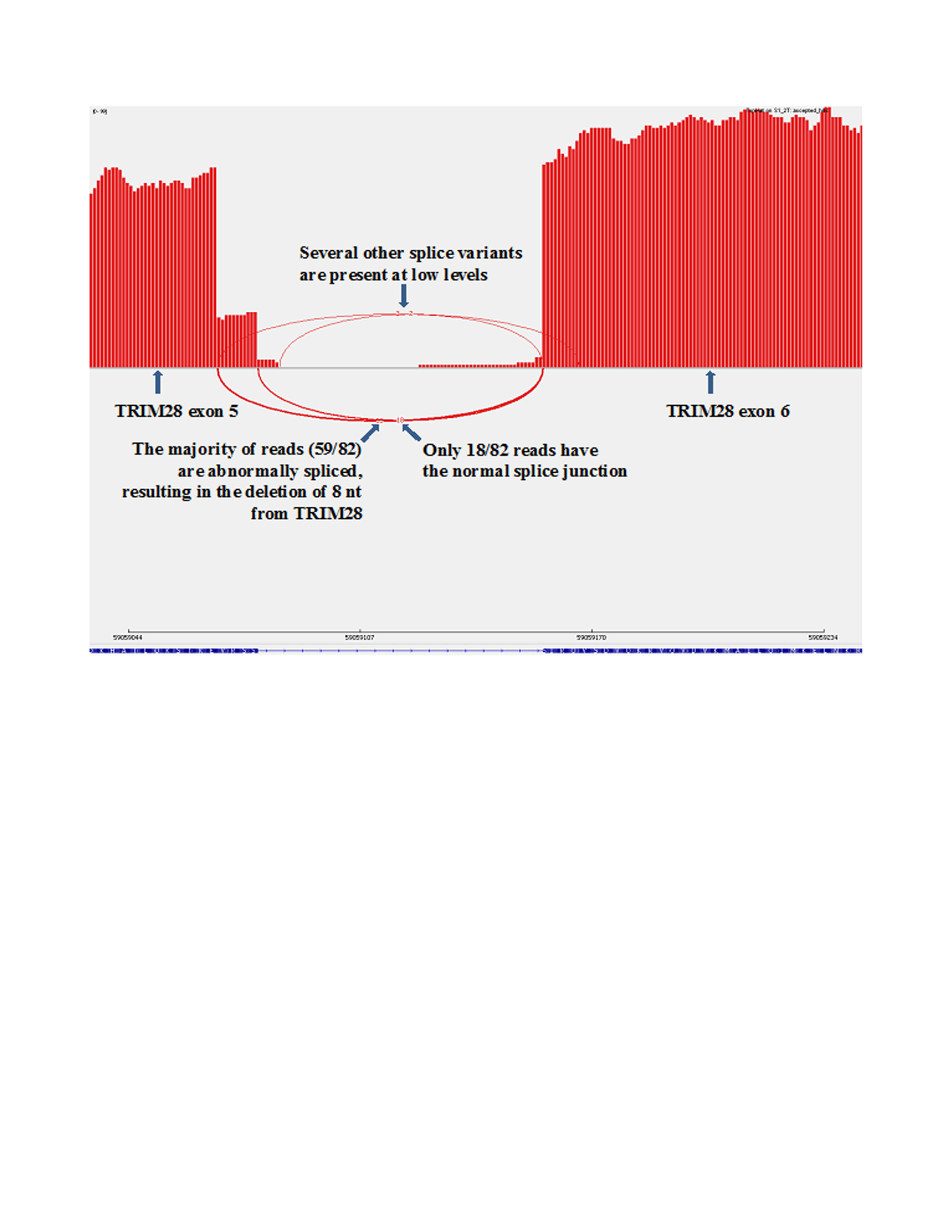

Supplement: S2 Fig — RNAseq paired-end fastq files were processed using FASTQGroomer, mapped to the human reference genome (hg19) using TopHat, and Sashimi plots were generated from the aligned bam file in IGV. The plot demonstrates the effect of the g.59059081G>A DNA splice-site variant on RNA. (TIF) [file pone.0208936.s002.tif]

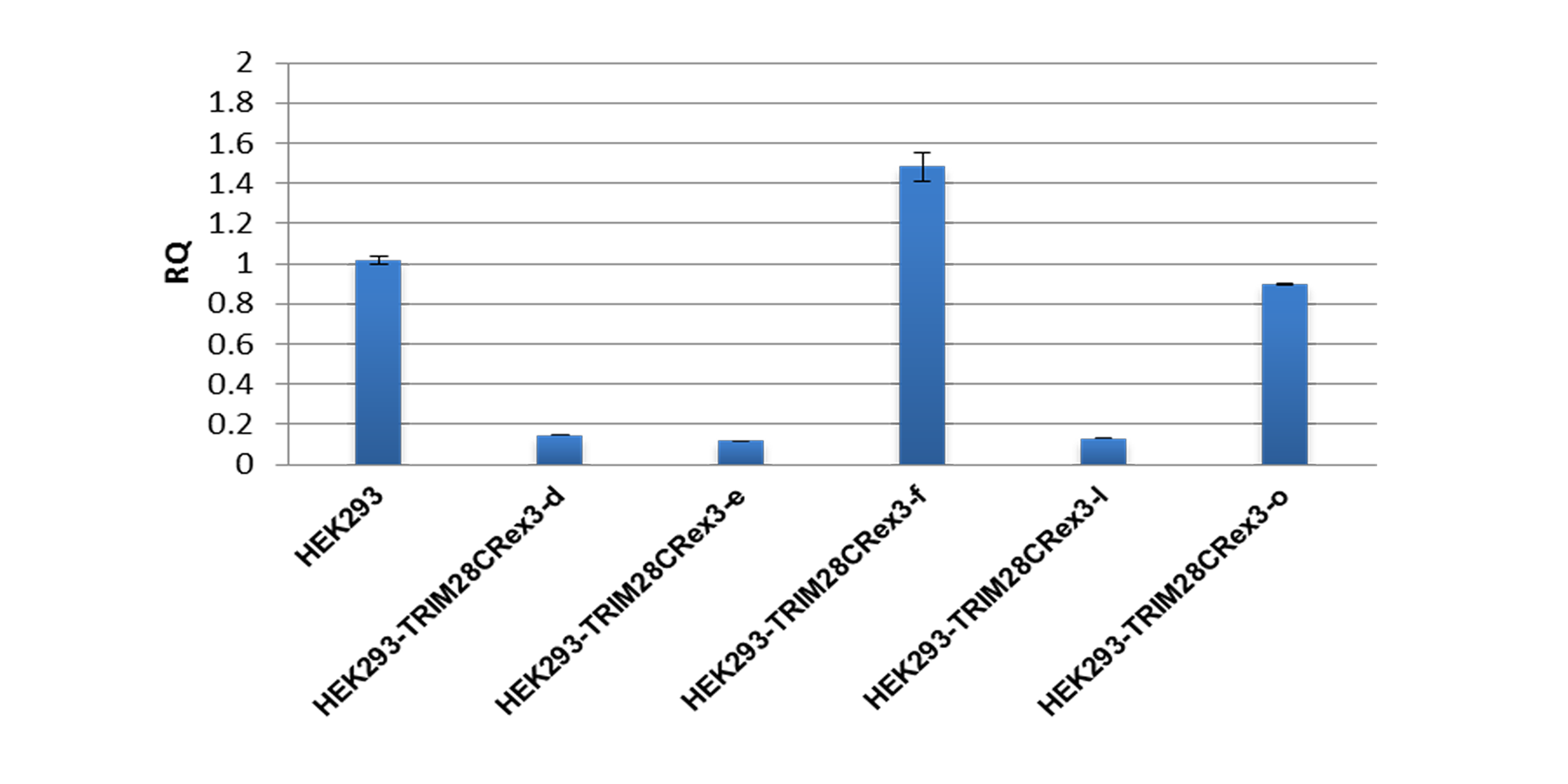

Supplement: S3 Fig — TRIM28 mRNA levels were evaluated in the parent HEK293 cell line and in selected CRISPR clones by qPCR using the TRIM28 TaqMan Gene Expression Assay from ThermoFisher. TRIM28 expression was normalized to GAPDH and is presented as the relative quantitative (RQ) value compared to HEK293 parent cells. Reduced mRNA levels were observed in CRISPR clones d, e and l, and wild-type mRNA levelswere observed in CRISPR clones f and o in comparison to HEK293 parent cells. Error bars represent the standard deviation of two PCR replicates. (TIF) [file pone.0208936.s003.tif]

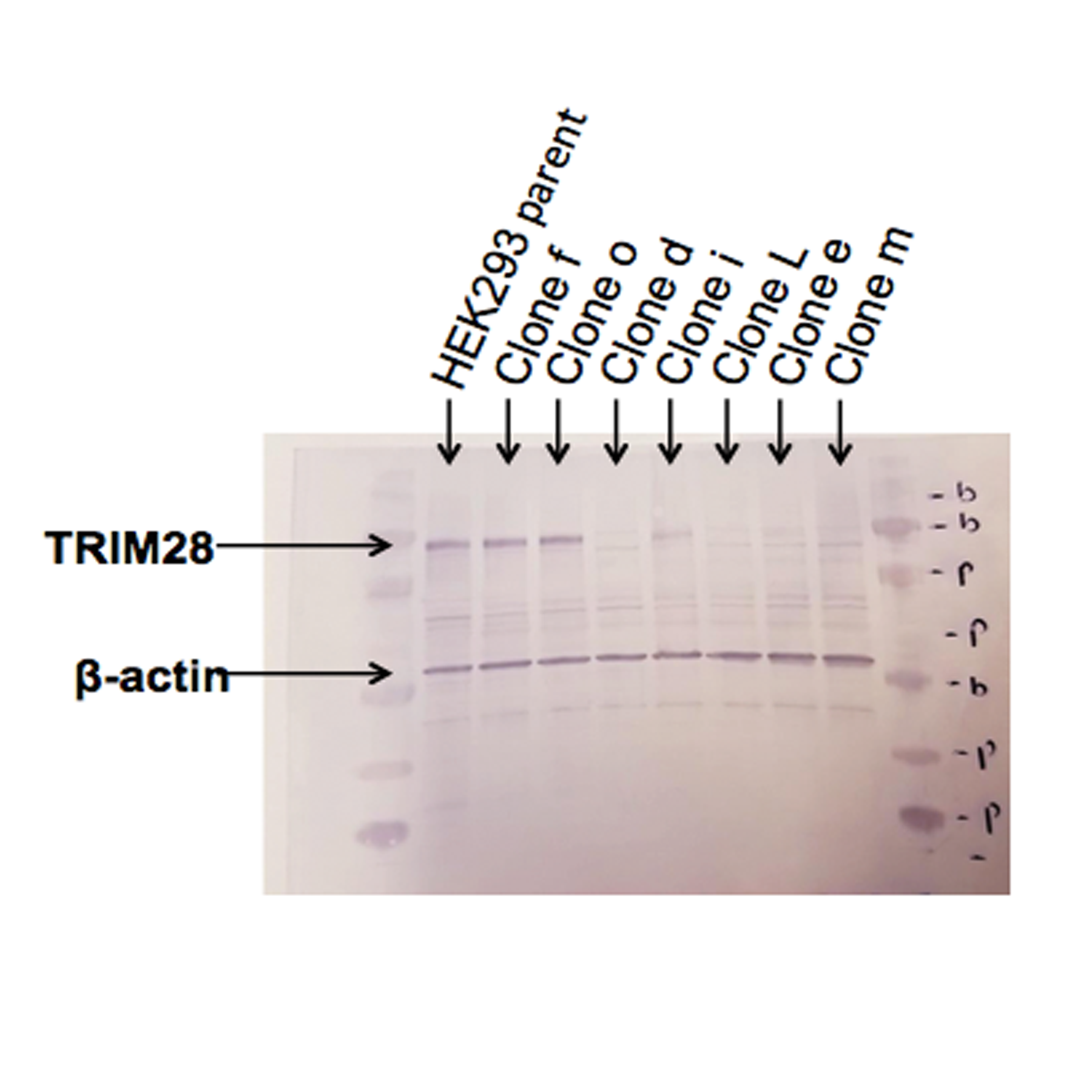

Supplement: S4 Fig — TRIM28 protein levels were evaluated in the parent HEK293 cell line and in selected CRISPR clones by western blotting using a polyclonal TRIM28 antibody from Abcam. The protein content was quantified in cell lysates by BCA, and equal amounts were loaded per lane; B-ACTIN was run on the same blot as an equal loading control. Reduced TRIM28 protein levels were observed in CRISPR clones d, L, and e, whereas protein levels were similar in HEK293 parent cells and clones f and o. (TIF) [file pone.0208936.s004.tif]

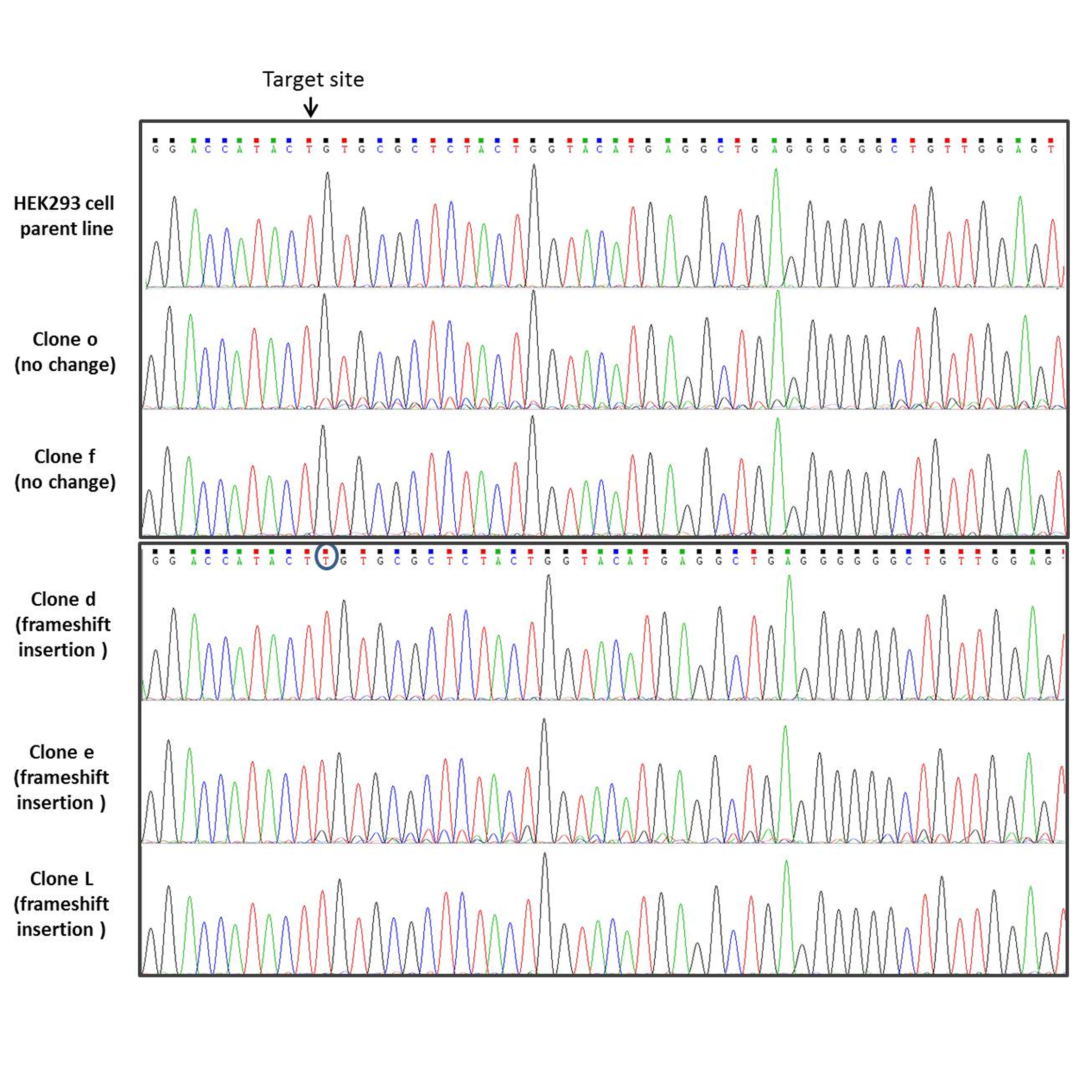

Supplement: S5 Fig — Genomic DNA was isolated from the parent HEK293 cell line and selected CRISPR clones and the TRIM28 CRISPR target region was amplified for Sanger sequencing. A single base pair insertion resulting in a frameshift change was found in CRISPR clones d, L, and e, whereas the genotype was normal in HEK293 parent cells and clones f and o. (TIF) [file pone.0208936.s005.tif]
