# Supplementary material for: A unique subset of low-risk Wilms tumors is characterized by loss of function of TRIM28 (KAP1), a gene critical in early renal development: A Children’s Oncology Group study
Source: PLoS One. 2018 Dec 13;13(12):e0208936. doi: 10.1371/journal.pone.0208936 (PMC6292605; doi:10.1371/journal.pone.0208936)
Supplement: S1 Table — Copy number and LOH analysis were performed in 5 S1 tumors using Nexus 6.1 (BioDiscovery) according to the parameters described in S1 File. Copy number and LOH events were filtered to include only those that occurred in > 2 samples. (PDF) [file pone.0208936.s006.pdf]

Tumor	Chromosome Region (hg19)	Cytoband	Length	Probes	Event
PADWNP S1	chr19:44,100,406-50,917,942	q13.31-33	6817509	429	LOH
PADWNP S1	chr19:50,921,821-55,297,978	q13.33-42	4376158	514	LOH
PADWNP S1	chr19:55,335,172-56,325,458	q13.42	990287	111	LOH
PADWNP S1	chr19:56,327,085-59,114,839	q13.42-43	2792755	318	LOH
PAJMKN S1	chr19:34,739,012-35,799,978	q13.11-12	1060967	151	LOH
PAJMKN S1	chr19:35,808,822-39,374,637	q13.12-2	3565816	348	LOH
PAJMKN S1	chr19:39,382,719-43,252,181	q13.2-31	3869463	367	LOH
PAJMKN S1	chr19:43,952,999-46,887,707	q13.31-32	2934709	347	LOH
PAJMKN S1	chr19:46,893,137-50,884,853	q13.32-33	3991689	443	LOH
PAJMKN S1	chr19:50,917,942-52,158,024	q13.33	1240083	249	LOH
PAJMKN S1	chr19:52,746,709-53,819,218	q13.33-41	1072510	182	LOH
PAJMKN S1	chr19:53,821,449-54,848,493	q13.41-42	1027045	173	LOH
PAJMKN S1	chr19:55,805,448-56,361,796	q13.42	556349	89	LOH
PAJMKN S1	chr19:58,334,188-59,114,839	q13.43	785652	93	LOH
PAJMZF S1	chr19:36,316,822-38,088,587	q13.12	1771766	76	LOH
PAKVET S1	chr19:53,553,268-59,114,839	q13.41-43	5566572	311	LOH
PAJNYM S1	chr19:36,634,054-38,497,788	q13.12-13	1863735	81	LOH
PAJNYM S1	chr19:46,122,746-48,243,670	q13.32	2120897	144	LOH
PAJNYM S1	chr19:48,254,402-52,723,978	q13.32-33	4469577	314	LOH
PAJNYM S1	chr19:52,725,245-59,114,839	q13.33-43	6394595	375	LOH
