# Supplementary material for: A unique subset of low-risk Wilms tumors is characterized by loss of function of TRIM28 (KAP1), a gene critical in early renal development: A Children’s Oncology Group study
Source: PLoS One. 2018 Dec 13;13(12):e0208936. doi: 10.1371/journal.pone.0208936 (PMC6292605; doi:10.1371/journal.pone.0208936)
Supplement: S2 Table — RNAseq gene transcript data from 3 TRIM28-mutant WTs was compared to six randomly selected TRIM28-wildtype WTs using DESeq2 as described in the Methods. The data were filtered to include transcripts with adjusted p-value < 0.01. (PDF) [file pone.0208936.s007.pdf]

UCSC ID	Gene Symbol	baseMean	log2 Fold Change	lfcSE	stat	pvalue	padj
uc011cjk.2	ANP32C	193.5	-3.73	0.404	-9.25	2.25E-20	5.90E-16
uc002mhy.2	CLEC4M*	148.3	6.64	0.728	9.126	7.10E-20	9.30E-16
uc021xwp.1	ANKRD33B	1217	4.24	0.469	9.041	1.56E-19	1.00E-15
uc002dwo.2	RP11-455F5.3	2169.1	3.66	0.403	9.063	1.27E-19	1.00E-15
uc002qtg.1	TRIM28*	2156.3	-4.09	0.455	-9.002	2.21E-19	1.20E-15
uc003tjs.3	PGAM2	1106.3	3.4	0.388	8.752	2.09E-18	9.10E-15
uc002ygw.3	RP4-583P15.11	63.7	4.37	0.528	8.283	1.20E-16	4.50E-13
uc002hrd.1	LRRC37A11P	1181.7	6.59	0.811	8.127	4.41E-16	1.50E-12
uc003dxs.1	RP11-553A10.1	1179.9	5.13	0.648	7.924	2.30E-15	6.70E-12
uc003syn.2	HOXA5*	16364.5	-3.55	0.468	-7.585	3.31E-14	8.70E-11
uc001vge.3	NEK5	100.6	3.39	0.45	7.543	4.60E-14	1.10E-10
uc003fub.1	AC108676.1	499.4	3.86	0.548	7.04	1.92E-12	3.60E-09
uc003fxh.3	PIGZ	173.2	3.41	0.484	7.045	1.86E-12	3.60E-09
uc010xxw.2	AC006262.6	79.3	6.1	0.866	7.04	1.92E-12	3.60E-09
uc003gmb.4	DRD5	483.2	5.71	0.819	6.968	3.23E-12	5.60E-09
uc021uzd.1	ERVV-2	75	6.2	0.894	6.939	3.95E-12	6.50E-09
uc010osh.2	CLCA3P	130	5.45	0.799	6.822	8.97E-12	1.40E-08
uc022aat.2		2248.8	-3.61	0.531	-6.787	1.15E-11	1.70E-08
uc002hwe.4		12.8	5.88	0.868	6.774	1.25E-11	1.70E-08
uc002sqr.3	AC105053.4	67	3.99	0.59	6.76	1.38E-11	1.80E-08
uc010ntg.2	GPR50	684.2	5.6	0.831	6.738	1.61E-11	2.00E-08
uc001oxd.3	UVRAG	65.7	4.24	0.631	6.726	1.74E-11	2.10E-08
uc004djb.1	SSX1	10.5	5.72	0.859	6.656	2.82E-11	3.20E-08
uc001htd.2	DUSP5P1	120.9	4.64	0.7	6.631	3.33E-11	3.60E-08
uc003syx.3	HOXA11	502.5	-3.35	0.512	-6.549	5.79E-11	6.10E-08
uc011dhi.1	BTNL9	14.8	4.1	0.648	6.331	2.44E-10	2.50E-07
uc002wvf.3	RP4-760C5.5	29.6	4.99	0.79	6.316	2.69E-10	2.60E-07
uc001kct.3		103.6	4.23	0.671	6.305	2.88E-10	2.70E-07
uc010jjn.3	C5orf58	14.4	5.03	0.808	6.217	5.08E-10	4.60E-07
uc001uia.2	TMEM132D	76.8	5.13	0.837	6.135	8.52E-10	7.40E-07
uc001dzh.3	KCNC4	79.8	2.2	0.359	6.125	9.08E-10	7.70E-07
uc002yml.3	AL035610.2	130	4.3	0.704	6.101	1.05E-09	8.60E-07
uc021xgp.1	C3orf80	622.2	-2.92	0.484	-6.028	1.66E-09	1.30E-06
uc003mvg.3	TUBB2B*	15121.6	-3.08	0.512	-6.015	1.80E-09	1.40E-06
uc003syp.3	HOXA-AS3	43.2	-3.47	0.579	-5.984	2.18E-09	1.60E-06
uc011mvm.2	MIR503HG	2398.5	3.98	0.666	5.975	2.31E-09	1.70E-06
uc001lpb.3	B4GALNT4	10943.2	-1.7	0.285	-5.972	2.34E-09	1.70E-06
uc003ocm.2	HLA-DMA*	58.1	4.84	0.818	5.911	3.40E-09	2.30E-06
uc002nqz.2	ZNF728	11.3	5.01	0.85	5.902	3.59E-09	2.40E-06
uc002igx.2	FZD2*	17242.3	-1.83	0.313	-5.859	4.66E-09	3.10E-06
uc004exm.4	MIR503HG	591.8	3.47	0.594	5.849	4.95E-09	3.10E-06
uc001izx.3	CCNYL2	164.3	3.41	0.582	5.853	4.84E-09	3.10E-06
uc001wfa.2	TRAJ35	63.3	5.05	0.867	5.827	5.66E-09	3.50E-06
uc002flp.4	PIEZO1	103.4	1.63	0.281	5.793	6.93E-09	4.10E-06
uc031tfr.1	LCN9	19.2	5.53	0.956	5.788	7.11E-09	4.10E-06

uc003ziy.3	INSL4	10.4	5.32	0.921	5.774	7.76E-09	4.30E-06
uc010slt.2	ANP32D	19.3	-4.28	0.742	-5.776	7.64E-09	4.30E-06
uc004dgl.1	RP1-30G7.2	12	4.23	0.734	5.759	8.44E-09	4.60E-06
uc001pmf.1	CRYAB*	2456.6	3.01	0.524	5.75	8.91E-09	4.80E-06
uc010wrt.2	SLC16A5	67.3	2.5	0.437	5.726	1.03E-08	5.40E-06
uc002vjc.1	WNT6	1936.5	4.09	0.716	5.715	1.10E-08	5.70E-06
uc002njy.4	TMEM59L	1153.3	-3.66	0.643	-5.7	1.20E-08	6.00E-06
uc001esj.3	PPIAL4C	54.2	-2.78	0.49	-5.681	1.34E-08	6.60E-06
uc002tnj.2	NIFK-AS1	277.9	3.45	0.609	5.664	1.48E-08	7.20E-06
uc001ijm.3	LINC00707	15.8	3.85	0.682	5.634	1.76E-08	8.40E-06
uc001uii.3	FZD10*	7056.9	-2.74	0.489	-5.605	2.08E-08	9.70E-06
uc003ayg.3	ENTHD1	10.1	3.89	0.694	5.601	2.13E-08	9.80E-06
uc003oyb.2	RCAN2	27.2	-3.79	0.678	-5.596	2.20E-08	9.90E-06
uc004exn.1	LINC00629	377.3	2.62	0.469	5.593	2.23E-08	9.90E-06
uc003dxn.4	LINC00488	624.8	4.46	0.8	5.573	2.50E-08	1.10E-05
uc002uce.4	IFIH1	934.8	3.54	0.636	5.561	2.68E-08	1.10E-05
uc010ckx.3	SLC52A1	15.4	3.9	0.702	5.562	2.67E-08	1.10E-05
uc001lzi.4	OR51E1	6509.1	-4.58	0.825	-5.551	2.83E-08	1.20E-05
uc001ibf.1	KIF26B	5844.1	-2.61	0.471	-5.54	3.02E-08	1.20E-05
uc001ejf.1	RP11-782C8.1	76.8	-3.99	0.723	-5.521	3.36E-08	1.30E-05
uc001fxc.3	ITLN1	80.3	5.02	0.909	5.521	3.37E-08	1.30E-05
uc001ayc.1	ARHGEF19	242.8	-4.3	0.781	-5.507	3.65E-08	1.40E-05
uc001yom.4	TDRD9	44.7	4.89	0.891	5.489	4.03E-08	1.60E-05
uc003epy.3	RAB6B	1978.5	2.3	0.424	5.425	5.80E-08	2.20E-05
uc004axu.3	FOXE1	116	4.38	0.809	5.41	6.29E-08	2.40E-05
uc001sei.3	HOXC13	663.5	4.21	0.778	5.407	6.39E-08	2.40E-05
uc003kei.1	SV2C	36.8	3.54	0.656	5.401	6.63E-08	2.40E-05
uc002avy.3	C15orf59	699.8	3.43	0.636	5.393	6.94E-08	2.50E-05
uc003wwq.3	SGCZ	14.6	-4.73	0.88	-5.379	7.48E-08	2.70E-05
uc021phq.1	RP11-480I12.5	486.8	-2.61	0.487	-5.369	7.93E-08	2.80E-05
uc003vqk.2	KLF14	130.3	-4.3	0.803	-5.357	8.47E-08	2.90E-05
uc001lxa.1	PHLDA2	305.5	3.27	0.61	5.356	8.51E-08	2.90E-05
uc001xtg.1	NGB	22.1	3.99	0.744	5.36	8.34E-08	2.90E-05
uc021yai.1		17.7	-4.73	0.889	-5.324	1.02E-07	3.40E-05
uc010lyr.4		13794.1	-2.2	0.414	-5.323	1.02E-07	3.40E-05
uc001gmr.3	AXDND1	38.5	4.47	0.843	5.306	1.12E-07	3.60E-05
uc009xhz.2	AKR1CL1	7.3	4.27	0.812	5.262	1.43E-07	4.60E-05
uc003gwp.3	SHISA3	17576.2	-3.91	0.746	-5.247	1.54E-07	4.90E-05
uc003sjo.4	ADAP1	31.7	3.41	0.653	5.227	1.72E-07	5.40E-05
uc031roj.1		18.8	4.21	0.808	5.215	1.84E-07	5.70E-05
uc003qwu.1	RP1-137D17.1	99.9	3.32	0.64	5.19	2.10E-07	6.40E-05
uc011bsq.2	ATP13A5	61.6	2.19	0.424	5.174	2.29E-07	6.90E-05
uc003syo.2	HOXA6	2119.8	-3.23	0.626	-5.161	2.45E-07	7.30E-05
uc001axn.3	C1orf64	17.8	4.38	0.849	5.155	2.53E-07	7.40E-05
uc002prt.1	KCNC3	1014	2.98	0.579	5.154	2.56E-07	7.40E-05
uc011cyz.1	LRRTM2*	1221.4	-3.19	0.621	-5.134	2.83E-07	8.20E-05
uc001ibg.1	KIF26B	32.9	-3.61	0.704	-5.128	2.93E-07	8.30E-05

uc001svt.3	RAB3IP	7	4.81	0.939	5.122	3.02E-07	8.50E-05
uc003xhm.3	DUSP4	758.7	-2.08	0.407	-5.11	3.22E-07	9.00E-05
uc003mib.1	FAM153A	146.1	4.09	0.805	5.085	3.67E-07	1.00E-04
uc001dgv.3	RP4-682C21.5	73.9	3.21	0.633	5.062	4.15E-07	1.10E-04
uc003syk.3	HOXA3	162.1	-2.47	0.488	-5.06	4.20E-07	1.10E-04
uc022cix.1	EIF4A1P2	204.5	-1.72	0.34	-5.058	4.24E-07	1.10E-04
uc001hnh.1	HHIPL2	139.9	2.68	0.531	5.054	4.33E-07	1.10E-04
uc002iwq.2	C17orf47	66.8	2.97	0.587	5.054	4.34E-07	1.10E-04
uc003zzb.4	CCIN	505.4	3.29	0.653	5.044	4.56E-07	1.20E-04
uc003hrt.4	HERC5	379.4	3.35	0.666	5.038	4.71E-07	1.20E-04
uc003jck.3	SLC6A3	249.7	3.29	0.653	5.033	4.82E-07	1.20E-04
uc003xtw.1	TOX	4159.6	-1.45	0.288	-5.035	4.77E-07	1.20E-04
uc003cuz.1	PRKAR2A-AS1	170.6	1.69	0.335	5.027	4.99E-07	1.20E-04
uc004elu.3	SLC25A53	857.9	-1.8	0.359	-5.018	5.21E-07	1.30E-04
uc021tmb.1		140.2	2.17	0.434	5.008	5.51E-07	1.30E-04
uc002rca.1	AC011897.1	4455.7	-3.06	0.612	-5.001	5.71E-07	1.40E-04
uc004efg.3	PABPC5	709	-4.06	0.815	-4.974	6.55E-07	1.60E-04
uc002nqs.1	ZNF676	638.4	4.04	0.812	4.976	6.49E-07	1.60E-04
uc001tud.3	OAS1	101.9	3.41	0.687	4.962	6.97E-07	1.60E-04
uc002otk.1	TMEM145	114.6	-2.06	0.417	-4.952	7.36E-07	1.70E-04
uc002pru.1	KCNC3	117.7	2.89	0.585	4.949	7.47E-07	1.70E-04
uc003yve.1	KCNK9	71.9	-3.34	0.676	-4.946	7.58E-07	1.70E-04
uc010ifg.2	TLR6	52.9	1.99	0.403	4.937	7.94E-07	1.80E-04
uc003jak.2	PLEKHG4B	6150.3	-1.97	0.401	-4.928	8.29E-07	1.90E-04
uc001oxs.4	GUCY2EP	5.8	4.61	0.939	4.91	9.13E-07	2.00E-04
uc003tgl.2	TRGV10	18.7	3.43	0.699	4.907	9.24E-07	2.10E-04
uc002kga.3	ZNF750	1717.2	3.38	0.69	4.9	9.57E-07	2.10E-04
uc002new.3	SIN3B	871.1	2.16	0.441	4.899	9.62E-07	2.10E-04
uc003gnd.4		39	3.6	0.737	4.884	1.04E-06	2.20E-04
uc009whu.1		40.1	-4.13	0.847	-4.876	1.08E-06	2.30E-04
uc009wcm.3	SYDE2	306.9	2.91	0.598	4.867	1.13E-06	2.40E-04
uc002uoo.4	PPP1R1C	44	-4.39	0.901	-4.865	1.15E-06	2.40E-04
uc031slz.1	RP11-826N14.2	7.4	3.83	0.786	4.865	1.14E-06	2.40E-04
uc002isl.3	ABCC3	206.2	3.26	0.67	4.865	1.15E-06	2.40E-04
uc003tgm.1		176.7	4.35	0.897	4.85	1.24E-06	2.50E-04
uc002nap.3	EPHX3	22.7	4.38	0.903	4.849	1.24E-06	2.50E-04
uc003syz.1	HOXA11-AS	2677.2	-2.88	0.594	-4.847	1.26E-06	2.60E-04
uc001ooz.2	AP000439.1	7.1	4.65	0.96	4.841	1.29E-06	2.60E-04
uc002tdb.3	FHL2	6.4	3.49	0.723	4.836	1.32E-06	2.60E-04
uc001ihw.2	AKR1C4	6.6	4.07	0.844	4.822	1.42E-06	2.80E-04
uc003fja.1	RP11-33A14.1	57.7	-3.88	0.807	-4.807	1.53E-06	3.00E-04
uc001mmi.3	SOX6	23.9	2.26	0.469	4.805	1.54E-06	3.00E-04
uc001hqe.2		77.1	4.15	0.866	4.799	1.60E-06	3.10E-04
uc002nqq.3	ZNF208	156.6	4.33	0.903	4.798	1.61E-06	3.10E-04
uc001ipg.3	PTPLA	520.8	1.65	0.343	4.792	1.66E-06	3.20E-04
uc010pou.1	RGS1	54.4	2.44	0.51	4.788	1.69E-06	3.20E-04
uc001utw.3	FRY-AS1	31.1	3.38	0.708	4.774	1.80E-06	3.40E-04

uc002ymk.1	AL035610.1	82.1	3.76	0.789	4.767	1.87E-06	3.50E-04
uc010ayn.1		37.8	2.08	0.436	4.763	1.91E-06	3.50E-04
uc003dxy.3	ZBED2	90.7	3.77	0.792	4.756	1.98E-06	3.60E-04
uc021qvz.1	RP11-664H17.1	34.4	3.52	0.741	4.757	1.96E-06	3.60E-04
uc010tye.1	KLC1	244.2	-1.53	0.323	-4.736	2.18E-06	4.00E-04
uc001sxu.3	PHLDA1	26983.6	-1.85	0.392	-4.731	2.23E-06	4.00E-04
uc001etx.3	CA14	4364.1	-2.36	0.499	-4.729	2.25E-06	4.00E-04
uc021urr.1	ZNF208	1943	4.38	0.928	4.718	2.38E-06	4.20E-04
uc003opf.1	LINC00951	45.2	3.53	0.75	4.715	2.42E-06	4.30E-04
uc021qmr.1		11	2.48	0.526	4.716	2.41E-06	4.30E-04
uc010uwo.1	CLDN9	1734.7	-3.44	0.732	-4.699	2.61E-06	4.60E-04
uc011cyf.2	NPY6R	86	2.69	0.574	4.691	2.72E-06	4.70E-04
uc003csq.1	SPINK8	154.2	-3.1	0.662	-4.688	2.76E-06	4.80E-04
uc001jnc.3	DNAJC12	87.4	-2.58	0.55	-4.686	2.78E-06	4.80E-04
uc003uxx.4	VGF	182.8	-3.52	0.757	-4.652	3.29E-06	5.60E-04
uc010clr.1	XAF1	17.7	3.51	0.755	4.644	3.43E-06	5.80E-04
uc009wzp.4	TNNT2	17	4.1	0.884	4.637	3.53E-06	5.90E-04
uc001dqk.3	ARHGAP29*	273.8	3.01	0.65	4.634	3.59E-06	6.00E-04
uc001loy.4	IFITM1	16789.7	2.71	0.584	4.635	3.58E-06	6.00E-04
uc001prh.1	DSCAML1	512.2	2.1	0.454	4.631	3.64E-06	6.00E-04
uc002gdm.1	XAF1	152.7	3.72	0.804	4.631	3.64E-06	6.00E-04
uc002csu.4	CLDN6	1806.9	-3.79	0.822	-4.619	3.86E-06	6.30E-04
uc009xgp.3	KMO	409.2	3.48	0.753	4.614	3.96E-06	6.40E-04
uc002nsx.2		6.7	3.51	0.761	4.608	4.06E-06	6.50E-04
uc003hrp.1	HERC6	21.1	3.9	0.849	4.598	4.27E-06	6.80E-04
uc003pzi.1	CLVS2	172.2	-4.04	0.878	-4.595	4.32E-06	6.80E-04
uc001lbq.1	AFAP1L2	182	2.66	0.579	4.596	4.31E-06	6.80E-04
uc003njl.3	HIST1H2BL	71.9	-2.02	0.44	-4.59	4.43E-06	6.90E-04
uc002ecx.4	HERC2P4	73.1	-3.74	0.816	-4.578	4.70E-06	7.30E-04
uc002yrl.2	IL10RB	37.5	1.83	0.401	4.558	5.18E-06	8.00E-04
uc001zjm.3	MEIS2*	180.5	-3.65	0.803	-4.554	5.27E-06	8.10E-04
uc003qar.3	RSPO3	2758.5	-3.16	0.695	-4.545	5.49E-06	8.30E-04
uc003vop.2	TSPAN33	521.5	1.8	0.395	4.546	5.48E-06	8.30E-04
uc011mvn.2	MIR503	6.8	4.27	0.94	4.545	5.49E-06	8.30E-04
uc001ppv.1	APOA1	490.5	2.77	0.609	4.543	5.55E-06	8.40E-04
uc001gjb.4	ANKRD45	126.7	3.29	0.724	4.541	5.59E-06	8.40E-04
uc002daf.1	TVP23A	12.5	-4.18	0.92	-4.539	5.65E-06	8.40E-04
uc031spd.1		28.1	3.03	0.669	4.53	5.91E-06	8.70E-04
uc009xiw.1	CELF2	18.1	3.05	0.675	4.528	5.96E-06	8.80E-04
uc001bnc.1	SFN	66.1	-3.99	0.881	-4.526	6.02E-06	8.80E-04
uc002rhg.2	DRC1	82.5	2.77	0.613	4.516	6.31E-06	9.10E-04
uc001sfl.3	HNRNPA1	2801.5	-1.4	0.31	-4.517	6.28E-06	9.10E-04
uc002wvy.3	DEFB123	10.4	4.03	0.892	4.518	6.25E-06	9.10E-04
uc004eto.3	GLUD2	705.6	2.26	0.5	4.512	6.41E-06	9.20E-04
uc001mke.3		36.5	2.77	0.614	4.511	6.46E-06	9.20E-04
uc002xpa.3	WFDC11	3.7	4.21	0.934	4.51	6.49E-06	9.20E-04
uc002jje.3	SSTR2	359	3.18	0.708	4.495	6.96E-06	9.70E-04

uc002wsw.2		18.2	4.06	0.904	4.495	6.95E-06	9.70E-04
uc003irk.4	SPOCK3	46.2	-3.2	0.713	-4.493	7.01E-06	9.80E-04
uc001nqx.3	PGA3	6.8	3.59	0.801	4.487	7.23E-06	1.00E-03
uc021tmw.1		457	-3.29	0.733	-4.481	7.45E-06	1.00E-03
uc021sli.1		7.8	3.77	0.843	4.469	7.85E-06	1.10E-03
uc010hel.1	COL6A4P1	13.3	3	0.671	4.467	7.94E-06	1.10E-03
uc003qqm.3	NOX3	56.4	4.16	0.931	4.466	7.99E-06	1.10E-03
uc003ata.3	LGALS2	252.3	3.47	0.776	4.467	7.94E-06	1.10E-03
uc002bpn.4	CRTC3	22.2	1.88	0.421	4.462	8.13E-06	1.10E-03
uc001cpu.2	FAAH	385.9	2.07	0.464	4.456	8.37E-06	1.10E-03
uc003mvi.1		21	-3.36	0.755	-4.454	8.44E-06	1.10E-03
uc002sdt.3	MEIS1*	1474.7	-1.87	0.42	-4.45	8.60E-06	1.10E-03
uc022aqr.1	CSMD1	12.5	-3.66	0.823	-4.452	8.52E-06	1.10E-03
uc001rmg.4	LRRK2	35.4	2.91	0.654	4.45	8.57E-06	1.10E-03
uc002ycw.2	RBBP8NL	153.7	-3.86	0.869	-4.44	9.00E-06	1.20E-03
uc022bje.2		7.1	3.79	0.855	4.436	9.18E-06	1.20E-03
uc001tvl.3	RP11-438N16.1	56.4	-4.04	0.912	-4.433	9.29E-06	1.20E-03
uc010fxn.2	SP140L	14.1	2.5	0.564	4.43	9.43E-06	1.20E-03
uc003bqd.3	EGOT	65.8	3.03	0.685	4.43	9.44E-06	1.20E-03
uc003jnx.3	C5orf28	48.2	1.58	0.357	4.426	9.59E-06	1.20E-03
uc003xxw.1		53.3	-3.33	0.754	-4.419	9.90E-06	1.30E-03
uc002ryc.3	RTN4	15.4	2.87	0.651	4.407	1.05E-05	1.30E-03
uc002chv.1	C16orf13	228.7	-1.81	0.41	-4.405	1.06E-05	1.30E-03
uc002naq.3	EPHX3	5.5	3.64	0.827	4.403	1.07E-05	1.30E-03
uc002ple.1	DHDH	154.7	-2.12	0.482	-4.403	1.07E-05	1.30E-03
uc001kwg.3	CYP17A1	130.6	3.59	0.816	4.397	1.10E-05	1.40E-03
uc004djx.4	EBP	263.9	-1.43	0.326	-4.393	1.12E-05	1.40E-03
uc010bdt.1	PIN4P1	368.2	-2.3	0.524	-4.393	1.12E-05	1.40E-03
uc001xxh.1	EML5	19.7	2.03	0.464	4.386	1.15E-05	1.40E-03
uc003ism.1	RP11-205M3.3	22.9	3.64	0.832	4.381	1.18E-05	1.40E-03
uc011mfv.1		14.7	-4.08	0.932	-4.381	1.18E-05	1.40E-03
uc004axc.1	RP11-498P14.3	76.9	-2.77	0.633	-4.377	1.20E-05	1.40E-03
uc002ilj.3	ITGB3	394.5	2.46	0.563	4.378	1.20E-05	1.40E-03
uc002jxc.3	CBX2	13045.1	-1.58	0.362	-4.373	1.22E-05	1.50E-03
uc003ghm.3	ADRA2C	1806.2	2.1	0.481	4.37	1.24E-05	1.50E-03
uc010sys.2	CCDC42B	31.9	2.46	0.562	4.37	1.24E-05	1.50E-03
uc021vyw.2	TWIST2	235.3	-3.18	0.729	-4.367	1.26E-05	1.50E-03
uc003qek.2		348.2	-3.3	0.758	-4.36	1.30E-05	1.50E-03
uc001rmi.3	LRRK2	129.1	3.23	0.744	4.342	1.41E-05	1.60E-03
uc021yga.1	GPX3*	12473.3	2.41	0.555	4.34	1.43E-05	1.70E-03
uc002ucg.3	GCA	1103.9	2.75	0.634	4.336	1.45E-05	1.70E-03
uc003xil.3	TEX15	2513.3	-2.42	0.559	-4.332	1.48E-05	1.70E-03
uc004ckh.1	LCNL1	16.5	3.19	0.736	4.329	1.50E-05	1.70E-03
uc003dqf.1	ROBO1	123.9	-1.39	0.321	-4.327	1.51E-05	1.70E-03
uc010qig.1	SLC16A9	4902.1	-2.17	0.501	-4.327	1.51E-05	1.70E-03
uc004agt.3	TMEM252	38.3	3.27	0.757	4.325	1.52E-05	1.70E-03
uc001dzv.1	KCNA3	33.5	3.18	0.736	4.32	1.56E-05	1.80E-03

uc010lyq.1	NKAIN3	627.4	-1.83	0.424	-4.315	1.60E-05	1.80E-03
uc002dpd.3	SBK1	27166	-1.5	0.347	-4.313	1.61E-05	1.80E-03
uc003fif.1	RP11-408H1.3	7.1	3.85	0.894	4.303	1.69E-05	1.90E-03
uc001nyc.3	RCOR2	7397.2	-2.22	0.515	-4.302	1.69E-05	1.90E-03
uc002nzf.1	AC002511.1	29	3.05	0.709	4.302	1.70E-05	1.90E-03
uc001fhd.3	EFNA4	336.3	-1.62	0.377	-4.297	1.73E-05	1.90E-03
uc021rgm.1		546.2	3.15	0.734	4.295	1.75E-05	1.90E-03
uc003crm.3		42.7	2.91	0.677	4.294	1.76E-05	1.90E-03
uc011cag.2	IGFBP7	76.7	2.81	0.656	4.292	1.77E-05	1.90E-03
uc021ona.1	RP5-1109J22.1	21.5	2.71	0.633	4.277	1.90E-05	2.00E-03
uc001jut.4	SYNPO2L	88.8	3.19	0.745	4.276	1.90E-05	2.00E-03
uc002fid.3	COTL1	16686.7	-1.63	0.382	-4.277	1.90E-05	2.00E-03
uc010rwp.1	CRYAB*	19.8	3.43	0.803	4.275	1.91E-05	2.00E-03
uc010njt.3	NUDT11	2837.2	-2.5	0.584	-4.274	1.92E-05	2.00E-03
uc001ydj.3	SERPINA12	20.5	3.79	0.887	4.27	1.95E-05	2.10E-03
uc001yrp.1	TMEM121	414.5	-1.91	0.448	-4.27	1.96E-05	2.10E-03
uc001mtv.4	CCDC73	823.5	1.96	0.461	4.256	2.08E-05	2.20E-03
uc010fxm.1	SP140L	455.7	2.18	0.514	4.251	2.13E-05	2.20E-03
uc002jqx.3	FOXJ1	1059.7	2.22	0.523	4.25	2.14E-05	2.20E-03
uc001tmt.3	WSCD2	9.3	3.7	0.872	4.246	2.18E-05	2.30E-03
uc001mbq.1	TRIM5	7.8	3.83	0.903	4.238	2.26E-05	2.30E-03
uc010amc.2	CTD-3006G17.2	36	-3.51	0.828	-4.237	2.27E-05	2.30E-03
uc001swh.2	PTPRR	7	-3.9	0.921	-4.234	2.29E-05	2.30E-03
uc002upt.3		2674.2	2.79	0.658	4.233	2.31E-05	2.40E-03
uc001lep.3	INPP5F	66.3	-3.21	0.759	-4.232	2.32E-05	2.40E-03
uc003weu.2	CNTNAP2	1209.9	-3.03	0.716	-4.228	2.36E-05	2.40E-03
uc002lno.4	HSBP1L1	652.9	1.61	0.381	4.227	2.37E-05	2.40E-03
uc001tuh.3	OAS2	240.7	3.09	0.733	4.216	2.48E-05	2.50E-03
uc002fxx.2	SPNS2	122	2.25	0.535	4.209	2.57E-05	2.60E-03
uc010wnj.1	RP11-670E13.3	5.1	3.35	0.797	4.204	2.62E-05	2.60E-03
uc002maa.2	FSD1	22.1	-2.36	0.562	-4.202	2.65E-05	2.60E-03
uc031qic.1	HMGA2*	549	-2.36	0.562	-4.2	2.67E-05	2.60E-03
uc003qhx.3	NHSL1	227.5	-2.78	0.664	-4.195	2.72E-05	2.70E-03
uc001xds.3	TIMM9	166.6	-1.22	0.292	-4.192	2.76E-05	2.70E-03
uc001zji.2	RP11-122D10.1	53.9	-3.53	0.841	-4.193	2.75E-05	2.70E-03
uc010xrl.1	VSTM2B	1309.1	-3.45	0.823	-4.19	2.79E-05	2.70E-03
uc001rsv.1	DDN	274.7	2.97	0.71	4.186	2.84E-05	2.80E-03
uc002nvo.1	SCGB2B2	117.2	1.6	0.382	4.184	2.87E-05	2.80E-03
uc001yrr.3		145.2	-1.89	0.452	-4.18	2.91E-05	2.80E-03
uc001tuj.3	OAS2	1684.8	3	0.719	4.176	2.97E-05	2.80E-03
uc004eln.1	TMSB15B	51.5	-2.33	0.559	-4.173	3.01E-05	2.90E-03
uc002dpc.1	RP11-57A19.2	593.4	-2.33	0.559	-4.169	3.06E-05	2.90E-03
uc003pym.2	MAN1A1	3341.2	-1.99	0.478	-4.156	3.24E-05	3.10E-03
uc010qzu.2	RP11-451K18.7	5	4.08	0.982	4.151	3.31E-05	3.10E-03
uc009wfr.3	KCNC4	19.9	2.44	0.589	4.147	3.37E-05	3.20E-03
uc031smp.1	HTATSF1P2	462.9	1.98	0.478	4.145	3.40E-05	3.20E-03
uc010emt.3	PPFIA3	9.7	-3.41	0.824	-4.145	3.40E-05	3.20E-03

uc003njn.1	HIST1H2AJ	52.3	-2.74	0.662	-4.14	3.47E-05	3.20E-03
uc001xhb.3	MTHFD1	315.6	-1.57	0.38	-4.138	3.51E-05	3.30E-03
uc010ayu.3	ATP10A	303.4	2.26	0.547	4.127	3.68E-05	3.40E-03
uc003qas.1	RSPO3	141.2	-2.69	0.654	-4.123	3.74E-05	3.50E-03
uc001svx.3	KCNMB4	3068.8	-2.12	0.515	-4.122	3.76E-05	3.50E-03
uc022adx.1	RP13-492C18.2	131	4.15	1.007	4.119	3.81E-05	3.50E-03
uc001uig.2	FZD10-AS1	30.7	-2.71	0.659	-4.12	3.79E-05	3.50E-03
uc001zwr.4	SLC12A1*	32.8	3.13	0.76	4.119	3.81E-05	3.50E-03
uc021sjf.1	ANKRD63	16.6	3.31	0.803	4.116	3.86E-05	3.50E-03
uc002ymf.3	ADAMTS1	11280.7	2.1	0.511	4.116	3.85E-05	3.50E-03
uc003meo.1	GPRIN1	399.1	-1.67	0.406	-4.108	3.99E-05	3.60E-03
uc003hfl.3	AMBN	52.2	-3.92	0.958	-4.094	4.25E-05	3.80E-03
uc002ruo.3	SIX2*	57376.2	-2.39	0.585	-4.091	4.30E-05	3.80E-03
uc002rbz.2	FAM84A	17720.3	-2.81	0.687	-4.089	4.34E-05	3.90E-03
uc003zqz.1		132.7	2.27	0.557	4.081	4.48E-05	4.00E-03
uc003anu.4	MCM5	298.8	-1.42	0.349	-4.08	4.50E-05	4.00E-03
uc003pga.4	RIMS1	23.1	-3.05	0.749	-4.077	4.57E-05	4.00E-03
uc001pfh.3	ENDOD1	2702	1.69	0.415	4.076	4.59E-05	4.00E-03
uc001tzj.2	OASL	21.4	3.08	0.756	4.071	4.68E-05	4.10E-03
uc001fup.2	IGSF9	20.6	-2.39	0.587	-4.069	4.72E-05	4.10E-03
uc002ney.2	SIN3B	65.5	1.71	0.42	4.068	4.75E-05	4.10E-03
uc031sre.1	FRMD1	20.1	3.74	0.921	4.066	4.79E-05	4.10E-03
uc002pwg.3	ETFB	97.2	-2.48	0.61	-4.066	4.78E-05	4.10E-03
uc001eev.3	SYT6	10.1	3.38	0.832	4.062	4.87E-05	4.20E-03
uc002qfu.1	TTYH1	82.1	2.9	0.715	4.061	4.89E-05	4.20E-03
uc004esi.1	RP4-755D9.1	407.5	3.34	0.823	4.059	4.93E-05	4.20E-03
uc003vlq.1	POT1-AS1	42.3	-3.47	0.856	-4.057	4.98E-05	4.20E-03
uc004ejx.3	TCEAL8	50.1	-2.27	0.56	-4.056	4.99E-05	4.20E-03
uc001kbg.1	MBL1P	109.3	-2.02	0.499	-4.051	5.10E-05	4.30E-03
uc001qgg.4	ADAMTS8	109.4	2.11	0.522	4.047	5.20E-05	4.40E-03
uc011ats.1	TATDN2	58.7	-2.77	0.686	-4.044	5.25E-05	4.40E-03
uc002ozs.3	CBLC	8.8	-3.79	0.938	-4.044	5.26E-05	4.40E-03
uc002jds.1	SCN4A	487.1	2.66	0.657	4.041	5.32E-05	4.50E-03
uc010ebt.2	CRLF1	658.2	-2.92	0.722	-4.037	5.42E-05	4.50E-03
uc002dhe.3	ACSM5	58.6	3.45	0.854	4.036	5.45E-05	4.50E-03
uc001dil.1	RP11-183M13.1	11.3	3.56	0.882	4.035	5.47E-05	4.50E-03
uc001eob.1	ANKRD35	220.4	-2.9	0.72	-4.033	5.51E-05	4.50E-03
uc011irz.2	C6orf48	31.7	-2.25	0.557	-4.033	5.51E-05	4.50E-03
uc004cgv.4	NACC2	14.2	2.36	0.587	4.025	5.69E-05	4.70E-03
uc004bwg.3	CCBL1	31.1	2.43	0.605	4.024	5.73E-05	4.70E-03
uc001bvl.4	ZBTB8B	265	-1.28	0.319	-4.022	5.77E-05	4.70E-03
uc002arn.2	SPESP1	349.1	-3.69	0.919	-4.018	5.86E-05	4.80E-03
uc003ijt.3	RP13-539F13.3	6.6	3.23	0.808	4.004	6.23E-05	5.10E-03
uc010nyk.2	TAS1R3	73.6	2.47	0.619	4.001	6.31E-05	5.10E-03
uc009vxf.1	KLF17	10.5	3.33	0.833	4.001	6.31E-05	5.10E-03
uc001dql.3	ARHGAP29*	32.8	3.2	0.8	3.999	6.35E-05	5.10E-03
uc001eiw.1	RP11-782C8.1	194.9	-2.58	0.644	-3.999	6.36E-05	5.10E-03

uc001ooc.3	MTL5	412.5	-2.29	0.575	-3.993	6.51E-05	5.20E-03
uc002ozt.2	BCAM	2712.1	1.76	0.44	3.993	6.53E-05	5.20E-03
uc003oph.1	LRFN2	334.1	2.85	0.713	3.991	6.58E-05	5.20E-03
uc003bak.1	CSDC2	1630.1	2.26	0.566	3.985	6.74E-05	5.30E-03
uc021rat.1		9.4	2.72	0.684	3.981	6.85E-05	5.40E-03
uc031psc.1	LINC00210	33.2	3.22	0.81	3.978	6.96E-05	5.50E-03
uc001cgh.2	KCNQ4	4.7	3.21	0.808	3.972	7.12E-05	5.60E-03
uc002aez.2	AQP9	7.3	3.6	0.905	3.972	7.11E-05	5.60E-03
uc002ymj.3	LINC00314	8.9	3.44	0.867	3.97	7.17E-05	5.60E-03
uc002prx.3	NAPSA	159.9	2.2	0.555	3.961	7.45E-05	5.80E-03
uc001ssz.1	RP11-366L20.2	1843	-1.99	0.501	-3.96	7.51E-05	5.80E-03
uc010shy.2	LMO3	3.3	3.69	0.934	3.958	7.55E-05	5.80E-03
uc001gde.2	LRRC52	110.9	2.76	0.697	3.955	7.64E-05	5.90E-03
uc001lsw.2	MUC6	1475.2	-3.05	0.771	-3.95	7.81E-05	6.00E-03
uc001ouo.3	DNAJB13	100.8	2.48	0.629	3.946	7.93E-05	6.10E-03
uc003jro.1	GAPT	128.1	2.75	0.696	3.945	7.98E-05	6.10E-03
uc004bfg.2	ZNF483	60.9	-1.53	0.388	-3.944	8.00E-05	6.10E-03
uc001jur.4	MYOZ1	321.9	2.73	0.691	3.943	8.05E-05	6.10E-03
uc001xfd.3	MNAT1	225.1	-1.09	0.276	-3.936	8.28E-05	6.30E-03
uc002nsc.1	CTD-2081K17.2	73.2	2.49	0.634	3.931	8.45E-05	6.40E-03
uc002lka.3	CDH7*	45.9	-2.84	0.723	-3.926	8.65E-05	6.50E-03
uc003bng.3	AC000036.4	27.6	3.04	0.775	3.922	8.79E-05	6.60E-03
uc011jzm.2	HOXA10	2095.9	-2.03	0.517	-3.919	8.89E-05	6.70E-03
uc011cfk.2	LEF1*	38.3	-2.5	0.639	-3.917	8.97E-05	6.70E-03
uc010qzq.2	OR52E6	3.1	3.78	0.966	3.916	9.00E-05	6.70E-03
uc003cwv.4	TCTA	2864.9	1.51	0.385	3.913	9.12E-05	6.70E-03
uc003gkk.3	ABLIM2	7.3	3.38	0.864	3.914	9.08E-05	6.70E-03
uc003jfm.4	ANKH	8875	1.37	0.35	3.913	9.12E-05	6.70E-03
uc002ymh.3	AP001604.3	19.7	2.7	0.689	3.915	9.06E-05	6.70E-03
uc010yey.2	TTYH1	3.3	3.8	0.973	3.908	9.31E-05	6.80E-03
uc002oes.2	CTD-2162K18.4	455.3	1.78	0.457	3.907	9.34E-05	6.80E-03
uc011hek.2	HLA-DRA	25.9	2.59	0.664	3.902	9.55E-05	7.00E-03
uc001pcb.3	PRSS23	2483.9	2.31	0.592	3.9	9.63E-05	7.00E-03
uc001tug.3	OAS3	7274	2.94	0.755	3.898	9.69E-05	7.00E-03
uc002mok.2	PDE4A	7.3	3.07	0.787	3.897	9.73E-05	7.00E-03
uc010fbp.3	NRXN1	69.9	-3.19	0.82	-3.89	0.0001001	7.20E-03
uc022aqz.2		9.7	-3.3	0.849	-3.885	0.0001022	7.40E-03
uc010stv.2	TRHDE-AS1	266.9	2.96	0.761	3.884	0.0001027	7.40E-03
uc001xye.1	KCNK13	89.5	2.39	0.616	3.884	0.0001029	7.40E-03
uc003ymd.3	ZFPM2	32.5	2.85	0.735	3.881	0.0001041	7.40E-03
uc003nhh.3	HIST1H2BH	1048.2	-2.24	0.578	-3.879	0.0001047	7.40E-03
uc001yqb.2	CDCA4	1654.1	-1.32	0.34	-3.88	0.0001045	7.40E-03
uc001bpr.3	ATPIF1	3621.7	1.44	0.371	3.876	0.0001064	7.50E-03
uc010yfy.1	RPL28	241.9	-1.94	0.5	-3.876	0.000106	7.50E-03
uc002wxg.2	CCM2L	15.3	-2.86	0.737	-3.876	0.0001061	7.50E-03
uc003fpm.3	LIPH	12.7	2.58	0.667	3.871	0.0001086	7.60E-03
uc002rdc.3	OSR1	6641.1	-2.48	0.64	-3.87	0.000109	7.60E-03

uc004dbh.3	PDK3	6707.1	1.85	0.478	3.867	0.0001102	7.70E-03
uc002ojd.1	CAPN12	30.8	1.73	0.447	3.867	0.0001101	7.70E-03
uc003cps.1	RTP3	4	3.51	0.909	3.866	0.0001108	7.70E-03
uc001gyj.3	RP11-480I12.5	81.2	-2.44	0.632	-3.861	0.000113	7.80E-03
uc003zvn.3	CCL19	40.6	-3.57	0.927	-3.853	0.0001166	8.00E-03
uc031qoh.1	PTCSC3	56.2	3.56	0.924	3.854	0.0001162	8.00E-03
uc001xbt.3	DLGAP5	82	-1.91	0.495	-3.853	0.0001167	8.00E-03
uc003aoe.3	APOL6	3003.9	2.69	0.698	3.855	0.0001159	8.00E-03
uc003nim.2	GUSBP2	80.7	-1.49	0.386	-3.852	0.0001171	8.00E-03
uc002ecy.3	TP53TG3D	56.6	-3.16	0.821	-3.851	0.0001175	8.00E-03
uc002oet.3		800	1.81	0.47	3.851	0.0001178	8.00E-03
uc001kos.3	GOLGA7B	223.4	3.38	0.879	3.849	0.0001187	8.10E-03
uc003yoz.3	COL14A1	2311.7	-2.81	0.731	-3.845	0.0001208	8.20E-03
uc003vqe.3		222.7	1.5	0.391	3.836	0.0001249	8.40E-03
uc009zii.2	LMO3	7.4	2.84	0.74	3.835	0.0001253	8.40E-03
uc004eoh.3	KCNE1L	736.5	-2.9	0.757	-3.834	0.000126	8.50E-03
uc003jtn.1	CWC27	201.1	-1.27	0.333	-3.832	0.0001269	8.50E-03
uc003inv.1	SFRP2	97308.8	2.96	0.772	3.827	0.0001295	8.60E-03
uc003qaq.1		52.5	-2.21	0.577	-3.828	0.0001293	8.60E-03
uc010dno.1	ST8SIA5	4.1	3.33	0.871	3.826	0.0001303	8.70E-03
uc003mey.3	UNC5A	328	-1.92	0.502	-3.824	0.0001311	8.70E-03
uc001dqe.1		56.2	1.42	0.371	3.82	0.0001333	8.80E-03
uc011bis.2	LSAMP	6.3	-3.6	0.942	-3.818	0.0001347	8.90E-03
uc001sos.4		524.4	-2.32	0.607	-3.816	0.0001355	8.90E-03
uc002omw.4	ZNF780A	2802.6	1.78	0.466	3.812	0.0001376	9.00E-03
uc001drv.3	DPYD	450.7	1.77	0.464	3.808	0.0001402	9.20E-03
uc001bpy.3	PHACTR4	32.5	2.07	0.543	3.803	0.0001428	9.30E-03
uc001ikn.2	CELF2-AS2	21.9	3.14	0.825	3.8	0.0001444	9.40E-03
uc002iep.3	PPY	46.1	-3.36	0.884	-3.798	0.0001458	9.50E-03
uc002opw.3	CYP2S1	46.3	2.41	0.634	3.797	0.0001464	9.50E-03
uc002ped.1	AC006262.6	16.5	3.62	0.954	3.797	0.0001466	9.50E-03
uc010fuz.3	SPAG16	54.5	1.97	0.519	3.795	0.0001478	9.50E-03
uc002rsf.1	C2orf91	83.6	2.56	0.675	3.794	0.0001483	9.50E-03
uc001bcy.3	PLA2G5	3.1	3.71	0.978	3.792	0.0001495	9.60E-03
uc031ras.1	LINC00854	87.7	1.55	0.409	3.792	0.0001494	9.60E-03
uc010eaj.1	SIN3B	21.4	1.82	0.481	3.792	0.0001495	9.60E-03
uc001jhw.3	SLC18A3	62.2	2.87	0.758	3.791	0.0001501	9.60E-03
uc001gmw.3		6.8	3.21	0.848	3.787	0.0001522	9.70E-03
uc010svl.1	ANO4	5.7	2.75	0.726	3.786	0.0001531	9.70E-03
uc002ffw.4	CENPN	123.7	-1.93	0.51	-3.786	0.0001532	9.70E-03
uc003anb.3	TIMP3	56234.7	1.6	0.423	3.784	0.0001541	9.70E-03
uc002btk.4	MCTP2	192.3	-3.33	0.881	-3.781	0.0001562	9.80E-03
uc002rjn.3	TRIM54	3.1	3.39	0.898	3.777	0.000159	9.90E-03
uc001lpx.3	C11orf35	113.2	1.55	0.411	3.777	0.0001587	9.90E-03
uc010ebu.1	TMEM59L	8.6	-3.4	0.9	-3.777	0.0001589	9.90E-03
uc009xfb.2	TRIM17	204.9	2.13	0.564	3.776	0.0001595	9.90E-03
