# Supplementary material for: A unique subset of low-risk Wilms tumors is characterized by loss of function of TRIM28 (KAP1), a gene critical in early renal development: A Children’s Oncology Group study
Source: PLoS One. 2018 Dec 13;13(12):e0208936. doi: 10.1371/journal.pone.0208936 (PMC6292605; doi:10.1371/journal.pone.0208936)
Supplement: S3 Table — RNAseq transposable element data from 3 TRIM28-mutant WTs was compared to six randomly selected TRIM28-wildtype WTs using DESeq2 as described in the Methods. The data were filtered to include TEs with adjusted p-value < 0.001. (PDF) [file pone.0208936.s008.pdf]

transcript_id	baseMean	log2 FoldChange	lfcSE	stat	pvalue	padj	Chr	Start	End	Smith-Waterman alignment Score	Strand	family_id	class_id
LTR4_dup17	1235.29	13.05	0.98	13.30	2.2E-40	3.7E-35	chr3	128573367	128573841	2741	-	ERV1	LTR
L1PB1_dup1604	195.83	5.55	0.43	12.94	2.5E-38	2.1E-33	chr2	168744394	168748490	11933	-	L1	LINE
L1PA6_dup325	448.12	11.85	0.92	12.88	5.5E-38	3.1E-33	chr1	173647228	173649789	12116	+	L1	LINE
SVA_D_dup897	369.98	7.48	0.62	12.10	1.1E-33	4.6E-29	chr11	86590500	86591501	6741	+	SVA_D	Other
SVA_D_dup896	238.39	11.02	0.91	12.07	1.5E-33	5.2E-29	chr11	86589268	86590488	5924	+	SVA_D	Other
HERV3-int_dup261	378.73	11.52	0.97	11.90	1.2E-32	3.3E-28	chr19	24000870	24001211	2987	-	ERV1	LTR
HERV3-int_dup40	476.72	10.13	0.85	11.88	1.5E-32	3.7E-28	chr3	128574342	128575237	5881	-	ERV1	LTR
SVA_D_dup22	150.54	10.39	0.92	11.29	1.5E-29	3.3E-25	chr1	24014645	24016257	10236	+	SVA_D	Other
LTR4_dup91	169.16	10.44	0.98	10.69	1.1E-26	2.1E-22	chr19	24000367	24000595	961	-	ERV1	LTR
HERVI-int_dup23	86.61	9.68	0.91	10.63	2.2E-26	3.6E-22	chr3	110688303	110688564	639	-	ERV1	LTR
SVA_B_dup109	162.86	10.36	0.99	10.49	9.7E-26	1.5E-21	chr4	25250433	25251871	10676	-	SVA_B	Other
SVA_D_dup883	200.04	10.54	1.03	10.23	1.4E-24	2.0E-20	chr11	73646655	73648767	6582	-	SVA_D	Other
HERVIP10F-int_dup359	286.38	4.65	0.46	10.14	3.8E-24	5.0E-20	chr16	30113567	30115156	6288	+	ERV1	LTR
PABL_B_dup349	70.32	3.60	0.36	9.96	2.2E-23	2.6E-19	chr19	37285807	37286288	909	-	ERV1	LTR
HERVK14-int_dup223	139.19	10.09	1.02	9.89	4.4E-23	4.9E-19	chr10	5077666	5080498	22865	+	ERVK	LTR
L1ME3A_dup10708	83.30	9.47	0.99	9.56	1.2E-21	1.2E-17	chr11	69898953	69900191	5114	-	L1	LINE
L1PB1_dup1602	63.74	9.16	0.97	9.45	3.5E-21	3.5E-17	chr2	168742664	168744020	9126	-	L1	LINE
SVA_D_dup924	51.50	8.91	0.95	9.36	8.0E-21	7.5E-17	chr12	32715349	32716995	10232	-	SVA_D	Other
L1ME3A_dup10707	87.33	9.49	1.02	9.34	9.6E-21	8.5E-17	chr11	69897354	69898645	5114	-	L1	LINE
HERV3-int_dup43	78.69	9.37	1.00	9.33	1.0E-20	8.8E-17	chr3	128576610	128578522	14138	-	ERV1	LTR
L1ME1_dup6449	83.06	9.42	1.02	9.28	1.7E-20	1.4E-16	chr3	110540956	110542430	6803	+	L1	LINE
MLT2B4_dup580	42.41	8.67	0.94	9.23	2.6E-20	2.0E-16	chr2	168792208	168792767	2178	+	ERVL	LTR
LTR16E2_dup1617	111.70	9.73	1.05	9.22	2.9E-20	2.1E-16	chr11	32559059	32559225	694	+	ERVL	LTR
PABL_B-int_dup116	153.97	10.05	1.10	9.17	4.8E-20	3.4E-16	chr9	138549197	138550188	16765	+	ERV1	LTR
SVA_F_dup60	100.74	9.58	1.06	9.01	2.0E-19	1.3E-15	chr1	150700421	150702477	7048	-	SVA_F	Other
MER4-int_dup314	98.04	5.04	0.56	8.98	2.8E-19	1.8E-15	chr2	113617978	113620655	16931	+	ERV1	LTR
MIRb_dup28865	46.13	8.72	0.98	8.90	5.6E-19	3.5E-15	chr2	74785372	74785442	224	+	MIR	SINE
HERV3-int_dup262	87.93	9.40	1.06	8.85	8.9E-19	5.4E-15	chr19	24001281	24001567	2987	-	ERV1	LTR
SVA_D_dup393	37.79	8.49	0.96	8.84	9.6E-19	5.6E-15	chr4	186160924	186162680	9088	-	SVA_D	Other
L1MA6_dup2408	38.11	8.50	0.96	8.84	1.0E-18	5.6E-15	chr7	63631869	63633408	9693	-	L1	LINE
LTR16A_dup867	39.08	8.52	0.97	8.81	1.2E-18	6.6E-15	chr2	168671550	168671732	613	+	ERVL	LTR
SVA_D_dup1425	47.52	8.71	1.00	8.68	4.0E-18	2.1E-14	chr1	101119	102591	10642	-	SVA_D	Other
AluSz_dup4689	58.61	8.93	1.04	8.61	7.6E-18	3.8E-14	chr1	116912137	116912440	2162	+	Alu	SINE
SVA_D_dup187	53.50	8.83	1.03	8.61	7.4E-18	3.8E-14	chr2	85714148	85716186	7831	+	SVA_D	Other
L1MA2_dup7198	32.88	8.28	0.97	8.50	1.9E-17	9.0E-14	chr19	23155601	23155798	759	+	L1	LINE
HERVI-int_dup132	570.14	7.37	0.87	8.45	2.9E-17	1.4E-13	chr18	44450608	44456660	30365	+	ERV1	LTR
AluSc8_dup2866	31.82	8.23	0.98	8.43	3.5E-17	1.6E-13	chr2	168670990	168671282	2198	+	Alu	SINE

Tigger1_dup557	59.08	8.89	1.06	8.38	5.3E-17	2.4E-13	chr1	150695711	150697591	14235	-	TcMar-Tigger	DNA
L2a_dup35574	64.39	8.97	1.07	8.35	6.7E-17	2.9E-13	chr3	110568586	110569363	1238	-	L2	LINE
L1PA5_dup6340	53.93	8.79	1.05	8.35	6.9E-17	2.9E-13	chr9	95755749	95761870	24425	-	L1	LINE
HERVK3-int_dup248	34.37	8.30	1.00	8.34	7.4E-17	3.1E-13	chr19	7845104	7846642	6385	+	ERVK	LTR
MER4-int_dup168	60.82	8.90	1.07	8.30	1.0E-16	4.1E-13	chr1	173644772	173645942	15271	-	ERV1	LTR
L1MEc_dup13105	31.35	8.19	0.99	8.27	1.4E-16	5.3E-13	chr11	69888236	69890513	5224	+	L1	LINE
L2b_dup89327	31.19	8.17	1.00	8.17	3.0E-16	1.1E-12	chr19	7848574	7848792	297	+	L2	LINE
L1M2_dup1638	50.11	8.66	1.07	8.10	5.6E-16	2.1E-12	chr3	110557024	110557716	3662	+	L1	LINE
L1MA7_dup8069	27.50	8.01	0.99	8.05	8.2E-16	3.0E-12	chr19	22146668	22146936	1057	+	L1	LINE
L1PA5_dup8770	172.85	5.61	0.70	8.02	1.0E-15	3.7E-12	chr12	57363005	57367556	25123	+	L1	LINE
L1ME1_dup6452	38.54	8.36	1.05	7.99	1.4E-15	4.9E-12	chr3	110590148	110591118	2827	-	L1	LINE
Harlequin-int_dup71	121.94	3.98	0.50	7.97	1.5E-15	5.3E-12	chr1	206282942	206286485	24781	-	ERV1	LTR
L1PA2_dup912	60.32	3.80	0.48	7.97	1.6E-15	5.3E-12	chr3	125655482	125661512	26678	+	L1	LINE
ERVL-B4-int_dup2340	34.50	8.23	1.04	7.95	1.9E-15	6.2E-12	chrX	130794952	130795768	1785	+	ERVL	LTR
L1PA3_dup1574	204.91	6.12	0.77	7.93	2.3E-15	7.3E-12	chr3	11530147	11533964	15774	-	L1	LINE
L1PA17_dup4562	33.43	8.19	1.04	7.90	2.8E-15	8.9E-12	chr19	23148237	23149532	6157	-	L1	LINE
Charlie25_dup999	26.49	7.94	1.01	7.88	3.2E-15	1.0E-11	chr19	22147384	22147575	279	+	hAT-Charlie	DNA
SVA_D_dup720	61.60	8.81	1.12	7.84	4.5E-15	1.4E-11	chr9	131541764	131543445	9638	+	SVA_D	Other
AluSx1_dup22178	27.42	7.97	1.02	7.82	5.2E-15	1.6E-11	chr3	194217781	194218078	2146	+	Alu	SINE
SVA_D_dup584	32.61	8.15	1.04	7.80	6.3E-15	1.9E-11	chr7	73210949	73212455	11057	-	SVA_D	Other
SVA_F_dup201	63.56	8.83	1.13	7.78	7.1E-15	2.1E-11	chr3	61419455	61421381	7023	-	SVA_F	Other
L1PA3_dup1088	36.30	8.25	1.06	7.75	9.3E-15	2.7E-11	chr2	105822259	105825907	24052	-	L1	LINE
L1MA5A_dup738	47.27	8.52	1.10	7.74	1.0E-14	2.8E-11	chr3	110536304	110537357	3886	-	L1	LINE
LTR13_dup405	26.50	7.91	1.02	7.74	1.0E-14	2.8E-11	chr19	22063054	22064074	7750	-	ERVK	LTR
L1PA3_dup8496	33.81	8.17	1.06	7.73	1.1E-14	2.9E-11	chr12	59619067	59622257	26392	-	L1	LINE
L1MB3_dup7726	104.44	-5.39	0.70	-7.72	1.2E-14	3.2E-11	chr8	60882514	60882963	2328	-	L1	LINE
L1MC1_dup3529	48.44	8.53	1.11	7.69	1.4E-14	3.8E-11	chr4	78926746	78927638	5369	+	L1	LINE
MER58A_dup3024	24.39	7.81	1.02	7.66	1.9E-14	4.8E-11	chr3	194217344	194217536	1110	+	hAT-Charlie	DNA
HERVI-int_dup97	35.75	8.21	1.07	7.66	1.9E-14	4.9E-11	chr12	10055801	10059965	30882	-	ERV1	LTR
L1PA2_dup1730	29.62	8.01	1.05	7.63	2.3E-14	5.9E-11	chr5	136118489	136124518	26512	-	L1	LINE
L1PA3_dup8495	34.90	8.18	1.07	7.61	2.7E-14	6.7E-11	chr12	59615794	59618760	26392	-	L1	LINE
SVA_A_dup114	112.45	-3.52	0.46	-7.61	2.7E-14	6.7E-11	chr8	70767849	70768792	6710	+	SVA_A	Other
AluSx1_dup6260	74.90	8.94	1.18	7.60	3.0E-14	7.2E-11	chr1	173676472	173676769	2215	-	Alu	SINE
THE1A_dup595	24.56	7.80	1.03	7.55	4.3E-14	1.0E-10	chr2	168797543	168797879	2379	-	ERVL-MaLR	LTR
AluJb_dup9830	69.90	8.86	1.17	7.55	4.5E-14	1.1E-10	chr1	173674521	173674828	1806	+	Alu	SINE
L1HS_dup290	804.42	5.98	0.79	7.53	4.9E-14	1.1E-10	chr3	110607662	110609395	14431	+	L1	LINE
L2_dup46253	31.16	8.04	1.07	7.53	5.0E-14	1.1E-10	chr14	56410378	56410619	777	-	L2	LINE
MLT2B2_dup483	27.31	7.90	1.05	7.52	5.5E-14	1.2E-10	chr3	110582629	110583105	2355	-	ERVL	LTR
Tigger1_dup813	31.89	8.07	1.07	7.52	5.4E-14	1.2E-10	chr1	213978232	213979393	8178	-	TcMar-Tigger	DNA
L1ME3C_dup591	42.94	8.36	1.12	7.49	7.0E-14	1.5E-10	chr1	173618142	173619060	1571	-	L1	LINE
LTR25-int_dup551	74.83	7.35	0.98	7.47	7.9E-14	1.7E-10	chr19	22129433	22133290	19794	+	ERV1	LTR
MER4-int_dup169	30.95	8.02	1.07	7.47	8.0E-14	1.7E-10	chr1	173645993	173647049	15271	-	ERV1	LTR

SVA_F_dup820	31.13	8.03	1.08	7.46	9.0E-14	1.9E-10	chr16	2851338	2852861	10254	-	SVA_F	Other
L1PA3_dup3271	52.22	7.05	0.95	7.44	9.9E-14	2.1E-10	chr5	38169599	38175631	28173	-	L1	LINE
L1MB7_dup5692	48.32	8.46	1.14	7.41	1.2E-13	2.5E-10	chr4	78925444	78926188	4040	-	L1	LINE
MER5B_dup4876	24.83	7.78	1.05	7.41	1.2E-13	2.5E-10	chr3	110558779	110558928	401	+	hAT-Charlie	DNA
L1P2_dup1478	25.14	7.79	1.05	7.40	1.3E-13	2.7E-10	chr19	22136559	22139767	16575	-	L1	LINE
L1M5_dup59435	23.41	7.72	1.04	7.40	1.4E-13	2.7E-10	chr19	7849682	7850125	785	-	L1	LINE
L1ME1_dup4706	193.87	-9.01	1.22	-7.38	1.5E-13	3.0E-10	chr2	181784643	181785934	1958	+	L1	LINE
HERV3-int_dup284	61.53	8.69	1.18	7.37	1.7E-13	3.4E-10	chr21	39596486	39597405	6747	-	ERV1	LTR
BSR/Beta_dup1580	23.96	7.73	1.05	7.35	2.0E-13	3.9E-10	chr19	22073355	22075704	1594	-	Satellite	Satellite
SVA_F_dup908	26.98	7.86	1.07	7.34	2.1E-13	4.0E-10	chr19	22047545	22048793	7175	-	SVA_F	Other
AluJr_dup8246	22.37	7.66	1.04	7.34	2.1E-13	4.0E-10	chr2	74785024	74785162	874	-	Alu	SINE
L1MA4A_dup842	553.46	-6.49	0.89	-7.33	2.3E-13	4.3E-10	chr2	181747207	181753308	16596	+	L1	LINE
L2_dup11390	24.03	7.72	1.06	7.28	3.4E-13	6.3E-10	chr3	110542704	110543224	423	+	L2	LINE
AluSx_dup7709	33.50	8.06	1.11	7.27	3.5E-13	6.4E-10	chr1	150697807	150698092	2277	-	Alu	SINE
PABL_B-int_dup117	418.02	10.35	1.43	7.26	3.9E-13	7.1E-10	chr9	138550493	138552410	16765	+	ERV1	LTR
HERVK13-int_dup34	49.40	8.44	1.16	7.26	4.0E-13	7.1E-10	chr10	5080755	5088879	74907	-	ERVK	LTR
MER34_dup158	55.93	4.82	0.66	7.25	4.1E-13	7.1E-10	chr2	168816510	168817038	2306	+	ERV1	LTR
L1PA3_dup195	102.22	4.31	0.60	7.22	5.2E-13	9.1E-10	chr1	76143436	76149470	26056	+	L1	LINE
MIRc_dup22689	22.71	7.65	1.06	7.22	5.4E-13	9.3E-10	chr3	110604260	110604449	438	-	MIR	SINE
AluSx1_dup99752	20.32	7.52	1.05	7.17	7.4E-13	1.3E-09	chr19	23156186	23156493	2046	+	Alu	SINE
LTR57_dup24	21.34	7.57	1.06	7.16	7.9E-13	1.3E-09	chr2	168790346	168790746	2663	+	ERVL	LTR
LTR10B1_dup181	123.39	4.24	0.59	7.16	8.3E-13	1.4E-09	chr16	30115179	30115488	1209	+	ERV1	LTR
L1PA7_dup2456	20.36	7.52	1.05	7.15	8.9E-13	1.5E-09	chr3	110561757	110562463	4996	-	L1	LINE
MIR3_dup19545	25.12	7.74	1.08	7.14	9.5E-13	1.6E-09	chr3	110606245	110606348	388	+	MIR	SINE
SVA_D_dup1315	34.75	8.06	1.13	7.12	1.1E-12	1.8E-09	chr20	31702735	31704267	11083	+	SVA_D	Other
LTR10C_dup35	50.36	8.42	1.18	7.11	1.1E-12	1.8E-09	chr1	173611593	173612174	3252	+	ERV1	LTR
L2c_dup114624	27.74	7.83	1.10	7.11	1.2E-12	1.9E-09	chr14	56428663	56428765	247	-	L2	LINE
AluSq2_dup49967	25.98	7.77	1.09	7.10	1.2E-12	1.9E-09	chr19	23154851	23155145	2339	+	Alu	SINE
L2a_dup120922	19.99	7.48	1.06	7.03	2.1E-12	3.3E-09	chr12	32707396	32708485	1972	-	L2	LINE
MER63A_dup461	21.43	7.55	1.08	7.01	2.3E-12	3.6E-09	chr2	168671365	168671548	450	-	ıAT-Blackjacl	DNA
HAL1_dup1847	53.38	8.45	1.21	7.00	2.5E-12	3.8E-09	chr1	221979707	221980454	849	-	L1	LINE
MER45C_dup1029	25.50	7.72	1.10	6.99	2.7E-12	4.1E-09	chr17	5447201	5448103	2926	+	hAT-Tip100	DNA
SVA_C_dup18	22.85	7.61	1.09	6.99	2.8E-12	4.3E-09	chr1	153546936	153548457	10297	+	SVA_C	Other
L1PA3_dup1732	39.76	3.25	0.47	6.98	2.9E-12	4.4E-09	chr3	51236480	51242602	27553	+	L1	LINE
AluSx1_dup5341	25.16	7.69	1.11	6.93	4.2E-12	6.3E-09	chr1	150698385	150698690	2298	-	Alu	SINE
L2a_dup11421	39.14	8.13	1.17	6.92	4.4E-12	6.5E-09	chr1	173630836	173631167	439	-	L2	LINE
SVA_D_dup1314	24.08	7.65	1.11	6.92	4.6E-12	6.7E-09	chr20	30709007	30711015	6931	-	SVA_D	Other
L1PA5_dup4999	61.81	6.82	0.99	6.89	5.6E-12	8.0E-09	chr7	63620912	63627011	26289	+	L1	LINE
MIRc_dup96239	27.43	7.77	1.13	6.89	5.7E-12	8.2E-09	chr19	7831145	7831209	277	+	MIR	SINE
PABL_B-int_dup121	54.45	8.43	1.22	6.88	5.9E-12	8.4E-09	chr9	138552896	138553426	5289	+	ERV1	LTR
L1PB1_dup1603	20.69	7.48	1.09	6.88	6.1E-12	8.6E-09	chr2	168744080	168744393	9126	-	L1	LINE
L1MA3_dup1112	122.34	-8.45	1.23	-6.88	6.2E-12	8.6E-09	chr2	181738282	181740547	18337	+	L1	LINE

L1PA4_dup1876	23.71	7.61	1.11	6.84	8.2E-12	1.1E-08	chr3	30470142	30475120	25852	+	L1	LINE
MER115_dup1804	23.15	7.58	1.11	6.83	8.5E-12	1.2E-08	chr11	69900507	69900747	950	+	hAT-Tip100	DNA
L1MDa_dup1003	116.13	-8.38	1.23	-6.81	9.7E-12	1.3E-08	chr2	181783809	181784657	2428	+	L1	LINE
L1PA3_dup6893	22.16	7.53	1.11	6.80	1.0E-11	1.4E-08	chrX	121011210	121017373	27253	+	L1	LINE
AluSx1_dup44180	313.02	-2.90	0.43	-6.79	1.1E-11	1.5E-08	chr7	148116512	148116835	2125	-	Alu	SINE
MER4-int_dup173	38.67	8.08	1.19	6.78	1.2E-11	1.6E-08	chr1	173651204	173651823	5100	-	ERV1	LTR
LTR8A_dup141	36.83	8.01	1.19	6.73	1.7E-11	2.3E-08	chr1	173682385	173683127	3621	+	ERV1	LTR
L1MB4_dup8471	29.26	7.79	1.16	6.72	1.8E-11	2.4E-08	chr19	7885171	7886438	3345	+	L1	LINE
Charlie1_dup92	38.87	8.06	1.20	6.71	1.9E-11	2.5E-08	chr1	173636871	173637332	2834	+	hAT-Charlie	DNA
AluSc_dup30991	25.12	7.63	1.14	6.70	2.1E-11	2.7E-08	chr19	7833180	7833491	2412	+	Alu	SINE
L1PA3_dup10035	19.90	7.40	1.11	6.69	2.2E-11	2.8E-08	chr18	33340273	33346376	27820	+	L1	LINE
L1ME1_dup6443	42.05	8.13	1.22	6.68	2.3E-11	3.0E-08	chr3	110534520	110535127	1347	+	L1	LINE
L1M4b_dup2317	24.68	7.61	1.14	6.67	2.5E-11	3.2E-08	chr6	122022260	122024068	6745	+	L1	LINE
L1PA3_dup6480	21.80	7.49	1.12	6.67	2.5E-11	3.2E-08	chrX	69003131	69009162	26264	-	L1	LINE
L1MDa_dup5835	20.37	7.42	1.11	6.66	2.7E-11	3.3E-08	chr15	27429735	27431166	4560	-	L1	LINE
MSTB_dup6459	16.83	7.22	1.08	6.66	2.8E-11	3.4E-08	chr13	31412471	31412657	863	-	ERVL-MaLR	LTR
AluJr_dup3749	25.34	7.63	1.15	6.65	2.9E-11	3.5E-08	chr1	150699869	150700195	1549	-	Alu	SINE
LTR12C_dup320	106.99	-8.25	1.24	-6.64	3.1E-11	3.8E-08	chr2	181555932	181557152	7781	+	ERV1	LTR
HERV16-int_dup80	328.10	9.79	1.47	6.64	3.1E-11	3.8E-08	chr1	173673117	173673906	1508	-	ERVL	LTR
L1PA17_dup4563	19.63	7.37	1.11	6.64	3.2E-11	3.9E-08	chr19	23149762	23151104	6157	-	L1	LINE
HERVK9-int_dup580	40.57	8.07	1.22	6.63	3.4E-11	4.0E-08	chr17	47248517	47253015	36910	-	ERVK	LTR
MER20B_dup3131	23.05	7.53	1.14	6.63	3.4E-11	4.0E-08	chr13	52702040	52702641	1553	+	hAT-Charlie	DNA
SVA_B_dup378	28.33	7.73	1.17	6.63	3.4E-11	4.0E-08	chr14	81928235	81929483	10082	-	SVA_B	Other
AluY_dup6941	40.49	8.07	1.22	6.62	3.6E-11	4.1E-08	chr1	173672347	173672641	2386	-	Alu	SINE
L1ME2_dup1943	108.83	-8.25	1.25	-6.62	3.6E-11	4.2E-08	chr2	181745935	181747207	3299	+	L1	LINE
L1PA3_dup10282	20.50	7.41	1.12	6.61	3.9E-11	4.4E-08	chr20	4264368	4270528	27500	-	L1	LINE
L1PA4_dup2325	84.85	4.84	0.73	6.60	4.0E-11	4.6E-08	chr3	122451719	122457897	25757	+	L1	LINE
LTR70_dup107	17.76	7.26	1.10	6.59	4.3E-11	4.8E-08	chr19	22178942	22180219	8900	-	ERV1	LTR
LTR21B_dup57	41.39	8.08	1.23	6.58	4.7E-11	5.2E-08	chr19	22798317	22798705	902	-	ERV1	LTR
LTR25_dup257	19.64	7.36	1.12	6.58	4.7E-11	5.2E-08	chr19	22128598	22129432	5098	+	ERV1	LTR
HERV17-int_dup4	17.09	7.22	1.10	6.58	4.7E-11	5.2E-08	chr1	46852091	46854826	33173	-	ERV1	LTR
L1MEc_dup5682	201.93	-2.37	0.36	-6.58	4.8E-11	5.3E-08	chr5	184398	184712	883	+	L1	LINE
HERVIP10F-int_dup153	293.94	9.69	1.47	6.57	5.0E-11	5.4E-08	chr6	118897432	118897771	792	+	ERV1	LTR
MIRb_dup142590	86.47	-8.05	1.23	-6.57	5.2E-11	5.7E-08	chr10	130522236	130522432	270	+	MIR	SINE
L2c_dup11139	23.73	7.54	1.15	6.56	5.2E-11	5.7E-08	chr1	173638176	173638498	534	-	L2	LINE
HERVIP10F-int_dup278	23.65	7.54	1.15	6.56	5.5E-11	5.9E-08	chr11	69884674	69884961	573	+	ERV1	LTR
LTR43_dup329	17.98	7.26	1.11	6.56	5.5E-11	5.9E-08	chr19	22127625	22128230	3326	+	ERV1	LTR
AluSx3_dup26679	17.91	7.26	1.11	6.55	5.6E-11	5.9E-08	chr19	22125440	22125750	2071	-	Alu	SINE
AluSc_dup6503	40.04	8.04	1.23	6.55	5.6E-11	5.9E-08	chr3	110682909	110683223	2406	-	Alu	SINE
L2b_dup15038	18.71	7.30	1.12	6.54	6.1E-11	6.4E-08	chr2	168789724	168789855	432	-	L2	LINE
MIR_dup119635	30.71	7.79	1.19	6.54	6.2E-11	6.4E-08	chr11	114648434	114648593	426	+	MIR	SINE
SVA_D_dup185	18.73	7.30	1.12	6.54	6.2E-11	6.4E-08	chr2	75022244	75024207	8001	-	SVA_D	Other

HERV3-int_dup39	888.90	9.17	1.40	6.54	6.2E-11	6.4E-08	chr3	128573892	128574321	2733	-	ERV1	LTR
SVA_D_dup1363	17.01	7.20	1.10	6.54	6.4E-11	6.5E-08	chr22	30253578	30255007	5968	-	SVA_D	Other
L2a_dup129198	16.16	7.15	1.10	6.52	7.2E-11	7.3E-08	chr13	31410735	31410858	213	+	L2	LINE
MER4A1_dup241	81.98	4.57	0.70	6.50	8.0E-11	8.1E-08	chr2	113620664	113621118	3215	+	ERV1	LTR
THE1B-int_dup842	30.77	7.77	1.20	6.49	8.6E-11	8.7E-08	chr3	110422432	110423957	9143	-	ERVL-MaLR	LTR
L1MA3_dup1113	95.39	-8.09	1.25	-6.47	9.6E-11	9.6E-08	chr2	181740904	181743924	18337	+	L1	LINE
MER102c_dup3418	40.91	8.03	1.24	6.46	1.0E-10	1.0E-07	chr21	34163224	34163396	448	+	hAT-Charlie	DNA
L2b_dup61231	606.89	-5.98	0.93	-6.46	1.1E-10	1.1E-07	chr11	4676142	4676354	241	+	L2	LINE
LTR5_Hs_dup89	26.39	7.62	1.18	6.45	1.1E-10	1.1E-07	chr2	113691831	113692798	7836	+	ERVK	LTR
AluSx1_dup14078	16.03	7.12	1.10	6.45	1.1E-10	1.1E-07	chr2	168791625	168791897	1831	+	Alu	SINE
LTR71A_dup8	30.06	7.74	1.20	6.44	1.2E-10	1.2E-07	chr1	173639984	173640472	2102	-	ERV1	LTR
L1PA4_dup100	24.90	7.55	1.17	6.43	1.3E-10	1.2E-07	chr1	42524947	42531043	23756	+	L1	LINE
MLT1C_dup2253	15.88	7.10	1.11	6.41	1.4E-10	1.4E-07	chr2	168791257	168791550	2267	+	ERVL-MaLR	LTR
LTR10B1_dup80	453.18	9.70	1.51	6.40	1.5E-10	1.4E-07	chr6	118899243	118899612	1627	+	ERV1	LTR
L1PA4_dup1480	18.88	7.28	1.14	6.40	1.6E-10	1.5E-07	chr2	168660837	168666936	24935	-	L1	LINE
MIRb_dup48442	20.57	7.36	1.15	6.39	1.7E-10	1.6E-07	chr3	110606064	110606147	269	+	MIR	SINE
L2c_dup11138	24.98	7.54	1.18	6.38	1.8E-10	1.7E-07	chr1	173633600	173633933	193	+	L2	LINE
MER75_dup294	29.34	7.69	1.21	6.36	2.0E-10	1.8E-07	chr10	5119903	5120334	2859	-	PiggyBac	DNA
L1MC1_dup202	36.96	7.90	1.24	6.36	2.1E-10	1.9E-07	chr1	58395680	58398074	14101	-	L1	LINE
MIRb_dup149668	21.23	7.37	1.17	6.32	2.6E-10	2.4E-07	chr11	69896837	69897005	610	+	MIR	SINE
L1PA3_dup1582	29.67	7.69	1.22	6.32	2.6E-10	2.4E-07	chr3	15228173	15234206	26453	+	L1	LINE
HERVIP10FH-int_dup20	14.81	7.01	1.11	6.30	3.0E-10	2.7E-07	chr1	46850306	46851452	7777	-	ERV1	LTR
AluSx_dup7953	32.21	7.76	1.23	6.30	3.0E-10	2.7E-07	chr1	153561360	153561678	2103	+	Alu	SINE
MIR_dup41131	14.73	7.00	1.12	6.27	3.6E-10	3.2E-07	chr3	194217144	194217343	737	-	MIR	SINE
L1ME4a_dup4621	33.06	-7.09	1.13	-6.27	3.6E-10	3.3E-07	chr2	70906939	70907951	758	-	L1	LINE
Charlie1_dup91	26.86	7.58	1.21	6.26	3.8E-10	3.4E-07	chr1	173636188	173636560	2834	+	hAT-Charlie	DNA
L1ME3C_dup590	23.46	7.45	1.19	6.26	3.8E-10	3.4E-07	chr1	173615355	173615552	746	-	L1	LINE
L1ME3_dup3329	24.88	7.50	1.20	6.26	3.9E-10	3.5E-07	chr6	45984332	45984890	1342	-	L1	LINE
LTR16C_dup5576	23.15	7.43	1.19	6.22	4.9E-10	4.3E-07	chr19	14456286	14456545	788	+	ERVL	LTR
AluSx3_dup26698	14.35	6.96	1.12	6.22	4.9E-10	4.3E-07	chr19	23156761	23157053	1801	-	Alu	SINE
MER61-int_dup902	22.93	7.41	1.19	6.22	5.0E-10	4.4E-07	chr12	10051928	10054829	20237	-	ERV1	LTR
HERVIP10F-int_dup143	35.80	7.83	1.26	6.21	5.2E-10	4.5E-07	chr6	35532548	35533480	3118	-	ERV1	LTR
L1PB2_dup2051	14.74	6.98	1.13	6.19	6.0E-10	5.2E-07	chr11	45134363	45135339	8349	-	L1	LINE
LTR16A_dup326	32.50	7.73	1.25	6.19	6.2E-10	5.3E-07	chr1	173676857	173676986	543	+	ERVL	LTR
MIRb_dup48440	16.94	7.11	1.15	6.16	7.3E-10	6.2E-07	chr3	110559175	110559312	457	+	MIR	SINE
L1PA3_dup7397	15.25	7.00	1.14	6.14	8.1E-10	6.9E-07	chr10	22692067	22698081	27432	-	L1	LINE
LTR16A_dup870	23.38	7.41	1.21	6.13	8.6E-10	7.3E-07	chr2	168832400	168832613	516	+	ERVL	LTR
MIRc_dup46595	142.02	-2.81	0.46	-6.13	8.7E-10	7.3E-07	chr7	148116932	148117016	233	+	MIR	SINE
MIRb_dup150056	20.06	7.26	1.19	6.12	9.1E-10	7.6E-07	chr11	73646052	73646164	250	-	MIR	SINE
AluSg_dup34793	973.79	-2.03	0.33	-6.12	9.4E-10	7.8E-07	chr17	42638099	42638381	2055	-	Alu	SINE
AluJr_dup3748	17.82	7.14	1.17	6.11	9.7E-10	8.0E-07	chr1	150698095	150698382	1633	-	Alu	SINE
MIR_dup11981	23.07	7.39	1.21	6.11	1.0E-09	8.4E-07	chr1	173614554	173614767	787	-	MIR	SINE

SST1_dup585	85.71	-5.64	0.93	-6.10	1.1E-09	8.7E-07	chr20	29517998	29519905	2506	+	centr	Satellite
MIRb_dup17441	21.35	7.31	1.20	6.09	1.1E-09	9.3E-07	chr1	173634003	173634199	376	+	MIR	SINE
L2b_dup7596	21.85	7.33	1.20	6.08	1.2E-09	9.5E-07	chr1	173614869	173614972	181	+	L2	LINE
L2c_dup23892	136.47	-6.13	1.01	-6.08	1.2E-09	9.5E-07	chr2	181709115	181710896	588	-	L2	LINE
L1PA2_dup3705	15.10	6.97	1.15	6.07	1.3E-09	1.0E-06	chr11	75459662	75465694	26749	+	L1	LINE
L1PA7_dup3902	24.19	7.42	1.22	6.06	1.3E-09	1.1E-06	chr5	180353024	180359355	22321	+	L1	LINE
L2a_dup146216	23.33	7.38	1.22	6.06	1.4E-09	1.1E-06	chr16	56767899	56768764	1198	-	L2	LINE
L1PA3_dup7773	14.28	6.91	1.14	6.05	1.4E-09	1.1E-06	chr11	6164888	6170684	26041	+	L1	LINE
L1MC4a_dup1274	136.67	8.93	1.48	6.04	1.5E-09	1.2E-06	chr1	173675778	173676310	1568	-	L1	LINE
PABL_B-int_dup122	29.30	7.58	1.26	6.04	1.5E-09	1.2E-06	chr9	138553717	138554136	5289	+	ERV1	LTR
L1PA16_dup12206	16.41	7.04	1.17	6.03	1.7E-09	1.3E-06	chr15	27431168	27432863	4865	-	L1	LINE
AluSx1_dup47837	13.49	6.85	1.14	6.02	1.7E-09	1.3E-06	chr8	95140055	95140330	2023	+	Alu	SINE
L2b_dup15435	58.19	-7.52	1.25	-6.02	1.7E-09	1.3E-06	chr2	181691922	181692452	267	-	L2	LINE
L1PA3_dup2018	18.39	7.15	1.19	6.02	1.8E-09	1.4E-06	chr3	120299602	120303501	26437	+	L1	LINE
L1ME1_dup3505	13.65	6.86	1.14	6.02	1.8E-09	1.4E-06	chr2	72298922	72299998	2554	-	L1	LINE
L1PA2_dup37	124.83	4.16	0.69	6.01	1.8E-09	1.4E-06	chr1	58592128	58598157	28345	-	L1	LINE
HERVI-int_dup35	51.43	4.71	0.78	6.00	2.0E-09	1.5E-06	chr4	99935123	99942209	19371	-	ERV1	LTR
L1MC4a_dup1272	29.67	7.58	1.26	6.00	2.0E-09	1.5E-06	chr1	173674905	173675161	1568	-	L1	LINE
AluJr4_dup11102	22.76	7.34	1.23	5.99	2.1E-09	1.6E-06	chr11	32511083	32511393	1479	-	Alu	SINE
L1ME3A_dup10706	16.80	7.05	1.18	5.98	2.3E-09	1.7E-06	chr11	69897130	69897339	691	-	L1	LINE
MLT1A1_dup5563	20.29	7.22	1.21	5.96	2.5E-09	1.9E-06	chr14	56429338	56429734	2120	+	ERVL-MaLR	LTR
AluSx1_dup67018	18.48	7.13	1.20	5.96	2.6E-09	1.9E-06	chr11	69895793	69896123	2075	-	Alu	SINE
L1ME4a_dup3009	26.79	7.47	1.26	5.95	2.8E-09	2.0E-06	chr1	221979069	221979467	819	+	L1	LINE
AluSq_dup19547	23.46	7.35	1.24	5.94	2.8E-09	2.1E-06	chr19	15343406	15343707	2520	+	Alu	SINE
SVA_D_dup427	24.25	7.38	1.24	5.93	2.9E-09	2.2E-06	chr5	68810357	68812368	7785	-	SVA_D	Other
HERVIP10F-int_dup145	25.60	7.42	1.25	5.92	3.2E-09	2.3E-06	chr6	35534186	35534606	1264	-	ERV1	LTR
THE1A-int_dup1395	24.09	7.37	1.24	5.92	3.2E-09	2.4E-06	chr20	31709608	31711182	11531	+	ERVL-MaLR	LTR
LTR75_1_dup113	101.10	3.44	0.58	5.91	3.3E-09	2.4E-06	chr19	17529638	17530194	2596	+	ERV1	LTR
AluSc_dup6501	198.05	4.65	0.79	5.91	3.4E-09	2.4E-06	chr3	110610390	110610697	2518	+	Alu	SINE
L1PB4_dup824	69.19	-7.59	1.29	-5.91	3.5E-09	2.5E-06	chr2	181352824	181356776	13447	-	L1	LINE
L1ME1_dup6451	16.78	7.02	1.19	5.90	3.6E-09	2.5E-06	chr3	110555276	110556030	2531	-	L1	LINE
L1ME3B_dup5374	25.84	7.42	1.26	5.90	3.6E-09	2.6E-06	chr11	32505836	32506264	927	+	L1	LINE
AluSx1_dup4833	21.34	7.25	1.23	5.90	3.6E-09	2.6E-06	chr1	113605162	113605459	1886	-	Alu	SINE
MIR3_dup73727	25.85	7.42	1.26	5.90	3.7E-09	2.6E-06	chr15	52580322	52580468	246	-	MIR	SINE
THE1B-int_dup1710	13.33	6.80	1.16	5.87	4.3E-09	3.0E-06	chr5	170517021	170518591	11686	+	ERVL-MaLR	LTR
L1PA3_dup7599	15.99	6.97	1.19	5.87	4.3E-09	3.0E-06	chr10	86532817	86539018	24634	-	L1	LINE
MIRb_dup208479	18.10	7.09	1.21	5.87	4.5E-09	3.1E-06	chr19	14455648	14455787	262	+	MIR	SINE
SVA_D_dup985	207.78	5.40	0.92	5.86	4.5E-09	3.2E-06	chr13	31412722	31414778	7329	-	SVA_D	Other
SVA_D_dup25	13.15	6.78	1.16	5.85	4.8E-09	3.3E-06	chr1	24357916	24359387	10684	-	SVA_D	Other
L2b_dup62459	26.46	7.43	1.27	5.85	4.9E-09	3.4E-06	chr11	32506748	32506838	222	-	L2	LINE
L1MC4a_dup18147	18.20	7.08	1.21	5.85	5.0E-09	3.4E-06	chr11	32536989	32537481	2695	+	L1	LINE
HERVH-int_dup1257	15.62	6.94	1.19	5.84	5.4E-09	3.7E-06	chr3	128682572	128683131	3678	+	ERV1	LTR

L1PA16_dup8936	19.49	7.14	1.23	5.83	5.6E-09	3.8E-06	chrX	120958196	120960643	12419	+	L1	LINE
L1ME1_dup6441	29.29	7.51	1.29	5.82	5.9E-09	4.0E-06	chr3	110319861	110321712	4716	+	L1	LINE
L1PBa_dup1943	13.08	6.76	1.16	5.82	5.9E-09	4.0E-06	chr15	55214432	55216167	10418	-	L1	LINE
L1P2_dup217	70.39	-5.57	0.96	-5.82	6.0E-09	4.0E-06	chr2	181629829	181634049	17738	+	L1	LINE
HERVIP10F-int_dup182	27.80	7.46	1.28	5.81	6.2E-09	4.1E-06	chr7	121146199	121147863	6518	-	ERV1	LTR
MLT1J1_dup3308	410.53	-5.57	0.96	-5.81	6.3E-09	4.2E-06	chr11	4676360	4676726	634	+	ERVL-MaLR	LTR
MLT2D_dup3222	22.33	7.26	1.25	5.81	6.3E-09	4.2E-06	chr11	32504449	32504781	1610	-	ERVL	LTR
MER11C_dup362	13.06	6.76	1.16	5.80	6.5E-09	4.3E-06	chr7	63596940	63598016	7468	+	ERVK	LTR
MIRb_dup139455	395.56	5.20	0.90	5.80	6.6E-09	4.4E-06	chr10	99624793	99625031	738	-	MIR	SINE
L1PA3_dup3196	20.51	7.18	1.24	5.80	6.7E-09	4.4E-06	chr5	15292040	15298198	27191	+	L1	LINE
L1PBa_dup2133	13.30	6.77	1.17	5.80	6.7E-09	4.4E-06	chr20	18222673	18224296	20584	+	L1	LINE
L1PA3_dup2541	23.01	7.28	1.26	5.79	6.9E-09	4.5E-06	chr4	55395102	55401260	27668	-	L1	LINE
L1MC5_dup20215	12.42	6.70	1.16	5.79	6.9E-09	4.5E-06	chr22	32945887	32946164	343	+	L1	LINE
L2_dup43574	12.60	6.72	1.16	5.79	7.1E-09	4.6E-06	chr13	31411645	31411809	186	-	L2	LINE
MER74A_dup1417	17.69	7.04	1.22	5.79	7.2E-09	4.7E-06	chr19	7832742	7833179	1600	-	ERVL	LTR
L1PA6_dup3718	20.84	7.19	1.24	5.78	7.4E-09	4.7E-06	chrX	82043283	82049463	21866	+	L1	LINE
MIRb_dup149667	17.03	7.00	1.21	5.78	7.3E-09	4.7E-06	chr11	69895391	69895607	688	+	MIR	SINE
AluY_dup109463	13.02	6.75	1.17	5.78	7.4E-09	4.7E-06	chr19	22151705	22151925	1574	+	Alu	SINE
UCON22_dup27	13.20	6.76	1.17	5.77	8.0E-09	5.1E-06	chr12	72657534	72657755	719	+	UCON22	Unknown
L1PBa1_dup49	16.68	6.98	1.21	5.77	8.1E-09	5.2E-06	chr3	14098056	14100152	10756	-	L1	LINE
L1PA16_dup1222	13.62	6.79	1.18	5.76	8.2E-09	5.2E-06	chr2	75013658	75016190	12263	-	L1	LINE
AluSq2_dup20906	12.64	6.72	1.17	5.76	8.3E-09	5.2E-06	chr7	63609143	63609447	2186	+	Alu	SINE
LTR16A_dup325	162.35	8.78	1.52	5.76	8.3E-09	5.2E-06	chr1	173673907	173674344	1932	-	ERVL	LTR
L2c_dup61942	19.04	7.10	1.23	5.76	8.4E-09	5.2E-06	chr7	44909419	44909545	377	-	L2	LINE
L1MA4A_dup4381	17.46	7.02	1.22	5.76	8.5E-09	5.3E-06	chr11	4190507	4191488	5560	-	L1	LINE
L1PA6_dup5627	38.30	6.19	1.08	5.76	8.6E-09	5.3E-06	chr19	22076483	22077149	6955	-	L1	LINE
L1MD1_dup2584	118.43	1.76	0.31	5.75	8.8E-09	5.4E-06	chr6	132774702	132775673	9078	+	L1	LINE
HUERS-P3-int_dup183	2229.45	8.59	1.49	5.75	8.8E-09	5.4E-06	chr6	118901493	118910113	41307	+	ERV1	LTR
LTR9B_dup302	1377.70	9.14	1.59	5.75	9.1E-09	5.5E-06	chr6	118911405	118911869	2691	+	ERV1	LTR
L1PB1_dup11764	12.58	6.70	1.17	5.74	9.3E-09	5.7E-06	chr15	102134508	102136069	17773	-	L1	LINE
THE1B_dup2719	52.69	-7.32	1.28	-5.74	9.5E-09	5.8E-06	chr2	181781353	181781717	2241	+	ERVL-MaLR	LTR
MER11A_dup908	91.47	5.96	1.04	5.74	9.5E-09	5.8E-06	chr19	22143901	22145028	7364	-	ERVK	LTR
L1PA8_dup1499	124.57	-3.18	0.55	-5.73	9.8E-09	5.9E-06	chr3	111576531	111577328	6125	+	L1	LINE
L1PA4_dup8784	40.67	6.31	1.10	5.73	1.0E-08	6.0E-06	chr11	32544638	32546341	7644	-	L1	LINE
L1MA9_dup2925	22.64	7.24	1.26	5.73	1.0E-08	6.1E-06	chr3	110520454	110521073	2519	+	L1	LINE
AluJb_dup8505	14.15	6.81	1.19	5.73	1.0E-08	6.1E-06	chr1	150698973	150699161	1503	+	Alu	SINE
L3_dup9735	12.55	6.70	1.17	5.72	1.0E-08	6.2E-06	chr3	110585288	110585583	509	+	CR1	LINE
SVA_A_dup139	25.82	7.36	1.29	5.72	1.1E-08	6.2E-06	chrX	40721567	40722923	6841	+	SVA_A	Other
L1MEc_dup11453	19.92	7.12	1.25	5.71	1.1E-08	6.5E-06	chrX	121075350	121077058	2433	-	L1	LINE
MLT2A2_dup414	19.83	7.12	1.25	5.71	1.2E-08	6.8E-06	chr2	113621545	113622042	2175	-	ERVL	LTR
HERVIP10F-int_dup129	15.71	6.90	1.21	5.70	1.2E-08	6.9E-06	chr5	152102462	152104154	6916	+	ERV1	LTR
MLT1G3_dup1832	20.08	7.13	1.25	5.70	1.2E-08	6.9E-06	chr11	45127997	45128427	1713	-	ERVL-MaLR	LTR

MIRc_dup93316	70.39	2.50	0.44	5.70	1.2E-08	7.0E-06	chr17	73102097	73102195	223	+	MIR	SINE
L1PA2_dup3518	13.89	6.79	1.19	5.70	1.2E-08	7.1E-06	chr10	110983380	110989393	26214	+	L1	LINE
L2_dup1977	17.86	7.02	1.23	5.70	1.2E-08	7.1E-06	chr1	85643021	85643517	284	+	L2	LINE
L1MB7_dup576	349.63	4.76	0.84	5.69	1.3E-08	7.4E-06	chr1	58646267	58649172	8431	-	L1	LINE
AluSx1_dup99158	49.55	2.94	0.52	5.68	1.3E-08	7.6E-06	chr19	17524808	17525108	2271	+	Alu	SINE
MIR_dup11982	17.62	7.00	1.23	5.68	1.3E-08	7.6E-06	chr1	173616241	173616471	724	-	MIR	SINE
L1HS_dup219	14.80	6.84	1.20	5.68	1.4E-08	7.8E-06	chr2	231201785	231207228	28165	-	L1	LINE
L1PA6_dup355	13.80	6.77	1.19	5.67	1.4E-08	8.0E-06	chr1	185555537	185561623	23346	+	L1	LINE
SVA_F_dup827	12.62	6.69	1.18	5.67	1.4E-08	8.0E-06	chr16	28708507	28710833	7421	-	SVA_F	Other
MIRc_dup66681	393.36	-6.14	1.08	-5.67	1.4E-08	8.1E-06	chr11	4675282	4675442	313	-	MIR	SINE
L1P2_dup1477	11.77	6.62	1.17	5.66	1.5E-08	8.4E-06	chr19	22134477	22136561	15113	+	L1	LINE
BLACKJACK_dup258	103.95	4.70	0.83	5.66	1.5E-08	8.4E-06	chr2	168838108	168839059	2545	+	hAT-Blackjack	DNA
L1PA4_dup8846	29.18	6.12	1.08	5.65	1.6E-08	9.0E-06	chr11	45143865	45150048	27256	-	L1	LINE
Tigger15a_dup2637	130.77	-2.37	0.42	-5.65	1.6E-08	9.1E-06	chr8	63894424	63894542	228	+	TcMar-Tigger	DNA
Charlie21a_dup383	40.65	-7.08	1.25	-5.65	1.6E-08	9.2E-06	chr7	94340798	94341044	575	-	hAT-Charlie	DNA
L1M2_dup1639	13.22	6.72	1.19	5.63	1.8E-08	9.7E-06	chr3	110662644	110666093	4226	+	L1	LINE
L1ME1_dup654	223.84	5.17	0.92	5.63	1.8E-08	9.7E-06	chr1	58242030	58244281	4872	-	L1	LINE
L1PA3_dup5587	13.51	6.74	1.20	5.63	1.8E-08	9.7E-06	chr8	127353831	127359842	25969	+	L1	LINE
AluJr_dup31787	63.10	-7.39	1.31	-5.63	1.8E-08	9.7E-06	chr7	145871019	145871323	1624	+	Alu	SINE
L1P1_dup2706	12.68	6.68	1.19	5.63	1.8E-08	9.9E-06	chr14	56328343	56330256	15651	+	L1	LINE
L2_dup40417	458.96	-1.68	0.30	-5.62	1.9E-08	1.0E-05	chr11	125526532	125526676	419	+	L2	LINE
L1PA5_dup1404	53.44	-7.27	1.29	-5.62	1.9E-08	1.0E-05	chr2	181450347	181456444	25041	-	L1	LINE
AluSq2_dup3344	19.26	7.06	1.26	5.61	2.0E-08	1.1E-05	chr1	173613852	173614149	2230	+	Alu	SINE
SVA_B_dup118	44.98	6.39	1.14	5.61	2.0E-08	1.1E-05	chr4	78927720	78929041	10595	-	SVA_B	Other
HERVI-int_dup7	296.09	7.99	1.42	5.61	2.0E-08	1.1E-05	chr1	173607757	173611588	11658	+	ERV1	LTR
HERV16-int_dup79	127.75	8.54	1.53	5.59	2.2E-08	1.2E-05	chr1	173672647	173673007	715	-	ERVL	LTR
HERVH-int_dup1259	14.72	6.81	1.22	5.59	2.2E-08	1.2E-05	chr3	128683423	128683802	2478	+	ERV1	LTR
L1ME3D_dup1753	23.77	7.23	1.30	5.58	2.4E-08	1.3E-05	chr6	132560427	132561312	555	+	L1	LINE
L3_dup30853	20.25	7.09	1.27	5.58	2.5E-08	1.3E-05	chr11	27147196	27147302	180	+	CR1	LINE
L2b_dup59693	292.34	5.19	0.93	5.57	2.5E-08	1.3E-05	chr10	99625065	99625177	350	+	L2	LINE
L2b_dup45186	512.70	-2.33	0.42	-5.57	2.5E-08	1.3E-05	chr8	63895728	63895849	383	-	L2	LINE
HERVIP10F-int_dup358	35.96	6.01	1.08	5.57	2.6E-08	1.4E-05	chr16	30111384	30112322	5185	+	ERV1	LTR
L1PA3_dup5611	12.02	6.61	1.19	5.57	2.6E-08	1.4E-05	chr8	132647419	132653435	27115	+	L1	LINE
MER4E1_dup529	21.22	7.13	1.28	5.57	2.6E-08	1.4E-05	chr9	138538454	138539210	5147	+	ERV1	LTR
L2c_dup18519	42.76	2.51	0.45	5.56	2.7E-08	1.4E-05	chr2	54082541	54082883	183	-	L2	LINE
L1PA13_dup1678	46.57	-7.14	1.29	-5.56	2.7E-08	1.4E-05	chr3	178050446	178053201	8620	-	L1	LINE
L1MEc_dup7541	23.59	7.22	1.30	5.56	2.7E-08	1.4E-05	chr6	132603861	132605850	6097	+	L1	LINE
L1MA4A_dup3754	19.78	7.06	1.27	5.55	2.8E-08	1.5E-05	chrX	82088453	82090781	8253	+	L1	LINE
MIRb_dup121668	242.32	3.54	0.64	5.55	2.9E-08	1.5E-05	chr9	127576731	127576872	431	-	MIR	SINE
MIRc_dup100316	20.81	7.10	1.28	5.54	3.0E-08	1.5E-05	chr21	34163614	34163661	190	+	MIR	SINE
PABL_B_dup187	22.81	7.18	1.30	5.54	3.0E-08	1.6E-05	chr9	138554137	138554765	3987	+	ERV1	LTR
L1MA3_dup4858	12.96	6.67	1.21	5.54	3.1E-08	1.6E-05	chr9	36181629	36182179	6264	+	L1	LINE

AluSx_dup92570	874.86	-1.72	0.31	-5.54	3.1E-08	1.6E-05	chr12	76421260	76421564	2555	+	Alu	SINE
L1PA16_dup3653	80.98	-7.49	1.35	-5.53	3.1E-08	1.6E-05	chr4	104376784	104378248	5609	+	L1	LINE
L1M5_dup59470	13.14	6.68	1.21	5.53	3.2E-08	1.6E-05	chr19	9573559	9573747	308	+	L1	LINE
L1PA2_dup2836	61.63	5.74	1.04	5.53	3.2E-08	1.6E-05	chr9	126665650	126671644	28418	+	L1	LINE
HERVIP10FH-int_dup50	18.71	7.00	1.27	5.53	3.2E-08	1.6E-05	chr1	173606964	173607234	759	+	ERV1	LTR
L1MB5_dup4695	14.02	6.74	1.22	5.53	3.2E-08	1.6E-05	chrX	82078087	82079276	9993	-	L1	LINE
SVA_D_dup1264	21.72	7.13	1.29	5.53	3.3E-08	1.6E-05	chr19	39287788	39289464	8634	-	SVA_D	Other
L1ME3_dup4895	12.52	6.64	1.20	5.53	3.3E-08	1.6E-05	chr9	126671708	126672936	4769	+	L1	LINE
MamRep1161_dup1719	18.69	7.00	1.27	5.52	3.3E-08	1.7E-05	chr21	34164653	34164777	345	+	TcMar	DNA
HERV3-int_dup42	13.26	6.69	1.21	5.52	3.4E-08	1.7E-05	chr3	128575651	128576307	14138	-	ERV1	LTR
LTR10G_dup39	17.59	6.95	1.26	5.52	3.4E-08	1.7E-05	chr6	118895207	118895666	1346	+	ERV1	LTR
SVA_D_dup1153	15.65	6.84	1.24	5.52	3.4E-08	1.7E-05	chr17	3756901	3758383	11001	+	SVA_D	Other
MER50_dup39	15.75	6.84	1.24	5.51	3.5E-08	1.7E-05	chr1	58462589	58463261	3601	+	ERV1	LTR
HERVP71A-int_dup195	34.06	4.15	0.75	5.51	3.5E-08	1.7E-05	chr16	30112597	30113550	4877	+	ERV1	LTR
AluSz_dup36160	12.24	6.61	1.20	5.51	3.6E-08	1.7E-05	chr7	63609593	63609883	1805	+	Alu	SINE
L1PREC2_dup7400	11.27	6.53	1.19	5.51	3.6E-08	1.7E-05	chr19	22182859	22183808	14258	+	L1	LINE
L1MEf_dup2724	16.13	6.86	1.25	5.50	3.7E-08	1.8E-05	chr3	110594234	110594371	290	-	L1	LINE
L1MA5_dup855	319.93	-4.01	0.73	-5.49	3.9E-08	1.9E-05	chr3	111943007	111945286	8044	+	L1	LINE
MER4E1_dup208	52.49	-4.83	0.88	-5.49	4.1E-08	2.0E-05	chr3	124470223	124470998	4700	+	ERV1	LTR
L1PA5_dup1405	39.91	-7.00	1.28	-5.48	4.2E-08	2.0E-05	chr2	181785935	181786454	4288	-	L1	LINE
L1PA3_dup9389	14.05	6.73	1.23	5.48	4.2E-08	2.0E-05	chr14	90673118	90679148	28165	-	L1	LINE
L1ME1_dup6467	269.86	-3.58	0.65	-5.48	4.3E-08	2.1E-05	chr3	111853413	111853971	2822	+	L1	LINE
MER5B_dup16524	472.30	-5.60	1.02	-5.48	4.4E-08	2.1E-05	chr11	4675668	4675814	270	-	hAT-Charlie	DNA
AluSx_dup129146	11.59	6.55	1.20	5.47	4.4E-08	2.1E-05	chr19	22383318	22383650	1727	-	Alu	SINE
MSTA-int_dup2552	11.70	6.56	1.20	5.47	4.4E-08	2.1E-05	chr13	52670364	52671955	9112	+	ERVL-MaLR	LTR
AluSc_dup31281	69.10	3.39	0.62	5.47	4.5E-08	2.1E-05	chr19	17528694	17529003	2368	-	Alu	SINE
LTR9_dup1853	13.04	6.65	1.22	5.47	4.6E-08	2.2E-05	chr19	22096275	22096898	1988	+	ERV1	LTR
L2c_dup11137	14.10	6.72	1.23	5.46	4.8E-08	2.3E-05	chr1	173630407	173630560	259	-	L2	LINE
L1PA5_dup5340	104.13	-3.35	0.61	-5.46	4.8E-08	2.3E-05	chr7	145854374	145858519	16620	+	L1	LINE
MIR_dup106524	275.78	-2.56	0.47	-5.46	4.9E-08	2.3E-05	chr10	61410945	61410992	221	+	MIR	SINE
L1PA3_dup1089	14.55	6.75	1.24	5.45	5.1E-08	2.4E-05	chr2	105828427	105830947	18632	-	L1	LINE
MER57-int_dup163	72.31	2.98	0.55	5.45	5.1E-08	2.4E-05	chr2	168829257	168831717	13098	+	ERV1	LTR
L1MC4_dup17404	11.67	6.55	1.20	5.45	5.2E-08	2.4E-05	chrX	130794480	130794898	912	-	L1	LINE
HERVIP10FH-int_dup672	59.54	5.03	0.92	5.44	5.3E-08	2.5E-05	chr22	42500961	42503342	8869	-	ERV1	LTR
AluSx_dup69505	20.54	7.05	1.30	5.44	5.4E-08	2.5E-05	chr9	138550189	138550492	2189	+	Alu	SINE
MER107_dup77	115.87	2.70	0.50	5.43	5.5E-08	2.5E-05	chr4	89427098	89427246	1018	-	DNA	DNA
L1M3a_dup543	60.98	5.66	1.04	5.43	5.7E-08	2.6E-05	chr19	22168927	22169624	1857	+	L1	LINE
LTR10C_dup100	13.14	6.65	1.23	5.43	5.7E-08	2.6E-05	chr3	110688579	110689151	3706	-	ERV1	LTR
L1M5_dup63265	16.58	-6.20	1.14	-5.42	5.9E-08	2.7E-05	chr22	32669175	32669409	696	+	L1	LINE
L1PB1_dup9577	11.30	6.51	1.20	5.42	5.9E-08	2.7E-05	chr11	5925830	5930244	22199	-	L1	LINE
L1PA2_dup1244	72.46	-7.36	1.36	-5.42	6.0E-08	2.7E-05	chr4	104171423	104177447	27379	-	L1	LINE
L1PA6_dup910	109.51	3.00	0.55	5.42	6.1E-08	2.8E-05	chr2	231390159	231396267	23877	+	L1	LINE

L1MC_dup11201	11.33	6.51	1.20	5.41	6.2E-08	2.8E-05	chr20	4016611	4016699	428	-	L1	LINE
L1MA4A_dup115	17.19	6.89	1.27	5.41	6.3E-08	2.8E-05	chr1	58005843	58006793	5979	+	L1	LINE
LTR76_dup179	20.62	7.04	1.30	5.41	6.3E-08	2.8E-05	chr21	39595965	39596364	1365	-	ERV1	LTR
MLT1G_dup2589	16.51	6.85	1.27	5.41	6.4E-08	2.9E-05	chr19	7884088	7884713	2209	+	ERVL-MaLR	LTR
L1ME3C_dup12183	25.31	7.22	1.33	5.41	6.4E-08	2.9E-05	chr21	29541999	29542185	360	+	L1	LINE
L1MA9_dup11686	15.15	6.77	1.25	5.41	6.5E-08	2.9E-05	chr11	32536527	32536742	920	-	L1	LINE
L1PA4_dup8408	13.22	6.65	1.23	5.41	6.5E-08	2.9E-05	chr10	64709387	64715527	26926	+	L1	LINE
LTR13_dup406	10.77	6.46	1.19	5.41	6.5E-08	2.9E-05	chr19	22072324	22073344	7610	-	ERVK	LTR
L2b_dup64871	13.45	6.66	1.23	5.40	6.5E-08	2.9E-05	chr11	85824762	85825505	1480	+	L2	LINE
L1ME1_dup6450	12.36	6.59	1.22	5.40	6.6E-08	2.9E-05	chr3	110548895	110549468	1815	-	L1	LINE
L1PA4_dup3369	14.74	6.75	1.25	5.40	6.6E-08	2.9E-05	chr4	159465602	159471751	25332	-	L1	LINE
MLT1G1_dup752	10.81	6.46	1.20	5.40	6.7E-08	2.9E-05	chr3	110599888	110600362	1880	+	ERVL-MaLR	LTR
AluSx1_dup71433	557.29	4.02	0.74	5.39	6.9E-08	3.0E-05	chr12	69249510	69249812	2253	-	Alu	SINE
MLT1A0_dup1709	449.62	-3.07	0.57	-5.39	7.0E-08	3.1E-05	chr2	14789881	14790233	1972	+	ERVL-MaLR	LTR
L2b_dup4052	16.50	6.84	1.27	5.39	7.2E-08	3.1E-05	chr1	57963443	57964645	1725	-	L2	LINE
L2_dup40785	19.14	6.97	1.29	5.38	7.3E-08	3.2E-05	chr12	10047844	10048140	383	-	L2	LINE
Charlie1_dup297	12.05	6.55	1.22	5.38	7.5E-08	3.3E-05	chr2	168789455	168789611	707	+	hAT-Charlie	DNA
L1PA11_dup3345	160.95	-2.94	0.55	-5.38	7.6E-08	3.3E-05	chr12	130636017	130636338	2232	+	L1	LINE
MLT1I_dup5650	13.14	6.63	1.23	5.38	7.6E-08	3.3E-05	chr8	124222036	124222376	843	+	ERVL-MaLR	LTR
L1MA6_dup2407	10.78	6.45	1.20	5.37	7.7E-08	3.3E-05	chr7	63630305	63631821	9693	-	L1	LINE
AluJb_dup91510	21.51	7.07	1.31	5.37	7.7E-08	3.3E-05	chr12	10046771	10047080	1883	-	Alu	SINE
AluJr4_dup11101	17.29	6.87	1.28	5.37	8.0E-08	3.4E-05	chr11	32507772	32508064	1842	-	Alu	SINE
MER50-int_dup474	10.78	6.45	1.20	5.36	8.3E-08	3.5E-05	chr19	22094925	22095694	4658	-	ERV1	LTR
L2b_dup65913	94.68	5.13	0.96	5.36	8.4E-08	3.6E-05	chr11	114648612	114649443	582	-	L2	LINE
L1PA2_dup2712	29.89	6.08	1.14	5.35	8.7E-08	3.7E-05	chr9	32729015	32735045	28232	+	L1	LINE
MARNA_dup1737	37.88	-3.45	0.65	-5.35	8.8E-08	3.7E-05	chr8	70767045	70767425	812	+	cMar-Marine	DNA
L1PA5_dup5630	290.53	-2.60	0.49	-5.35	9.0E-08	3.8E-05	chr8	70755536	70761658	25865	+	L1	LINE
MIRb_dup48438	14.12	6.69	1.25	5.35	9.0E-08	3.8E-05	chr3	110526481	110526592	229	+	MIR	SINE
MIRc_dup14238	11.15	6.47	1.21	5.34	9.3E-08	3.9E-05	chr2	86131565	86131772	692	+	MIR	SINE
L1MEc_dup3972	54.85	5.84	1.09	5.34	9.3E-08	3.9E-05	chr3	110546034	110547027	2438	-	L1	LINE
L1PA3_dup535	10.85	6.44	1.21	5.34	9.4E-08	3.9E-05	chr1	177370054	177376077	26193	-	L1	LINE
L1PA3_dup4577	11.22	6.48	1.21	5.34	9.4E-08	3.9E-05	chr7	80342422	80348461	27270	+	L1	LINE
L2c_dup105282	418.54	3.96	0.74	5.34	9.5E-08	4.0E-05	chr12	69248940	69249215	852	-	L2	LINE
L1M4_dup5518	19.16	6.95	1.30	5.33	9.6E-08	4.0E-05	chr6	35518219	35518712	4155	+	L1	LINE
MSTA_dup19302	50.35	6.14	1.15	5.33	9.7E-08	4.0E-05	chr21	39513161	39513538	2233	+	ERVL-MaLR	LTR
MER44A_dup155	16.07	6.80	1.28	5.33	9.8E-08	4.1E-05	chr1	222010083	222010390	1618	+	TcMar-Tigger	DNA
L2_dup11394	12.47	6.57	1.23	5.33	9.8E-08	4.1E-05	chr3	110572662	110573274	2036	+	L2	LINE
L1PA2_dup2777	12.66	6.58	1.24	5.32	1.0E-07	4.2E-05	chr9	83269032	83275050	27200	-	L1	LINE
LTR9B_dup301	18.64	6.92	1.30	5.32	1.1E-07	4.3E-05	chr6	118900874	118901492	4183	+	ERV1	LTR
L1PA4_dup9772	18.03	6.89	1.30	5.32	1.1E-07	4.3E-05	chr12	129577793	129579737	9950	+	L1	LINE
SVA_D_dup946	21.50	7.04	1.33	5.32	1.1E-07	4.3E-05	chr12	76334786	76336717	7191	+	SVA_D	Other
HERVI-int_dup22	490.85	6.90	1.30	5.31	1.1E-07	4.4E-05	chr3	110683916	110687774	12846	-	ERV1	LTR

LTR37A_dup848	61.68	-7.20	1.36	-5.31	1.1E-07	4.4E-05	chr7	94338280	94338679	1242	+	ERV1	LTR
L1PB4_dup7074	11.53	6.49	1.22	5.30	1.1E-07	4.6E-05	chr19	7849450	7849522	357	-	L1	LINE
FLAM_A_dup13359	21.11	-6.39	1.20	-5.30	1.1E-07	4.6E-05	chr16	28297580	28297708	858	+	Alu	SINE
L1PA5_dup8166	64.97	5.01	0.95	5.29	1.2E-07	4.8E-05	chr11	32512311	32514316	10231	-	L1	LINE
L1MA4_dup9516	11.88	6.51	1.23	5.29	1.2E-07	4.9E-05	chr19	22076187	22076473	1261	-	L1	LINE
L1PA3_dup7663	11.71	6.50	1.23	5.28	1.3E-07	5.1E-05	chr10	106288093	106294135	28179	-	L1	LINE
AluSx3_dup2216	16.33	6.79	1.29	5.28	1.3E-07	5.1E-05	chr1	222091778	222092080	2228	+	Alu	SINE
Charlie2b_dup2819	18.05	6.88	1.30	5.27	1.3E-07	5.4E-05	chr13	52704308	52704896	1519	-	hAT-Charlie	DNA
BSR/Beta_dup1595	10.45	6.39	1.21	5.27	1.3E-07	5.4E-05	chr19	22105160	22107067	1452	-	Satellite	Satellite
AluY_dup22722	12.31	6.54	1.24	5.27	1.4E-07	5.6E-05	chr3	110603608	110603912	2624	+	Alu	SINE
MIR3_dup32684	18.26	-6.24	1.19	-5.26	1.4E-07	5.6E-05	chr6	3229776	3229888	280	-	MIR	SINE
MIRb_dup88329	83.60	-4.95	0.94	-5.26	1.4E-07	5.6E-05	chr6	127520211	127520404	504	+	MIR	SINE
AluJo_dup32044	298.97	-4.96	0.94	-5.26	1.4E-07	5.7E-05	chr8	117850461	117850777	1611	-	Alu	SINE
L1MDa_dup1411	37.67	-6.85	1.30	-5.26	1.4E-07	5.7E-05	chr3	117363162	117364211	7452	-	L1	LINE
MIRc_dup100159	11.19	6.45	1.23	5.26	1.5E-07	5.7E-05	chr21	29112759	29112949	199	+	MIR	SINE
L1MC_dup855	58.51	-3.82	0.73	-5.25	1.5E-07	5.8E-05	chr2	14807056	14807595	497	-	L1	LINE
L1MDa_dup1408	30.01	-6.67	1.27	-5.25	1.5E-07	5.8E-05	chr3	117357023	117358431	4278	-	L1	LINE
HERVIP10F-int_dup155	1481.24	8.24	1.57	5.25	1.5E-07	5.8E-05	chr6	118897961	118899240	4906	+	ERV1	LTR
L1M1_dup1118	66.68	-7.20	1.37	-5.25	1.5E-07	5.9E-05	chr2	186447461	186449776	7482	+	L1	LINE
AluSx_dup129129	10.35	6.37	1.21	5.25	1.5E-07	5.9E-05	chr19	22151979	22152079	584	-	Alu	SINE
L1PA7_dup2457	42.87	5.95	1.13	5.25	1.5E-07	5.9E-05	chr3	110562462	110564714	10627	+	L1	LINE
MIR3_dup6588	11.90	6.50	1.24	5.25	1.6E-07	6.0E-05	chr1	173632097	173632136	198	-	MIR	SINE
MLT1A1_dup331	106.72	8.15	1.56	5.24	1.6E-07	6.2E-05	chr1	173677349	173677741	1826	+	ERVL-MaLR	LTR
L2c_dup11133	80.11	-3.84	0.73	-5.24	1.6E-07	6.2E-05	chr1	173576616	173576837	326	-	L2	LINE
Ricksha_0_dup164	103.26	-4.69	0.89	-5.24	1.6E-07	6.2E-05	chr6	134224424	134225321	5132	-	MuDR	DNA
L1MEf_dup11069	11.93	6.50	1.24	5.23	1.7E-07	6.3E-05	chr14	56426770	56428588	4723	+	L1	LINE
HERVL-int_dup2207	10.19	6.35	1.21	5.23	1.7E-07	6.4E-05	chr21	29399566	29401840	16997	+	ERVL	LTR
FAM_dup2903	10.01	6.34	1.21	5.23	1.7E-07	6.4E-05	chr10	42904149	42904298	811	-	Alu	SINE
MIR3_dup67670	444.63	4.01	0.77	5.23	1.7E-07	6.4E-05	chr12	113357377	113357525	358	-	MIR	SINE
L1M5_dup12474	11.76	6.48	1.24	5.23	1.7E-07	6.6E-05	chr3	110547077	110547512	357	-	L1	LINE
L1PA2_dup4358	10.81	6.41	1.23	5.23	1.7E-07	6.6E-05	chr15	55356192	55362189	27099	+	L1	LINE
LTR8A_dup381	78.25	5.42	1.04	5.22	1.8E-07	6.6E-05	chr2	168832792	168833456	3148	+	ERV1	LTR
L1PA8_dup5802	10.40	6.37	1.22	5.22	1.8E-07	6.7E-05	chr11	57753969	57758219	22035	-	L1	LINE
L1PA6_dup2851	13.76	6.62	1.27	5.22	1.8E-07	6.8E-05	chr7	85304267	85306850	13694	-	L1	LINE
L1PA2_dup946	11.18	6.44	1.23	5.22	1.8E-07	6.8E-05	chr3	145645566	145651578	27481	+	L1	LINE
AluSc_dup2283	14.35	6.66	1.28	5.22	1.8E-07	6.8E-05	chr1	206279135	206279440	2310	-	Alu	SINE
L4_dup12427	185.79	3.69	0.71	5.22	1.8E-07	6.8E-05	chr12	15749895	15749964	197	+	RTE	LINE
LTR50_dup2205	23.30	-6.44	1.23	-5.22	1.8E-07	6.8E-05	chr15	37278461	37279108	1369	+	ERVL	LTR
SVA_D_dup1140	25.39	5.88	1.13	5.21	1.8E-07	6.9E-05	chr17	474751	476282	10298	+	SVA_D	Other
SVA_F_dup709	13.15	6.58	1.26	5.21	1.9E-07	7.1E-05	chr12	56468876	56470558	10205	-	SVA_F	Other
HERVL-int_dup2208	10.73	6.39	1.23	5.21	1.9E-07	7.1E-05	chr21	29409417	29412755	24109	-	ERVL	LTR
MER21B_dup97	10.97	6.41	1.23	5.20	2.0E-07	7.2E-05	chr1	112141210	112141818	4033	-	ERVL	LTR

L1MB2_dup852	29.12	2.74	0.53	5.20	2.0E-07	7.2E-05	chr2	86075666	86075987	1722	+	L1	LINE
AluSx_dup99359	10.50	6.37	1.23	5.20	2.0E-07	7.3E-05	chr14	20982554	20982853	2158	+	Alu	SINE
L1PA3_dup10210	10.17	6.34	1.22	5.19	2.1E-07	7.6E-05	chr19	22171889	22174108	12026	-	L1	LINE
L1MA8_dup8482	47.50	-6.97	1.34	-5.19	2.1E-07	7.8E-05	chr13	39198527	39198805	3095	+	L1	LINE
AluSx_dup127845	13.70	6.61	1.27	5.18	2.2E-07	8.0E-05	chr19	14454433	14454517	1863	+	Alu	SINE
L1PA5_dup1119	15.75	6.72	1.30	5.18	2.2E-07	8.1E-05	chr2	113656493	113657198	5698	-	L1	LINE
L2c_dup105280	356.12	4.04	0.78	5.18	2.3E-07	8.2E-05	chr12	69248070	69248420	623	-	L2	LINE
L1PA3_dup9439	33.74	5.85	1.13	5.17	2.3E-07	8.4E-05	chr15	27432874	27439019	25633	-	L1	LINE
AluJo_dup59036	12.49	6.52	1.26	5.17	2.3E-07	8.4E-05	chr17	5437400	5437713	2026	+	Alu	SINE
AluSg4_dup455	12.90	6.55	1.27	5.17	2.3E-07	8.4E-05	chr1	173612311	173612607	2310	-	Alu	SINE
AluSz_dup58733	16.74	6.77	1.31	5.17	2.3E-07	8.4E-05	chr11	32506535	32506666	1034	-	Alu	SINE
FLAM_A_dup6112	16.74	6.77	1.31	5.17	2.4E-07	8.4E-05	chr6	110713711	110713841	630	-	Alu	SINE
L1MC5_dup3290	39.27	-6.83	1.32	-5.17	2.4E-07	8.4E-05	chr3	10309711	10309802	330	-	L1	LINE
L2c_dup69046	312.74	-2.25	0.44	-5.17	2.4E-07	8.4E-05	chr8	63896010	63896078	186	-	L2	LINE
Tigger3_dup129	31.74	-6.68	1.29	-5.17	2.4E-07	8.4E-05	chr2	181619895	181622072	17257	-	TcMar-Tigger	DNA
Charlie2b_dup2818	28.37	5.85	1.13	5.17	2.4E-07	8.5E-05	chr13	52703735	52703949	283	-	hAT-Charlie	DNA
L1MD1_dup6273	31.52	-6.67	1.29	-5.17	2.4E-07	8.5E-05	chr18	50041997	50042782	2626	+	L1	LINE
L1M1_dup2375	70.89	-7.14	1.38	-5.16	2.4E-07	8.7E-05	chr4	104300930	104302729	14260	+	L1	LINE
AluSx1_dup100492	10.21	6.33	1.23	5.16	2.5E-07	8.7E-05	chr19	37287103	37287484	1684	-	Alu	SINE
L1M4_dup2159	13.35	6.57	1.27	5.16	2.5E-07	8.7E-05	chr2	168750351	168751085	4872	-	L1	LINE
MER39_dup202	16.47	6.75	1.31	5.16	2.5E-07	8.9E-05	chr1	222011950	222012307	1329	-	ERV1	LTR
L1MCa_dup648	10.31	6.34	1.23	5.15	2.5E-07	8.9E-05	chr2	74839917	74840966	5493	-	L1	LINE
L2c_dup4508	13.31	6.57	1.27	5.15	2.5E-07	8.9E-05	chr1	58102912	58103318	188	-	L2	LINE
L1PA4_dup9835	12.19	6.49	1.26	5.15	2.5E-07	9.0E-05	chr13	31914729	31920657	25881	+	L1	LINE
MER91B_dup751	197.33	-2.25	0.44	-5.15	2.6E-07	9.1E-05	chr8	63894758	63894825	202	-	hAT-Tip100	DNA
MIR3_dup19592	20.19	-6.29	1.22	-5.15	2.6E-07	9.2E-05	chr3	111842285	111842332	223	-	MIR	SINE
MLT1B_dup10020	142.29	8.14	1.58	5.15	2.6E-07	9.2E-05	chr9	138557978	138558384	393	-	ERVL-MaLR	LTR
HERVL40-int_dup183	10.87	6.39	1.24	5.15	2.7E-07	9.3E-05	chr3	110587736	110588411	1955	+	ERVL	LTR
L1M5_dup4996	517.93	-3.55	0.69	-5.14	2.7E-07	9.3E-05	chr2	14790765	14791004	314	-	L1	LINE
SVA_F_dup823	10.45	6.35	1.23	5.14	2.7E-07	9.5E-05	chr16	24119671	24121462	9304	-	SVA_F	Other
AluSc_dup12562	147.43	8.13	1.58	5.14	2.8E-07	9.6E-05	chr6	118899613	118899912	2471	+	Alu	SINE
L1PA8A_dup1949	101.83	4.34	0.84	5.14	2.8E-07	9.6E-05	chr12	20220673	20225799	21328	+	L1	LINE
SVA_F_dup421	12.59	6.51	1.27	5.14	2.8E-07	9.6E-05	chr7	73250659	73251631	6605	+	SVA_F	Other
L1MC4a_dup1275	84.80	7.98	1.55	5.13	2.8E-07	9.8E-05	chr1	173677029	173677348	677	-	L1	LINE
L2b_dup89957	10.06	6.31	1.23	5.13	2.9E-07	9.8E-05	chr19	22122935	22123056	245	+	L2	LINE
HERVL40-int_dup185	9.86	6.29	1.23	5.13	2.9E-07	9.9E-05	chr3	110588862	110589483	1884	+	ERVL	LTR
LTR1B_dup219	12.26	6.49	1.26	5.13	2.9E-07	1.0E-04	chr3	110677326	110678065	4102	-	ERV1	LTR
MIRb_dup93594	11.57	6.43	1.26	5.12	3.0E-07	1.0E-04	chr7	38343487	38343699	245	-	MIR	SINE
THE1B_dup1290	14.13	6.61	1.29	5.12	3.1E-07	1.0E-04	chr1	222237943	222238295	2322	-	ERVL-MaLR	LTR
AluSx_dup126545	11.85	6.45	1.26	5.11	3.2E-07	1.1E-04	chr19	7854824	7855095	2120	+	Alu	SINE
Tigger15a_dup2636	180.85	-2.65	0.52	-5.11	3.3E-07	1.1E-04	chr8	63894273	63894381	301	+	TcMar-Tigger	DNA
AluSx1_dup99701	35.80	5.23	1.03	5.10	3.4E-07	1.2E-04	chr19	22360320	22360618	2006	+	Alu	SINE

L4_dup10056	442.21	3.30	0.65	5.10	3.4E-07	1.2E-04	chr9	127576902	127577135	355	+	RTE	LINE
LTR34_dup330	65.65	2.96	0.58	5.10	3.4E-07	1.2E-04	chr19	17523462	17523936	990	-	ERV1	LTR
AluSx_dup126852	30.89	5.75	1.13	5.09	3.5E-07	1.2E-04	chr19	9576387	9576690	2262	+	Alu	SINE
MLT1J2_dup4952	18.27	6.81	1.34	5.09	3.5E-07	1.2E-04	chr12	15754726	15754892	490	+	ERVL-MaLR	LTR
AluSx_dup21939	16.96	-6.13	1.20	-5.09	3.5E-07	1.2E-04	chr2	239753319	239753627	2267	-	Alu	SINE
LTR2B_dup213	13.19	6.53	1.29	5.08	3.7E-07	1.2E-04	chr10	85926942	85927425	4054	-	ERV1	LTR
AluSx1_dup65453	13.57	6.56	1.29	5.08	3.7E-07	1.2E-04	chr11	32518623	32518918	2272	+	Alu	SINE
L2a_dup42343	15.42	6.67	1.31	5.08	3.7E-07	1.3E-04	chr4	25249921	25250145	285	-	L2	LINE
MER50_dup105	13.43	6.55	1.29	5.08	3.8E-07	1.3E-04	chr1	173641041	173641267	3716	+	ERV1	LTR
L1MD2_dup4637	64.75	2.20	0.43	5.08	3.8E-07	1.3E-04	chr7	128816576	128817497	5010	-	L1	LINE
L1PA6_dup1374	52.84	4.48	0.88	5.08	3.8E-07	1.3E-04	chr4	6837193	6838107	6944	-	L1	LINE
AluSx_dup10117	66.40	-3.08	0.61	-5.08	3.8E-07	1.3E-04	chr1	202820441	202820745	2128	+	Alu	SINE
L1PA3_dup8795	22.39	5.72	1.13	5.08	3.9E-07	1.3E-04	chr13	25380786	25386806	27465	-	L1	LINE
L2a_dup35638	45.35	-4.34	0.85	-5.07	3.9E-07	1.3E-04	chr3	111854077	111854175	344	-	L2	LINE
L1MA9_dup6066	12.76	6.50	1.28	5.07	3.9E-07	1.3E-04	chr6	65014165	65015003	1907	+	L1	LINE
L1ME1_dup12540	59.60	-7.02	1.38	-5.07	3.9E-07	1.3E-04	chr6	134250088	134250728	2782	-	L1	LINE
L1PA12_dup1348	31.88	5.18	1.02	5.07	3.9E-07	1.3E-04	chr11	86594213	86594725	3795	+	L1	LINE
L1PA3_dup8028	15.52	6.67	1.31	5.07	3.9E-07	1.3E-04	chr11	58463759	58468139	25733	-	L1	LINE
MARNA_dup2876	77.77	4.41	0.87	5.07	4.0E-07	1.3E-04	chr15	90208369	90208783	550	-	cMar-Marine	DNA
SVA_F_dup835	11.16	6.38	1.26	5.07	4.0E-07	1.3E-04	chr16	56773650	56774916	5405	-	SVA_F	Other
L1ME1_dup20896	28.88	-6.56	1.29	-5.07	4.0E-07	1.3E-04	chr10	130521374	130521549	734	+	L1	LINE
L1ME2_dup6257	94.82	-2.26	0.45	-5.07	4.1E-07	1.3E-04	chr8	63878984	63879213	963	-	L1	LINE
L1PA3_dup8176	10.89	6.36	1.26	5.06	4.2E-07	1.3E-04	chr11	101723221	101729358	26805	+	L1	LINE
HAL1_dup4168	29.07	-6.56	1.30	-5.06	4.2E-07	1.4E-04	chr2	181765161	181765729	2646	+	L1	LINE
L1ME4a_dup35179	23.13	-6.38	1.26	-5.06	4.2E-07	1.4E-04	chr15	37315461	37315705	282	-	L1	LINE
L1PA3_dup9641	14.53	6.61	1.31	5.06	4.2E-07	1.4E-04	chr15	98634769	98640793	27338	+	L1	LINE
LTR28_dup506	10.12	6.29	1.24	5.06	4.2E-07	1.4E-04	chr21	29116385	29117272	4717	-	ERV1	LTR
SVA_D_dup853	10.83	6.35	1.26	5.05	4.3E-07	1.4E-04	chr11	34007129	34008566	10640	+	SVA_D	Other
AluSx_dup16838	479.38	3.72	0.74	5.05	4.5E-07	1.4E-04	chr2	102793444	102793731	2203	+	Alu	SINE
MIR_dup148262	391.77	-1.11	0.22	-5.05	4.5E-07	1.4E-04	chr16	30434927	30435159	785	-	MIR	SINE
L1MA9_dup6068	12.45	6.47	1.28	5.04	4.6E-07	1.5E-04	chr6	65017212	65017756	1631	+	L1	LINE
MER4-int_dup167	12.96	6.50	1.29	5.04	4.6E-07	1.5E-04	chr1	173643991	173644469	15271	-	ERV1	LTR
L1PA13_dup6443	13.32	6.53	1.29	5.04	4.6E-07	1.5E-04	chr11	6000001	6004318	9983	-	L1	LINE
L1PA2_dup3425	14.27	6.59	1.31	5.04	4.7E-07	1.5E-04	chr10	38284372	38290379	27762	+	L1	LINE
LTR70_dup111	53.71	5.89	1.17	5.03	4.9E-07	1.5E-04	chr19	22384404	22385698	7838	-	ERV1	LTR
L1PA2_dup3472	14.19	6.58	1.31	5.03	4.9E-07	1.6E-04	chr10	82482266	82488300	28259	-	L1	LINE
AluJb_dup130607	29.36	2.80	0.56	5.02	5.1E-07	1.6E-04	chr19	17524383	17524530	1169	+	Alu	SINE
L1MA3_dup4696	15.09	-5.99	1.19	-5.02	5.3E-07	1.7E-04	chr8	138756931	138758590	16153	-	L1	LINE
L1PA6_dup3606	12.10	6.43	1.28	5.02	5.3E-07	1.7E-04	chrX	62697902	62699113	17118	+	L1	LINE
L1MB8_dup6748	61.78	-6.98	1.39	-5.01	5.4E-07	1.7E-04	chr7	124815611	124816351	2962	+	L1	LINE
LTR13_dup384	10.66	6.32	1.26	5.01	5.6E-07	1.7E-04	chr19	7857519	7858528	8021	-	ERVK	LTR
AluSq2_dup2825	32.03	5.90	1.18	5.01	5.6E-07	1.7E-04	chr1	150699529	150699823	2459	-	Alu	SINE

L3_dup9733	9.96	6.26	1.25	5.00	5.6E-07	1.8E-04	chr3	110583346	110583519	472	+	CR1	LINE
MLT1H1_dup242	9.87	6.25	1.25	5.00	5.6E-07	1.8E-04	chr1	222201493	222201997	1733	-	ERVL-MaLR	LTR
SVA_D_dup1318	10.01	6.26	1.25	5.00	5.7E-07	1.8E-04	chr20	32286284	32288520	6848	+	SVA_D	Other
SVA_E_dup190	10.79	6.33	1.27	5.00	5.7E-07	1.8E-04	chr16	31116139	31117030	2769	+	SVA_E	Other
AluY_dup73810	14.10	6.56	1.31	5.00	5.8E-07	1.8E-04	chr11	32509963	32510266	2570	-	Alu	SINE
MLT1D-int_dup611	12.48	6.45	1.29	5.00	5.8E-07	1.8E-04	chr21	29510956	29512501	3171	+	ERVL-MaLR	LTR
AluSx1_dup44119	23.25	-6.36	1.27	-4.99	5.9E-07	1.8E-04	chr7	146006612	146006918	2239	+	Alu	SINE
L1PA5_dup8976	9.26	6.19	1.24	4.99	6.0E-07	1.8E-04	chr12	95216596	95222788	25966	+	L1	LINE
L1PREC2_dup5573	13.96	6.55	1.31	4.99	6.1E-07	1.9E-04	chr11	6110824	6115074	21146	-	L1	LINE
AluSx3_dup17971	118.54	2.42	0.48	4.99	6.1E-07	1.9E-04	chr11	32626398	32626697	2360	-	Alu	SINE
L1MA2_dup4758	86.66	2.55	0.51	4.99	6.2E-07	1.9E-04	chrX	133694028	133694506	3436	-	L1	LINE
L1MDa_dup2577	52.26	-6.91	1.39	-4.99	6.2E-07	1.9E-04	chr6	134233584	134234839	7321	+	L1	LINE
L1PB4_dup5800	14.15	6.55	1.32	4.98	6.3E-07	1.9E-04	chr12	129570799	129571167	2024	-	L1	LINE
AluSp_dup39038	9.27	6.19	1.24	4.98	6.3E-07	1.9E-04	chr16	30120846	30121150	2354	+	Alu	SINE
L1MA7_dup8068	10.01	6.25	1.26	4.98	6.3E-07	1.9E-04	chr19	22143218	22143330	516	+	L1	LINE
L2c_dup11136	11.98	6.41	1.29	4.98	6.3E-07	1.9E-04	chr1	173629000	173629184	201	-	L2	LINE
AluJb_dup5484	19.02	6.80	1.36	4.98	6.4E-07	1.9E-04	chr1	58171975	58172284	1608	-	Alu	SINE
L1PA16_dup124	19.62	6.82	1.37	4.97	6.5E-07	2.0E-04	chr1	57907333	57907735	2431	-	L1	LINE
HERV3-int_dup263	471.93	6.57	1.32	4.97	6.6E-07	2.0E-04	chr19	24001565	24004302	15108	-	ERV1	LTR
L1ME4a_dup10673	17.79	6.74	1.36	4.97	6.6E-07	2.0E-04	chr4	3700864	3701050	266	+	L1	LINE
L1ME4a_dup35176	21.64	-6.28	1.26	-4.97	6.6E-07	2.0E-04	chr15	37314034	37314423	548	-	L1	LINE
L1PA2_dup4747	9.83	6.23	1.25	4.97	6.7E-07	2.0E-04	chr20	21891492	21897519	26140	-	L1	LINE
SVA_D_dup411	70.85	2.67	0.54	4.97	6.8E-07	2.0E-04	chr5	43521581	43523181	10023	+	SVA_D	Other
AluJo_dup63716	9.25	6.18	1.24	4.97	6.8E-07	2.0E-04	chr19	2881824	2882133	1619	+	Alu	SINE
SVA_D_dup745	9.98	6.25	1.26	4.97	6.8E-07	2.0E-04	chrX	48081188	48082652	11603	+	SVA_D	Other
MIR3_dup76946	756.56	-1.66	0.33	-4.96	6.9E-07	2.0E-04	chr16	28334360	28334422	251	-	MIR	SINE
L1PA5_dup8771	11.50	6.37	1.28	4.96	7.2E-07	2.1E-04	chr12	57367862	57368550	7254	+	L1	LINE
L2a_dup4797	15.16	6.60	1.33	4.95	7.2E-07	2.2E-04	chr1	58244687	58245225	775	-	L2	LINE
MADE1_dup5401	11.62	6.37	1.29	4.95	7.3E-07	2.2E-04	chr11	32519138	32519212	603	+	cMar-Marine	DNA
LTR12D_dup474	9.15	6.16	1.25	4.95	7.6E-07	2.2E-04	chr21	29256772	29258022	5524	-	ERV1	LTR
MER113A_dup1452	64.71	3.17	0.64	4.95	7.5E-07	2.2E-04	chr19	50816674	50816769	255	-	hAT-Charlie	DNA
L1ME3F_dup430	1741.11	-1.30	0.26	-4.95	7.6E-07	2.2E-04	chr1	238643892	238644423	685	+	L1	LINE
L1MC_dup8400	13.12	6.47	1.31	4.94	7.8E-07	2.3E-04	chr12	20331087	20333948	9788	-	L1	LINE
HERVKC4-int_dup32	10.85	6.31	1.28	4.94	7.9E-07	2.3E-04	chr19	7860947	7861799	5512	-	ERVK	LTR
L1PA7_dup12460	9.08	6.15	1.25	4.94	7.9E-07	2.3E-04	chr19	22165185	22166810	21600	+	L1	LINE
MIR_dup30035	25.34	-6.40	1.30	-4.94	8.0E-07	2.3E-04	chr3	10317707	10317925	354	+	MIR	SINE
L1MDa_dup3828	9.11	6.15	1.25	4.93	8.1E-07	2.4E-04	chr9	126674502	126675687	2773	+	L1	LINE
MER4A1_dup103	10.59	6.28	1.27	4.93	8.2E-07	2.4E-04	chr1	173643520	173643989	3083	-	ERV1	LTR
MER11D_dup107	10.00	6.23	1.27	4.93	8.4E-07	2.5E-04	chr7	63604327	63605215	7660	-	ERVK	LTR
L1PA4_dup3437	9.14	6.15	1.25	4.92	8.8E-07	2.6E-04	chr4	174524214	174530337	25731	-	L1	LINE
AluSq2_dup49932	9.33	6.17	1.25	4.91	8.9E-07	2.6E-04	chr19	22386402	22386694	2233	-	Alu	SINE
L1PA3_dup10248	24.52	5.77	1.17	4.91	8.9E-07	2.6E-04	chr19	29716687	29722722	28538	+	L1	LINE

AluSq2_dup51418	8.88	6.12	1.25	4.91	9.0E-07	2.6E-04	chr20	4015642	4015788	1100	+	Alu	SINE
L1PB1_dup1565	73.95	3.46	0.70	4.91	9.2E-07	2.7E-04	chr2	160784936	160786314	7292	+	L1	LINE
AluJb_dup5479	18.67	6.75	1.38	4.91	9.3E-07	2.7E-04	chr1	57907020	57907325	1839	-	Alu	SINE
L1PA5_dup5342	61.35	-3.35	0.68	-4.91	9.3E-07	2.7E-04	chr7	146002747	146006080	13150	+	L1	LINE
MER4A1_dup106	12.03	6.38	1.30	4.90	9.4E-07	2.7E-04	chr1	173680338	173680783	3434	-	ERV1	LTR
MLT1F2_dup3258	52.88	2.65	0.54	4.90	9.4E-07	2.7E-04	chrX	133693221	133693696	1733	+	ERVL-MaLR	LTR
MIR3_dup8870	842.28	-2.70	0.55	-4.90	9.4E-07	2.7E-04	chr1	245871952	245872114	577	+	MIR	SINE
MLT1J2_dup1399	9.32	6.16	1.26	4.90	9.5E-07	2.7E-04	chr3	110548351	110548754	754	-	ERVL-MaLR	LTR
AluJo_dup45143	236.72	-1.36	0.28	-4.90	9.5E-07	2.7E-04	chr11	125526111	125526254	935	+	Alu	SINE
MIRb_dup96994	19.34	-6.16	1.26	-4.90	9.6E-07	2.7E-04	chr7	101230412	101230556	350	+	MIR	SINE
L3_dup8410	28.28	-6.47	1.32	-4.90	9.7E-07	2.8E-04	chr3	10314912	10315147	427	+	CR1	LINE
MER63B_dup2197	10.53	6.27	1.28	4.90	9.7E-07	2.8E-04	chr19	7831924	7832112	602	-	ıAT-Blackjacl	DNA
AluJb_dup130606	131.88	3.20	0.65	4.89	9.9E-07	2.8E-04	chr19	17524027	17524187	1169	+	Alu	SINE
MER5B_dup24463	14.47	6.54	1.34	4.89	9.9E-07	2.8E-04	chr21	39617161	39617326	520	+	hAT-Charlie	DNA
L2c_dup138274	10.37	6.25	1.28	4.89	1.0E-06	2.8E-04	chr22	31973733	31974059	299	-	L2	LINE
SVA_A_dup72	10.58	6.27	1.28	4.89	1.0E-06	2.8E-04	chr5	133192865	133194574	8911	-	SVA_A	Other
Arthur1_dup239	12.70	6.43	1.31	4.89	1.0E-06	2.9E-04	chr2	42207931	42208277	2552	-	hAT-Tip100	DNA
L1ME5_dup352	12.01	6.38	1.31	4.88	1.0E-06	2.9E-04	chr2	42232932	42233556	1727	-	L1	LINE
L1PA3_dup1518	108.27	3.44	0.70	4.88	1.0E-06	2.9E-04	chr2	241483912	241489939	26513	+	L1	LINE
Tigger3b_dup93	15.11	6.57	1.34	4.88	1.0E-06	2.9E-04	chr1	57901021	57901576	6158	-	TcMar-Tigge1	DNA
L1PREC2_dup1084	189.39	2.80	0.57	4.88	1.1E-06	3.0E-04	chr2	231409494	231410269	10157	+	L1	LINE
LTR84b_dup945	10.20	6.23	1.28	4.88	1.1E-06	3.0E-04	chr12	129561835	129562060	293	+	ERVL	LTR
MIR3_dup16630	18.81	-6.13	1.26	-4.88	1.1E-06	3.0E-04	chr3	10329830	10329909	240	+	MIR	SINE
L1P2_dup1028	19.35	5.52	1.13	4.87	1.1E-06	3.0E-04	chrX	130789937	130793165	12257	+	L1	LINE
MIRc_dup90505	26.94	5.72	1.17	4.87	1.1E-06	3.0E-04	chr17	5440840	5440959	200	+	MIR	SINE
L1M5_dup24956	87.20	-5.22	1.07	-4.87	1.1E-06	3.1E-04	chr6	134226080	134226149	260	-	L1	LINE
L1PA15_dup7636	9.14	6.13	1.26	4.87	1.1E-06	3.1E-04	chr17	5466052	5467347	6814	+	L1	LINE
L1PA5_dup10887	11.38	6.33	1.30	4.87	1.1E-06	3.1E-04	chr20	18227354	18229727	13224	+	L1	LINE
HERVK9-int_dup586	15.88	6.60	1.36	4.87	1.1E-06	3.1E-04	chr19	9822698	9827591	40634	-	ERVK	LTR
L1PA3_dup7486	10.36	6.24	1.28	4.87	1.1E-06	3.1E-04	chr10	54490116	54496287	26920	-	L1	LINE
L1ME2_dup1944	26.06	-6.39	1.31	-4.87	1.1E-06	3.1E-04	chr2	181753903	181755601	4730	+	L1	LINE
L1MB8_dup3926	10.38	6.24	1.28	4.86	1.1E-06	3.1E-04	chr4	89343276	89344376	2474	-	L1	LINE
MER11B_dup493	10.20	6.23	1.28	4.86	1.1E-06	3.1E-04	chr15	89477380	89478465	6391	-	ERVK	LTR
MER91B_dup752	132.45	-2.04	0.42	-4.86	1.2E-06	3.2E-04	chr8	63894853	63894984	286	-	hAT-Tip100	DNA
MER5B_dup15659	299.53	-2.32	0.48	-4.86	1.2E-06	3.2E-04	chr10	61411203	61411372	350	+	hAT-Charlie	DNA
L1PA3_dup7758	9.05	6.12	1.26	4.86	1.2E-06	3.2E-04	chr11	26472643	26478780	26895	+	L1	LINE
L2a_dup145210	203.17	-1.43	0.29	-4.86	1.2E-06	3.2E-04	chr16	30410634	30410702	349	+	L2	LINE
AluSq_dup16072	46.04	-6.75	1.39	-4.86	1.2E-06	3.2E-04	chr15	37316113	37316398	2130	-	Alu	SINE
Charlie2a_dup1143	83.93	-2.36	0.49	-4.86	1.2E-06	3.2E-04	chr8	63858005	63858284	595	-	hAT-Charlie	DNA
MIR_dup42027	13.37	6.45	1.33	4.86	1.2E-06	3.3E-04	chr4	10017894	10018096	691	+	MIR	SINE
THE1D_dup1529	27.51	-6.43	1.32	-4.85	1.2E-06	3.3E-04	chr2	181783338	181783711	1901	-	ERVL-MaLR	LTR
SVA_D_dup1000	48.42	4.27	0.88	4.85	1.3E-06	3.4E-04	chr13	52653457	52655042	10417	+	SVA_D	Other

L2c_dup16809	31.95	-4.35	0.90	-4.84	1.3E-06	3.4E-04	chr2	21243717	21244822	649	+	L2	LINE
L1MB1_dup6233	68.22	-2.58	0.53	-4.84	1.3E-06	3.4E-04	chr7	153539	154348	704	-	L1	LINE
MIR_dup59489	106.01	1.65	0.34	4.84	1.3E-06	3.5E-04	chr5	140601985	140602190	545	-	MIR	SINE
L1PA16_dup11258	61.04	-6.82	1.41	-4.84	1.3E-06	3.5E-04	chr13	39141284	39143543	18290	+	L1	LINE
L1MC3_dup9745	10.93	-5.64	1.17	-4.84	1.3E-06	3.6E-04	chr12	125273626	125273967	1552	+	L1	LINE
L1PA7_dup2458	9.85	6.19	1.28	4.83	1.3E-06	3.6E-04	chr3	110595585	110595818	1778	-	L1	LINE
L1PA13_dup229	39.49	-6.65	1.38	-4.83	1.3E-06	3.6E-04	chr1	143162529	143165060	9314	-	L1	LINE
L1P1_dup1143	8.63	6.07	1.26	4.83	1.4E-06	3.6E-04	chr6	46050973	46054126	17149	-	L1	LINE
L1ME3_dup7717	13.53	6.45	1.34	4.83	1.4E-06	3.6E-04	chr16	56771399	56771668	1297	-	L1	LINE
L1M5_dup53189	12.47	6.38	1.32	4.83	1.4E-06	3.6E-04	chr15	74062691	74063189	685	-	L1	LINE
L1M3_dup1329	9.35	6.14	1.27	4.83	1.4E-06	3.7E-04	chr4	1133589	1134597	7829	+	L1	LINE
L1ME3F_dup1954	39.85	-6.65	1.38	-4.83	1.4E-06	3.7E-04	chr6	134249560	134250073	1151	-	L1	LINE
L1MEf_dup11908	31.68	-6.51	1.35	-4.83	1.4E-06	3.7E-04	chr16	32266842	32266927	407	-	L1	LINE
L1MEf_dup9416	8.79	6.08	1.26	4.83	1.4E-06	3.7E-04	chr11	45135638	45136692	3491	+	L1	LINE
AluSz_dup94203	8.90	6.09	1.26	4.82	1.4E-06	3.7E-04	chr20	62441840	62442127	2038	+	Alu	SINE
AluJr4_dup6719	61.43	7.57	1.57	4.82	1.4E-06	3.7E-04	chr6	132554455	132554693	1203	-	Alu	SINE
MER57A-int_dup583	9.76	6.17	1.28	4.82	1.4E-06	3.7E-04	chr5	152104668	152107245	10885	+	ERV1	LTR
L1PA3_dup1305	185.29	3.99	0.83	4.82	1.4E-06	3.7E-04	chr2	174392018	174398045	28244	-	L1	LINE
L1PA7_dup12459	28.93	5.53	1.15	4.82	1.4E-06	3.7E-04	chr19	22160086	22164877	21600	+	L1	LINE
L2a_dup29737	11.60	6.32	1.31	4.82	1.5E-06	3.8E-04	chr3	13666786	13667787	1327	+	L2	LINE
L2b_dup91226	10.40	6.23	1.29	4.82	1.5E-06	3.8E-04	chr19	50822736	50823077	425	+	L2	LINE
BLACKJACK_dup2040	45.48	2.92	0.61	4.82	1.5E-06	3.8E-04	chr19	40563857	40564624	1904	-	ıAT-Blackjacl	DNA
MIRc_dup47827	1602.61	-0.88	0.18	-4.81	1.5E-06	3.9E-04	chr8	26514443	26514531	181	-	MIR	SINE
BSR/Beta_dup1590	34.16	5.57	1.16	4.81	1.5E-06	3.9E-04	chr19	22090091	22094452	1565	-	Satellite	Satellite
MER66-int_dup697	12.29	6.36	1.32	4.81	1.5E-06	4.0E-04	chr19	53553422	53553989	1137	+	ERV1	LTR
L1M2_dup7890	33.74	-6.53	1.36	-4.81	1.5E-06	4.0E-04	chr16	32262347	32263461	3396	+	L1	LINE
AluSx1_dup10800	42.96	2.39	0.50	4.80	1.6E-06	4.0E-04	chr2	54077536	54077832	2290	+	Alu	SINE
L2a_dup21475	10.78	-5.62	1.17	-4.80	1.6E-06	4.2E-04	chr2	105764370	105764472	274	-	L2	LINE
LTR12D_dup432	22.28	-6.23	1.30	-4.80	1.6E-06	4.2E-04	chr15	81706223	81707427	7383	+	ERV1	LTR
MLT1C_dup14475	8.88	6.08	1.27	4.80	1.6E-06	4.2E-04	chr13	52660775	52661229	2135	+	ERVL-MaLR	LTR
L1PA4_dup9906	29.30	4.03	0.84	4.79	1.6E-06	4.2E-04	chr13	52223889	52228685	21140	-	L1	LINE
L1MB2_dup7619	9.20	6.11	1.28	4.79	1.7E-06	4.3E-04	chr17	56641201	56642047	5041	+	L1	LINE
L1PA7_dup1298	49.96	4.93	1.03	4.79	1.7E-06	4.3E-04	chr2	113615081	113616060	9888	+	L1	LINE
HERVIP10F-int_dup356	90.63	4.15	0.87	4.79	1.7E-06	4.3E-04	chr16	30108529	30110361	3397	+	ERV1	LTR
MIRc_dup75107	13.97	-5.84	1.22	-4.78	1.7E-06	4.4E-04	chr12	57976663	57976802	319	+	MIR	SINE
MIRb_dup121667	217.10	3.36	0.70	4.78	1.8E-06	4.6E-04	chr9	127575625	127575780	399	-	MIR	SINE
L2a_dup72421	49.64	-2.25	0.47	-4.78	1.8E-06	4.6E-04	chr6	165802351	165804237	1159	-	L2	LINE
L1M4_dup1698	9.90	6.17	1.29	4.77	1.8E-06	4.6E-04	chr2	102775750	102776517	1716	-	L1	LINE
MER34B_dup164	11.01	6.26	1.31	4.77	1.8E-06	4.7E-04	chr4	9863641	9864194	3476	+	ERV1	LTR
L1PREC2_dup5413	22.71	-6.24	1.31	-4.77	1.9E-06	4.7E-04	chr10	56074306	56075610	5648	-	L1	LINE
Charlie9_dup60	10.78	6.24	1.31	4.77	1.9E-06	4.7E-04	chr1	173633136	173633232	327	-	hAT-Charlie	DNA
L1PA7_dup1299	46.18	5.92	1.24	4.77	1.9E-06	4.7E-04	chr2	113616367	113617505	9888	+	L1	LINE

L1M4b_dup2581	8.42	6.02	1.26	4.76	1.9E-06	4.8E-04	chr7	63627177	63628016	1659	+	L1	LINE
L1MC3_dup13006	9.23	6.10	1.28	4.76	1.9E-06	4.9E-04	chr21	29416161	29418216	5701	+	L1	LINE
L1PA8A_dup1948	12.58	6.36	1.34	4.76	1.9E-06	4.9E-04	chr12	20219416	20220641	13259	+	L1	LINE
L1PA3_dup9524	36.82	5.64	1.19	4.76	1.9E-06	4.9E-04	chr15	53976397	53982426	27413	+	L1	LINE
HERVL40-int_dup1204	8.71	6.05	1.27	4.76	1.9E-06	4.9E-04	chr19	9574658	9575313	1829	-	ERVL	LTR
AluSq2_dup10194	12.53	-5.74	1.21	-4.76	2.0E-06	4.9E-04	chr3	111681496	111681804	2303	+	Alu	SINE
SVA_D_dup539	49.29	5.70	1.20	4.76	2.0E-06	4.9E-04	chr6	151770765	151772353	10263	+	SVA_D	Other
MER63B_dup2030	9.03	6.08	1.28	4.76	2.0E-06	4.9E-04	chr16	30117460	30117745	1462	+	ıAT-Blackjacl	DNA
SVA_D_dup918	8.94	6.07	1.28	4.76	2.0E-06	4.9E-04	chr12	12718331	12719858	10901	+	SVA_D	Other
L1MB3_dup12548	27.47	5.61	1.18	4.76	2.0E-06	4.9E-04	chr13	52709676	52709817	722	+	L1	LINE
MIRc_dup96829	79.38	3.97	0.84	4.76	2.0E-06	4.9E-04	chr19	34398269	34398468	372	+	MIR	SINE
HERVIP10F-int_dup184	119.91	7.65	1.61	4.76	2.0E-06	4.9E-04	chr7	121148081	121151020	9498	-	ERV1	LTR
AluSx_dup62709	8.78	6.06	1.27	4.75	2.0E-06	5.0E-04	chr8	124220954	124221221	1505	+	Alu	SINE
AluSg_dup5064	12.21	6.33	1.33	4.75	2.0E-06	5.1E-04	chr2	113613582	113613868	2302	-	Alu	SINE
L1PBa_dup2131	9.62	6.13	1.29	4.75	2.1E-06	5.1E-04	chr20	18219793	18221232	9950	+	L1	LINE
L1MB1_dup1023	14.72	-5.87	1.24	-4.75	2.1E-06	5.1E-04	chr3	115639412	115641599	3850	-	L1	LINE
MLT1J_dup839	9.75	6.14	1.29	4.75	2.1E-06	5.1E-04	chr1	173632702	173632878	309	+	ERVL-MaLR	LTR
L1M5_dup8639	19.65	-6.11	1.29	-4.75	2.1E-06	5.1E-04	chr2	181622664	181623015	1096	-	L1	LINE
MIRc_dup3661	12.66	6.36	1.34	4.74	2.1E-06	5.1E-04	chr1	58021331	58021456	356	-	MIR	SINE
HERVI-int_dup131	47.50	5.64	1.19	4.74	2.1E-06	5.2E-04	chr18	44448764	44450296	30365	+	ERV1	LTR
L1MA9_dup105	11.56	6.29	1.33	4.74	2.1E-06	5.2E-04	chr1	57894858	57895930	5094	-	L1	LINE
L2a_dup35572	9.42	6.11	1.29	4.74	2.1E-06	5.2E-04	chr3	110531002	110531965	542	+	L2	LINE
AluSz_dup58734	31.91	5.96	1.26	4.74	2.1E-06	5.2E-04	chr11	32510735	32511045	2317	-	Alu	SINE
L1MCa_dup1345	13.21	6.39	1.35	4.74	2.1E-06	5.2E-04	chr3	110681644	110681986	567	+	L1	LINE
L2b_dup30108	9.73	6.14	1.30	4.73	2.2E-06	5.4E-04	chr5	43523211	43523924	571	-	L2	LINE
SVA_A_dup236	9.93	6.15	1.30	4.73	2.2E-06	5.4E-04	chr19	21441697	21443275	6481	-	SVA_A	Other
AluSx3_dup1810	13.55	6.41	1.36	4.73	2.2E-06	5.4E-04	chr1	173636561	173636870	2221	+	Alu	SINE
L2c_dup98246	9.09	6.08	1.29	4.73	2.3E-06	5.5E-04	chr11	86594929	86595153	180	+	L2	LINE
LTR77_dup7	100.25	2.10	0.44	4.73	2.3E-06	5.5E-04	chr1	110276470	110277100	3248	-	ERV1	LTR
MER34A1_dup352	33.43	-6.48	1.37	-4.72	2.3E-06	5.6E-04	chr7	94337294	94337863	3194	-	ERV1	LTR
LOR1-int_dup297	23.48	2.52	0.53	4.72	2.3E-06	5.7E-04	chr2	241489976	241490877	5338	+	ERV1	LTR
MIRc_dup10449	543.89	-2.28	0.48	-4.72	2.4E-06	5.8E-04	chr1	245871372	245871610	352	+	MIR	SINE
L1PREC2_dup6941	992.04	4.03	0.85	4.72	2.4E-06	5.8E-04	chr17	6676620	6677897	8597	+	L1	LINE
MER107_dup76	79.06	2.64	0.56	4.72	2.4E-06	5.8E-04	chr4	89427037	89427056	1018	-	DNA	DNA
MIRb_dup10577	11.33	-5.63	1.19	-4.71	2.4E-06	5.8E-04	chr1	64935176	64935382	601	+	MIR	SINE
Tigger1_dup7856	8.41	6.00	1.27	4.71	2.5E-06	5.9E-04	chr10	11104062	11105209	13909	+	TcMar-Tigger	DNA
L1MD2_dup4617	66.31	7.48	1.59	4.71	2.5E-06	5.9E-04	chr7	121133311	121133757	1690	-	L1	LINE
L1HS_dup187	11.34	6.26	1.33	4.71	2.5E-06	5.9E-04	chr2	160971028	160977191	25552	+	L1	LINE
L1M1_dup1122	40.30	-6.58	1.40	-4.71	2.5E-06	6.0E-04	chr2	186459226	186460301	3327	+	L1	LINE
AluJo_dup10114	44.51	-6.63	1.41	-4.70	2.6E-06	6.1E-04	chr2	186504842	186505112	1463	+	Alu	SINE
MIR3_dup8869	15.85	-5.91	1.26	-4.69	2.7E-06	6.4E-04	chr1	245871889	245871926	230	+	MIR	SINE
MSTA-int_dup561	8.85	6.04	1.29	4.69	2.7E-06	6.5E-04	chr3	110669239	110670791	9196	+	ERVL-MaLR	LTR

AluY_dup3561	12.16	6.30	1.34	4.69	2.8E-06	6.6E-04	chr1	58107039	58107325	2297	-	Alu	SINE
AluSg_dup30899	38.36	-6.54	1.40	-4.69	2.8E-06	6.7E-04	chr15	37214809	37215116	2098	-	Alu	SINE
AluSx_dup5273	11.92	6.29	1.34	4.68	2.8E-06	6.7E-04	chr1	58466931	58467230	2316	-	Alu	SINE
L1PA3_dup1573	227.45	6.13	1.31	4.68	2.8E-06	6.7E-04	chr3	11526024	11528297	12604	-	L1	LINE
L2a_dup145190	17.23	5.36	1.14	4.68	2.8E-06	6.7E-04	chr16	30115556	30115634	215	+	L2	LINE
AluSx3_dup26678	8.87	6.04	1.29	4.68	2.9E-06	6.8E-04	chr19	22122828	22122921	644	+	Alu	SINE
HERV9-int_dup740	12.58	6.33	1.35	4.68	2.9E-06	6.9E-04	chr10	5097054	5101973	40652	-	ERV1	LTR
SVA_F_dup3	38.26	5.21	1.12	4.68	2.9E-06	6.9E-04	chr1	21349733	21351405	9800	+	SVA_F	Other
L1MD_dup2334	14.05	6.42	1.37	4.67	3.0E-06	7.0E-04	chr4	166568170	166569111	2857	+	L1	LINE
L1PA15_dup999	8.05	5.95	1.27	4.67	3.0E-06	7.0E-04	chr2	174590743	174592574	6706	+	L1	LINE
L1MEf_dup11909	34.73	-6.47	1.39	-4.67	3.0E-06	7.1E-04	chr16	32266962	32267316	530	-	L1	LINE
MER50_dup103	11.25	6.23	1.33	4.67	3.0E-06	7.1E-04	chr1	173639770	173639983	3716	+	ERV1	LTR
SVA_D_dup1052	9.39	6.08	1.30	4.67	3.0E-06	7.1E-04	chr14	92022875	92024752	8575	-	SVA_D	Other
L1P1_dup2760	38.78	2.51	0.54	4.67	3.1E-06	7.1E-04	chr15	43467899	43469402	12154	+	L1	LINE
AluSq2_dup29796	210.16	-1.22	0.26	-4.67	3.1E-06	7.2E-04	chrX	135930461	135930772	2467	+	Alu	SINE
HERVH-int_dup1256	11.07	6.22	1.33	4.67	3.1E-06	7.2E-04	chr3	128682088	128682569	3109	+	ERV1	LTR
L2b_dup10603	100.20	-4.31	0.92	-4.66	3.1E-06	7.2E-04	chr2	14779882	14779907	183	+	L2	LINE
L1PA2_dup412	23.96	5.52	1.18	4.66	3.1E-06	7.3E-04	chr2	72291106	72297127	28214	+	L1	LINE
LOR1-int_dup1347	10.67	6.19	1.33	4.66	3.1E-06	7.3E-04	chr20	31704293	31705397	5960	-	ERV1	LTR
AluJo_dup29470	11.42	-5.62	1.21	-4.66	3.1E-06	7.3E-04	chr8	6211170	6211480	2100	+	Alu	SINE
L1PA5_dup10346	69.21	-2.48	0.53	-4.66	3.2E-06	7.4E-04	chr17	31277436	31277978	4719	-	L1	LINE
L1PA6_dup3010	66.35	-2.20	0.47	-4.65	3.2E-06	7.5E-04	chr8	70775813	70779220	23707	+	L1	LINE
SVA_F_dup980	8.88	6.03	1.30	4.65	3.2E-06	7.5E-04	chr22	31989136	31989973	5415	-	SVA_F	Other
L1PA6_dup3468	8.21	5.96	1.28	4.65	3.3E-06	7.5E-04	chr9	134214449	134217333	23524	-	L1	LINE
L2_dup46255	11.25	6.22	1.34	4.65	3.3E-06	7.6E-04	chr14	56437450	56438020	911	+	L2	LINE
L1PA16_dup5746	55.19	7.37	1.58	4.65	3.3E-06	7.6E-04	chr6	132525262	132532490	20047	-	L1	LINE
L1PB1_dup9922	152.77	-3.75	0.81	-4.65	3.3E-06	7.7E-04	chr11	100199722	100205901	21154	+	L1	LINE
MLT1B_dup8067	55.70	7.36	1.59	4.64	3.4E-06	7.9E-04	chr7	121126286	121126713	1569	-	ERVL-MaLR	LTR
MER45R_dup262	10.25	6.14	1.32	4.64	3.4E-06	7.9E-04	chr7	85257259	85258812	4376	+	hAT-Tip100	DNA
LTR16A_dup4855	11.01	6.20	1.34	4.64	3.5E-06	7.9E-04	chr11	32517272	32517684	1037	+	ERVL	LTR
L1PA3_dup8847	28.13	5.01	1.08	4.64	3.5E-06	8.0E-04	chr13	46238200	46244348	27756	-	L1	LINE
Kanga1_dup103	19.46	-6.06	1.31	-4.64	3.5E-06	8.1E-04	chr2	181831975	181832129	736	-	TcMar-Tc2	DNA
L1MC4_dup27051	8.12	5.94	1.28	4.64	3.6E-06	8.1E-04	chr19	22380535	22380742	423	+	L1	LINE
AluSz_dup88689	165.05	2.72	0.59	4.63	3.6E-06	8.2E-04	chr19	17525396	17525697	2312	+	Alu	SINE
L1M6_dup1436	11.54	6.24	1.35	4.63	3.6E-06	8.3E-04	chr3	134716158	134717121	1114	-	L1	LINE
MIRb_dup220646	43.80	2.11	0.46	4.63	3.7E-06	8.3E-04	chr22	29657014	29657116	221	-	MIR	SINE
HERVIP10F-int_dup152	11.26	6.21	1.34	4.63	3.7E-06	8.4E-04	chr6	118896757	118897394	2274	+	ERV1	LTR
SVA_E_dup44	8.21	5.95	1.29	4.63	3.7E-06	8.4E-04	chr2	105826122	105828425	6493	-	SVA_E	Other
L1PA4_dup1944	29.70	2.58	0.56	4.63	3.7E-06	8.4E-04	chr3	43720447	43726586	26376	+	L1	LINE
Charlie19a_dup1004	8.35	5.96	1.29	4.62	3.8E-06	8.6E-04	chr10	80220413	80220676	637	-	hAT-Charlie	DNA
L1ME1_dup6445	9.62	6.08	1.32	4.62	3.8E-06	8.6E-04	chr3	110537347	110537824	6803	+	L1	LINE
MLT1J_dup14839	60.72	7.36	1.59	4.62	3.9E-06	8.7E-04	chr21	29542462	29542669	442	-	ERVL-MaLR	LTR

SVA_F_dup865	25.34	5.42	1.17	4.62	3.9E-06	8.8E-04	chr17	41124316	41126028	10217	+	SVA_F	Other
L1HS_dup775	28.07	-6.31	1.37	-4.62	3.9E-06	8.8E-04	chr7	124819625	124822032	18089	-	L1	LINE
SVA_F_dup826	8.20	5.94	1.29	4.61	3.9E-06	8.9E-04	chr16	28427206	28429159	7963	+	SVA_F	Other
LTR89_dup850	9.58	6.08	1.32	4.61	4.0E-06	9.0E-04	chr13	31409975	31410254	606	-	ERVL	LTR
L2a_dup25331	19.62	-6.05	1.31	-4.61	4.0E-06	9.0E-04	chr2	181773186	181773564	674	-	L2	LINE
MamGypLTR1b_dup109	1538.25	-1.92	0.42	-4.61	4.0E-06	9.0E-04	chr16	84599853	84599987	232	+	Gypsy	LTR
AluSc_dup31282	77.05	3.12	0.68	4.61	4.1E-06	9.1E-04	chr19	17530404	17530530	997	-	Alu	SINE
HAL1_dup4782	70.65	-5.13	1.11	-4.61	4.1E-06	9.2E-04	chr3	10304883	10306197	3371	-	L1	LINE
MamGypLTR1a_dup793	31.41	-6.37	1.38	-4.61	4.1E-06	9.2E-04	chr11	99527628	99527722	247	+	Gypsy	LTR
L1PA3_dup947	7.81	5.90	1.28	4.61	4.1E-06	9.2E-04	chr2	60416159	60422188	27994	+	L1	LINE
L1HS_dup298	8.66	5.99	1.30	4.60	4.1E-06	9.2E-04	chr3	120291869	120298033	29375	+	L1	LINE
L2c_dup60080	63.48	3.01	0.65	4.60	4.2E-06	9.3E-04	chr7	938017	938107	183	+	L2	LINE
MER74A_dup336	11.13	6.19	1.35	4.60	4.2E-06	9.3E-04	chr3	110524488	110525027	2715	-	ERVL	LTR
MIRb_dup153739	44.31	-6.54	1.42	-4.60	4.2E-06	9.3E-04	chr11	99200435	99200590	280	+	MIR	SINE
L1ME3F_dup294	11.14	6.19	1.35	4.60	4.2E-06	9.3E-04	chr1	173630126	173630353	273	+	L1	LINE
L1ME1_dup21403	9.10	6.03	1.31	4.60	4.3E-06	9.4E-04	chr11	32548018	32548319	1743	+	L1	LINE
L1PB1_dup11584	34.85	-4.60	1.00	-4.60	4.3E-06	9.4E-04	chr15	49200062	49206220	24315	-	L1	LINE
Charlie16a_dup1727	24.74	-6.22	1.35	-4.60	4.3E-06	9.4E-04	chr16	32266306	32266361	234	+	hAT-Charlie	DNA
HERV16-int_dup1361	10.41	6.14	1.34	4.60	4.3E-06	9.5E-04	chr12	10026562	10027511	2818	+	ERVL	LTR
HAL1_dup4167	18.79	-6.01	1.31	-4.59	4.3E-06	9.5E-04	chr2	181763953	181764881	2646	+	L1	LINE
L2c_dup93144	18.79	-6.01	1.31	-4.59	4.3E-06	9.5E-04	chr11	4681757	4681882	262	+	L2	LINE
AluSx_dup22458	18.43	-6.00	1.31	-4.59	4.4E-06	9.5E-04	chr3	10313008	10313287	2079	-	Alu	SINE
MER94_dup237	10.77	6.16	1.34	4.59	4.4E-06	9.7E-04	chr1	173633328	173633456	417	+	ɿAT-Blackjacl	DNA
SVA_F_dup944	8.60	5.98	1.30	4.59	4.5E-06	9.7E-04	chr20	30763175	30764214	5831	+	SVA_F	Other
L1PA7_dup3489	8.33	5.95	1.30	4.59	4.5E-06	9.7E-04	chr4	111347353	111353784	21782	-	L1	LINE
AluSx1_dup16861	17.94	-5.97	1.30	-4.59	4.5E-06	9.7E-04	chr3	10316674	10316960	2133	-	Alu	SINE
MLT1G1_dup146	69.56	-3.33	0.73	-4.59	4.5E-06	9.8E-04	chr1	173575137	173575648	1882	-	ERVL-MaLR	LTR
Charlie18a_dup176	71.99	-3.21	0.70	-4.59	4.5E-06	9.8E-04	chr1	173575736	173575967	772	+	hAT-Charlie	DNA
MIR_dup95045	11.39	6.21	1.35	4.59	4.5E-06	9.8E-04	chr9	126430667	126430916	698	-	MIR	SINE
HERVIP10F-int_dup279	8.29	5.94	1.30	4.59	4.5E-06	9.8E-04	chr11	69885233	69886572	3077	+	ERV1	LTR
MER51A_dup320	47.41	5.51	1.20	4.58	4.6E-06	9.9E-04	chr6	118912357	118913000	5024	+	ERV1	LTR
L1PA3_dup4872	7.80	5.89	1.29	4.58	4.6E-06	9.9E-04	chr7	97161884	97165015	25832	+	L1	LINE
MER58B_dup966	25.74	-6.23	1.36	-4.58	4.6E-06	9.9E-04	chr2	186699848	186700149	1117	-	hAT-Charlie	DNA
L1MC5_dup11521	245.47	-2.99	0.65	-4.58	4.6E-06	1.0E-03	chrX	51232874	51233148	1110	-	L1	LINE
L2c_dup4587	11.66	6.22	1.36	4.58	4.6E-06	1.0E-03	chr1	58571573	58571685	348	-	L2	LINE
