# Supplementary material for: A unique subset of low-risk Wilms tumors is characterized by loss of function of TRIM28 (KAP1), a gene critical in early renal development: A Children’s Oncology Group study
Source: PLoS One. 2018 Dec 13;13(12):e0208936. doi: 10.1371/journal.pone.0208936 (PMC6292605; doi:10.1371/journal.pone.0208936)
Supplement: S1 File — (DOCX) [file pone.0208936.s009.docx]

**A unique subset of low-risk Wilms tumors is characterized by loss of function of *TRIM28 (KAP1)*, a gene critical in early renal development: A Children’s Oncology Group study.**

Amy E. Armstrong, Samantha Gadd, Vicki Huff, Daniela S. Gerhard, Jeffrey S. Dome, Elizabeth J. Perlman

**Supplemental Methods**

***Analysis of Previous TARGET data***

Whole exome sequencing (WES) and RNA sequencing (RNAseq) performed on two S1 tumors included in the TARGET initiative were acquired through dbGAP study accession (Specimen IDs provided below)

| Sample | NCBI SRA ID  Tumor WES | NCBI SRA ID  Normal WES | NCBI SRA ID  Tumor RNAseq |
| --- | --- | --- | --- |
| PAJMKN | SRR566158 | SRR566160 | SRR1797028 |
| PAKVET | SRR566205 | SRR566187 | SRR1796953 |
| CAAAAQ FHWT (TRIM28 wt) |  |  | SRR1329251 |
| CAAAAR FHWT (TRIM28 wt) |  |  | SRR1274446 |
| PAJNRL FHWT (TRIM28 wt) |  |  | SRR1285638 |
| PAJNSL FHWT (TRIM28 wt) |  |  | SRR1274470 |
| PAJPHA FHWT (TRIM28 wt) |  |  | SRR1274472 |
| PALKCW FHWT (TRIM28 wt) |  |  | SRR1285639 |
| PAKYLT  Anaplastic WT with TRIM28 mutation |  |  | SRR1796977 |

***Copy number analysis***

Copy number analysis was performed using the Illumina Human 610-quad beadchip. The dat files were processed in BeadStudio (Illumina, San Diego, CA) and the output files were imported into Nexus 6.1 (BioDiscovery, El Segundo, CA). In Nexus 6.1, the data were segmented using SNP-FASST2 Segmentation with a significance threshold of 1.0e^-07^, a maximum contiguous probe spacing of 1000 Kbp and a minimum number of probes per segment of 8. The following parameters were used: segmented regions with calls greater than 0.2 or less than -0.23 were called as gain or loss, respectively; regions of minimum length = 500 kb in which the allelic ratio was ≥ 0.8 in ≥ 95% of probes were called as LOH; the heterozygous imbalance threshold was set at 0.4; and the minimum SNP probe density (probes/Mb) was set at 0. Regions that overlapped ≥ 85% with a known region of copy number variation in the general population based on The Centre for Applied Genomics’ Database of Genomic Variants (http://dgv.tcag.ca/dgv/) were removed.

***Sanger Sequencing:***

| Variant and PCR conditions | Forward Primer | Reverse primer |
| --- | --- | --- |
| 59059081G>A ^a^ | 5’-AGGATGCAGTGAGGAACCAG-3’ | 5’-GGCCATCTTGACATCCACTT-3’ |
| 59056439_59056440  insCGGCGGGG ^b^ | 5’-GCCTGTAGTGCCTGCTTAGG-3’ | 5’-ACAAGTTCCCACCCAGGTTA-3’ |
| 59056466T>G ^b^ | 5’-GCCTGTAGTGCCTGCTTAGG-3’ | 5’-ACAAGTTCCCACCCAGGTTA-3’ |
| ERV1  (LTR4_dup91) ^c^ | 5’-TACTTGCACCACGAGCTTTG-3’ | 5’-GAGGCAGGAGACACCCAGTA-3’ |
| ERV2  (HERVIP10F-int_dup359) ^c^ | 5’-CCACCAAATCAAACCATTCC-3’ | 5’-CCGCTGCACAAGAGTAACAA-3’ |
| ERV3  (HERV3-int_dup262)  ^c^ | 5’-AATGAATGGCCACCTGAAAG-3’ | 5’-TCCGAGCCAGCAAATTTAAG-3’ |
| ERV4  (MLT2B4_dup580) c | 5’-CAAATGGAAACATCGGCTTT-3’ | 5’-TCCCAATTTCTGCATCAACA-3’ |

1. PCR amplification was performed using Platinum PCR SuperMix High Fidelity (Life Technologies Corporation, Carlsbad, CA) with the following cycling conditions: initial denaturation at 94°C for 5 min; 35 cycles at 94°C for 30 s, 58°C for 1 min, 72°C for 1 min; a final extension at 72°C for 8 min.
2. The Invitrogen PCRx Enhancer System (Thermo Fisher Scientific, Waltham, MA) was utilized for this GC-rich region with a 4X enhancer concentration. Cycling conditions were: initial denaturation at 94°C for 5 min; 35 cycles at 94°C for 30 s, 50°C for 1 min, 72°C for 1 min; a final extension at 72°C for 8 min.
3. The reverse transcriptase reaction was performed using the High-Capacity cDNA Reverse Transcription Kit (Applied Biosciences, Foster City, CA) with the following conditions: 25°C for 10 min, 37°C for 120 min, and 85°C for 5 min. PCR was performed using the SYBR Green PCR Kit (Applied Biosystems) with the following conditions: activation at 95°C for 10 min followed by 40 cycles of 95°C for 15 sec and 60°C for 1 min. Primers were generated using Primer3 (Koressaar et al 2007).

***CRISPR***

Puromycin was added 24 hours after transfection for 72 hours to select cells expressing the Cas9-puromycin-resistance expression plasmid. Genomic DNA was isolated from selected cells, and CRISPR editing was confirmed using the Surveyor Kit (IDT, Skokie IL). Following expansion of individual clones, *TRIM28* gene and protein expression were compared to parent HEK293 cells by RT-PCR (Life Technologies, Carlsbad, CA) and by western blotting with TRIM28 polyclonal antibody (Abcam, Cambridge MA). The following clones were submitted for Sanger sequencing to evaluate the CRISPR-targeted region (GeneWiz): the parent HEK293 cell line, four clones with very low TRIM28 RNA and protein expression, and two clones with wildtype TRIM28 RNA and protein expression. The following primers were used: forward 5’-CTTCTCTGACCCTGCCTTTG-3’ and reverse 5’-CACCAGATGCAACCAACATC-3’. This revealed a homozygous frameshift mutation at p.Val192 in 3/4 clones showing very low TRIM28 RNA and protein expression; the fourth clone had a heterozygous mutation. The mutation was absent and no other abnormalities were detected in the parent cell line and in two clones with normal TRIM28 RNA and protein levels.

***References***

Katz Y, Wang ET, Silterra J, Schwartz S, Wong B, Thorvaldsdottir H, Robinson JT, Mesirov JP, Airoldi EM, Burge CB. Quantitative visualization of alternative exon expression from RNA-seq data. 2015 Bioinformatics Jul 15;31(14):2400-2.

Koressaar T, Remm M. Enhancements and modifications of primer design program Primer3. 2007 Bioinformatics May 15;23(10):1289-91.
